# A Web-Based Intervention to Support the Mental Well-Being of Sexual and Gender Minority Young People: Mixed Methods Co-Design of Oneself

**DOI:** 10.2196/54586

**Published:** 2024-05-21

**Authors:** Katherine Brown, Mathijs F G Lucassen, Alicia Núñez-García, Katharine A Rimes, Louise M Wallace, Rajvinder Samra

**Affiliations:** 1 Centre for Research in Psychology and Sports Science School of Life and Medical Sciences University of Hertfordshire Hatfield United Kingdom; 2 School of Health & Psychological Sciences City, University of London London United Kingdom; 3 School of Health, Wellbeing & Social Care The Open University Milton Keynes United Kingdom; 4 Institute of Psychiatry, Psychology & Neuroscience Kings College London London United Kingdom

**Keywords:** sexual minority, gender minority, lesbian, gay, bisexual, transgender, queer, LGBTQ+, mental well-being, support, intervention, resilience, digital, co-design, sexual and gender minority youth, SGMY, mobile phone

## Abstract

**Background:**

Sexual and gender minority youth are at greater risk of compromised mental health than their heterosexual and cisgender peers. This is considered to be due to an increased burden of stigma, discrimination, or bullying resulting in a heightened experience of daily stress. Given the increasing digital accessibility and a strong preference for web-based support among sexual and gender minority youth, digital interventions are a key means to provide support to maintain their well-being.

**Objective:**

This paper aims to explicate the co-design processes and underpinning logic of *Oneself*, a bespoke web-based intervention for sexual and gender minority youth.

**Methods:**

This study followed a 6-stage process set out by Hagen et al (identify, define, position, concept, create, and use), incorporating a systematic scoping review of existing evidence, focus groups with 4 stakeholder groups (ie, sexual and gender minority youth, professionals who directly support them, parents, and UK public health service commissioners), a series of co-design workshops and web-based consultations with sexual and gender minority youth, the appointment of a digital development company, and young adult sexual and gender minority contributors to create content grounded in authentic experiences.

**Results:**

*Oneself* features a welcome and home page, including a free *accessible to all* animation explaining the importance of using appropriate pronouns and the opportunity to create a user account and log-in to access further free content. Creating an account provides an opportunity (for the user and the research team) to record engagement, assess users’ well-being, and track progress through the available content. There are three sections of content in *Oneself* focused on the priority topics identified through co-design: (1) coming out and doing so safely; (2) managing school, including homophobic, biphobic, or transphobic bullying or similar; and (3) dealing with parents and families, especially unsupportive family members, including parents or caregivers. *Oneself’s* content focuses on identifying these as topic areas and providing potential resources to assist sexual and gender minority youth in coping with these areas. For instance, *Oneself* drew on therapeutic concepts such as cognitive reframing, stress reduction, and problem-solving techniques. There is also a section containing relaxation exercises, a section with links to other recommended support and resources, and a *downloads* section with more detailed techniques and strategies for improving well-being.

**Conclusions:**

This study contributes to research by opening up the *black box* of intervention development. It shows how *Oneself* is underpinned by a logic that can support future development and evaluation and includes diverse co-designers. More interactive techniques to support well-being would be beneficial for further development. Additional content specific to a wider range of intersecting identities (such as care-experienced Asian sexual and gender minority youth from a minority faith background) would also be beneficial in future *Oneself* developments.

**International Registered Report Identifier (IRRID):**

RR2-10.2196/31036

## Introduction

### Background

Worldwide, it is estimated that up to 10% of the adolescent population identifies as being either a sexual or gender minority youth; that is, they identify as lesbian, gay, bisexual, transgender, queer, or another sexual or gender minority (lesbian, gay, bisexual, transgender, and queer [LGBTQ+]) [1-3]. Sexual and gender minority youth are known to be at greater risk of poor mental health than their heterosexual and cisgender peers [1,4]. This elevated risk is suggested to be largely related to an increased burden of stigma, discrimination, or bullying resulting in a heightened experience of stress in their day-to-day lives [5,6]. Clearly, work needs to continue to improve social environments for sexual and gender minority youth to reduce the additional stress they experience, but this will take time. In parallel, research is needed to identify what can be done to support sexual and gender minority youth to protect their mental health and well-being and help them build the skills and resilience they will need to thrive. This is increasingly important for the youngest sexual and gender minority youth as there is evidence suggesting that they are *coming out* at an earlier age than previous generations [6,7]. Their younger age may mean that they have had less time and opportunity to develop strong support networks and coping skills compared with those who come out at an older age [6,8].

Current and recent generations of young people have grown up in the digital age. Often referred to as *digital natives* [9], they have only experienced a world with access to the internet [9]. The latest data suggest that almost all homes in the United Kingdom have access to the internet [10] and 97% of individuals aged 12 to 15 years have their own mobile phone, with the vast majority using it to access the internet [11]. Young people are also known to spend much of their time in web-based spaces, which can assist their early attempts to seek information or obtain support on the issues they face. Similarly, a UK Department of Health and Social Care–commissioned report highlighted a strong preference among sexual and gender minority youth to access help on the internet, whereby 82.3% (n=572) of sexual and gender minority youth participants reported being “likely” or “very likely” to choose support in this format [12]. For this reason, providing web-based resources to support sexual and gender minority youth and the adults who assist them could be a widely accessible and relatively low-cost public health approach to improving their health and well-being.

In this paper, we present the detailed systematic steps we took to develop *Oneself,* a bespoke digital web-based resource to support sexual and gender minority youth regarding some of the most pressing challenges associated with growing up and being a sexual and gender minority young person. Drawing on the “identify, define, position, concept, create, and use” stages set out by Hagen et al [13] for participatory design with young people in mental health promotion, the process initially involved a scoping review of the strategies used in existing interventions [14]; in-depth interviews with adult experts who support sexual and gender minority youth, including parents; and focus groups with sexual and gender minority youth. We then engaged in a co-design process involving workshops with sexual and gender minority youth to determine priorities for the focus of the content and the look and feel of the resource and develop aspects of the content itself.

In addition to drawing on evidence from the scoping review, we drew on the firsthand expertise of sexual and gender minority youth as participatory research and co-design with intended end users of interventions are essential for their optimization in pragmatic terms. For example, knowledge about the needs of unique subpopulations may be limited, and co-design processes can help enhance an intervention’s acceptability [15-18]. In instances in which a group is frequently marginalized, such as sexual and gender minority youth, co-design is especially important because it represents a way to empower and democratize research and its outputs [19]. Co-design with underserved populations, including sexual and gender minority youth, allows pertinent diversity considerations to be addressed, for instance, factors regarding language, symbols, and character use in digital mental health technologies [20]. Hence, co-design processes are an attempt to help inform the creation of acceptable resources and assist in not only avoiding further alienating populations such as sexual and gender minority youth but also offering them a voice and greater inclusion. The approach applied by Hagen et al [13] was specifically chosen because it has been applied successfully in the past to support sexual and gender minority youth in terms of their mental health. Making intervention development processes replicable and transparent in how they are intended to bring about change for end users is also recognized as important for developing the science of health and well-being [21]. With this in mind, we outlined what we planned to do at the start of our project in our published study protocol [6]. This protocol was submitted in June 2021, before the project officially commenced.

### Aim

This paper sets out the systematic stages involved in developing *Oneself* for sexual and gender minority youth and describes how the findings or outcomes from each stage fed into content development and refinements. It also aims to clearly explicate how each feature and its content are intended to support sexual and gender minority youth and promote change so that any future research involving *Oneself* can incorporate evaluation against the logic that underpins it.

## Methods

### Overview

In accordance with our published protocol [6], we set out to follow the stages in intervention co-design as outlined by Hagen et al [13]. Intervention development and co-design are rarely a straightforward, linear process. In practice, some tasks need to happen in parallel, and researchers and coproducers may need to cycle back and repeat elements of the process as additional challenges emerge and new insights arise. The 6 stages of co-design involving adult experts and sexual and gender minority youth are set out below with a brief description of project activities involved in developing the resource aligned to that stage and links to the relevant methods section where more detail is provided (Textbox 1 [13]).

**Activities carried out across each of the 6 stages of co-design set out by Hagen et al to develop the web-based resource.**

**Identify**

- Focus groups with sexual and gender minority youth (see the *Interviews and Focus Groups With Sexual and Gender Minority Youth, Adult Experts, and Parents: Identify and Define Stages* section)
- Systematic scoping review (see the *Systematic Scoping Review: Identify and Define Stages* section)
- Interviews with adult experts and parents (see the *Interviews and Focus Groups With Sexual and Gender Minority Youth, Adult Experts, and Parents: Identify and Define Stages* section)

**Define**

- Focus groups with sexual and gender minority youth (see the *Interviews and Focus Groups With Sexual and Gender Minority Youth, Adult Experts, and Parents: Identify and Define Stages* section)
- Systematic scoping review (see the *Systematic Scoping Review: Identify and Define Stages* section)
- Interviews with adult experts and parents (see the *Interviews and Focus Groups With Sexual and Gender Minority Youth, Adult Experts, and Parents: Identify and Define Stages* section)
- Team co-development to finalize decisions and solutions (see the *Findings of the Research Team’s Co-Design Meetings in June 2022: Design, Position, and Concept Stages* section)

**Position**

- Initial co-design workshops with sexual and gender minority youth and email and web-based consultation (see the *Initial Co-Design Workshops With Sexual and Gender Minority Youth and Email or Web-Based Consultations: Position and Concept Stages* section)
- Appointment of digital developer (see the *Appointment of Digital Developer [Preparation for Delivering the Concept, Create, and Use Stages]* section)
- Team co-development to finalize decisions and solutions (see the *Research Team Co-Development to Finalize Decisions on the Focus and Topic Areas [Define, Position, and Concept Stages]* section)

**Concept**

- Initial co-design workshops with sexual and gender minority youth and email or web-based consultation (see the *Initial Co-Design Workshops With Sexual and Gender Minority Youth and Email or Web-Based Consultations: Position and Concept Stages* section)
- Questionnaire to assess *look and feel* design options (see the *Initial Co-Design Workshops With Sexual and Gender Minority Youth and Email or Web-Based Consultations: Position and Concept Stages* section)
- Team co-development to finalize decisions and solutions (see the *Findings of the Research Team’s Co-Design Meetings in June 2022: Design, Position, and Concept Stages* section)
- Appointment of sexual and gender minority community members through specialist media and modeling agencies (see the *Appointment of Sexual and Gender Minority Contributors Through Specialist Media and Modeling Agencies: Concept Stage* section)

**Create**

- Further co-design workshops with sexual and gender minority youth (see the *Further Co-Design Workshops With Sexual and Gender Minority Youth: Create Stage* section)
- Filming with sexual and gender minority contributors (see the *Introducing the Sexual and Gender Minority Contributors: Create Stage*section)
- Development work by appointed digital provider (see *The Oneself Resource* section)

**Use**

- Feedback from *think aloud* user interviews (MFG Lucassen, unpublished data, 2024)
- Feedback from adult expert interviews (MFG Lucassen, unpublished data, 2024)

### Ethical Considerations

Ethics approval for the aspects of the study involving human participation was granted by the Human Research Ethics Committee at The Open University (OU) before data collection began (ethics approval HREC/4059/Lucassen). All participants, both adults (eg, professionals who directly support sexual and gender minority youth) and adolescents, gave full informed consent to participate and signed a consent form to indicate this. Young people aged <16 years also required written parental consent to participate. Study data were anonymized before analysis, and all consent records were stored separately. Following the anonymization of interview and focus group transcripts, recordings and transcripts with person-identifiable information were deleted. Where applicable, participants were reimbursed for any transport costs associated with taking part and given a £20 (US $25.56) gift voucher per interview or focus group as a token of gratitude for their involvement.

### Systematic Scoping Review: Identify and Define Stages

The PRISMA-ScR (Preferred Reporting Items for Systematic Reviews and Meta-Analyses extension for Scoping Reviews) guidelines [22] were followed, and studies were included if they contained primary data on psychosocial coping strategies for sexual and gender minority youth, were conducted with adolescents (aged 10-19 years), and were published in English. The MEDLINE, Embase, and PsycINFO databases were searched. Search terms included a range of terms to capture a sexual and gender minority focus (eg, “gender minorit*” or “LGB*”) and a range of terms for psychosocial coping strategies (eg, “Coping*,” “adaptive,” and “resilience”). No date restrictions were applied, and the searches ran up to January 19, 2022. A descriptive approach to synthesizing the evidence, as recommended by Arksey and O’Malley [23], was used. The methods and findings of the scoping review have been published elsewhere [14]. The systematic scoping review ran in parallel to the focus groups with sexual and gender minority youth and interviews with adult experts and parents, which are reported in the following section.

### Interviews and Focus Groups With Sexual and Gender Minority Youth, Adult Experts, and Parents: Identify and Define Stages

A total of 6 focus groups, each with between 3 and 10 sexual and gender minority youth participants, were conducted between November 2021 and February 2022. To reach and recruit participants in the applicable age range from the target communities, we worked with 3 organizations supporting LGBTQ+ youth to advertise the opportunity. Focus groups were run in conjunction with these organizations, with their staff also attending to help young people feel comfortable and supported. Staff also assisted in the process of obtaining informed consent from sexual and gender minority youth and, for those aged <16 years, from their parents or guardians. Due to COVID-19 restrictions, all focus groups were hosted via videoconference. The sessions were audio recorded and transcribed. Once accurate transcripts were approved (by MFGL or ANG) and fully anonymized, the focus group electronic audio recordings were deleted. Participants were all secondary school-aged, primarily between the ages of 12 and 20 years, with those aged ≤15 years in a separate focus group. In total, 4 participants aged ≤25 years took part in the focus groups with the older participants because they had special educational needs (eg, learning disabilities) and, as such, were still engaged in secondary-level education or training. Table 1 provides demographic information about sexual and gender minority youth focus group participants; 81% (29/36) of the sexual and gender minority youth were gender minority youth (ie, their gender identity was not the same as their sex as recorded at birth). Many participants (14/36, 39%) were bisexual or pansexual. Approximately 1 in 5 sexual and gender minority youth (7/36, 19%) were of dual heritage (eg, European and West African) or from a migrant background (eg, the other White participants who were not White British).

In parallel, 16 one-to-one interviews were conducted with adult experts based in England, with 6% (1/16) of the participants in Wales, including parents of sexual and gender minority youth, between October 2021 and January 2022. A total of 25% (4/16) of the adults held posts as commissioners of public health services relevant to sexual health and well-being, roles that included consideration of the needs of sexual and gender minority youth. In total, 25% (4/16) of the experts worked in frontline practitioner roles supporting the health and well-being of young people, including sexual and gender minority youth (eg, clinicians working in child and adolescent mental health services). In total, 25% (4/16) of the experts were community-based professionals, such as sexual and gender minority youth workers and policing staff focused on reducing the mistreatment of sexual and gender minority individuals. A total of 25% (4/16) of the adults were parents of a sexual and gender minority adolescent interested in better supporting sexual and gender minority youth. As with the sexual and gender minority youth focus groups, interviews were conducted using videoconference software and audio recorded, and transcripts of the interviews were produced. Once anonymized and approved as accurate (by MFGL or ANG), the electronic audio recordings were deleted.

**Table 1:** Demographic information about sexual and gender minority youth involved in focus groups about factors affecting mental well-being between November 2021 and January 2022 in England (N=36)^a^.

Demographics			Values
Age of sexual and gender minority youth (y), mean (SD; range)			16.8 (2.6; 12-24)
**Gender of sexual and gender minority youth^b^, n (%)**			
	Nonbinary	10 (28)	
	Male	5 (14)	
	Female	5 (14)	
	Transgender man or male	5 (14)	
	Questioning	2 (6)	
	Other responses (eg, gender fluid)	9 (25)	
Is your gender identity the same as your sex recorded at birth?—no, n (%)			29 (81)
**Sexuality of sexual and gender minority youth, n (%)**			
	Bisexual	9 (25)	
	Pansexual	5 (14)	
	Gay	5 (14)	
	Queer	2 (6)	
	Questioning	2 (6)	
	Lesbian	1 (3)	
	Not heterosexual	1 (3)	
	“Unlabeled”	1 (3)	
	“N/A”^c^	1 (3)	
	2 responses (eg, bisexual and queer)	4 (11)	
	≥3 responses (eg, bisexual, pansexual, and not heterosexual)	5 (14)	
**Ethnicity of sexual and gender minority youth, n (%)**			
	British	3 (8)	
	British and North African	1 (3)	
	European and West African	1 (3)	
	Mixed	1 (3)	
	Other White	3 (8)	
	European	1 (3)	
	White British	25 (69)	
	Not stated	1 (3)	
Felt down or low for more than a few days in a row?—yes, n (%)			31 (86)

^a^6 focus groups in total with between 5 and 11 participants each; 44 participants in total (including 8 youth workers).

^b^This item was an open-ended question; as such, 3 gender minority youths wrote *Male* or *Female* (ie, *Male* and *Female* here does not necessarily equate to being cisgender and male or female).

^c^N/A: not applicable.

### Appointment of Digital Developer (Preparation for Delivering the Concept, Create, and Use Stages)

In January 2022, a tender specification for a digital developer was created based on the outcomes at that time from the identify, define, and position work outlined previously. A range of commercial developers were notified of the tender, and after a competitive process involving an assessment of providers’ submissions and web-based interviews, Bluestep Solutions Limited (Bluestep for brevity) were appointed. They supported the research team in the task of translating the findings that emerged from the preceding evidence-gathering stages (ie, the scoping review, interviews, and focus groups) to content for the digital resource. Bluestep’s expertise resided in developing engaging and user-friendly content aligned with the research team’s evidence-informed approach. Co-design workshops with sexual and gender minority youth participants were conducted to refine the pilot content and improve its look and feel (described in the *Findings of the Co-Design Workshops With Sexual and Gender Minority Youth: Position and Concept Stages* section). Bluestep provided a map of the potential structure and parameters of the digital resource that could be developed within the available budget. The original budget was £41,000 (approximately US $50,000). Some savings were made in the project’s overall budget, and additional funds were also sourced through the OU, resulting in a final budget of nearly £50,000 (approximately US $61,000). To remain within budget, Bluestep indicated that the research team should focus on 3 core sections of content and have only 1 full day of filming.

### Initial Co-Design Workshops With Sexual and Gender Minority Youth and Email or Web-Based Consultations: Position and Concept Stages

Two initial co-design workshops were held with (1) older sexual and gender minority youth aged ≥16 years (May 2022) and (2) younger sexual and gender minority youth aged 12 to 15 years (June 2022). The main aim of these workshops was to identify the priority issues and challenges faced by sexual and gender minority youth on which to focus and the preferred solutions and strategies that should be highlighted. To achieve this, 10 possible topics or issues and 11 possible solutions or strategies were presented to them based on data from the scoping review and the earlier interviews with adults and focus groups with sexual and gender minority youth. A modified nominal group technique [24] was used to facilitate this process. This involved structured voting before group discussions on the possible topics for inclusion, where all attendees were given an opportunity to express their views and preferences.

In June 2022, Bluestep created a selection of visual concepts (Multimedia Appendix 1) with different color palettes and visual *tones of voice* represented by imagery. For example, the *inclusive* visualized toolkit included a bright rainbow color palette, and the message toning was intended to represent inclusivity and messaging that “we’re all in it together.” The overall concepts were also set out alongside some suggested names (created from a marketing perspective) from Bluestep for the digital resource. The suggested names, which drew on commercial marketing expertise from Bluestep, included the following:

1. MEE: Mindful Education & Enlightenment for LGBTQ+
2. Oneself: Defined by you, allied by us
3. Free to be: Mindful tools for your journey

These visual concepts and suggested names were shared with our sexual and gender minority youth workshop participants, and they gave their feedback with support from youth workers via email and in a web-based consultation session via videoconference. The ultimate decisions about concepts, color schemes, and names were strongly informed by the sexual and gender minority youth’s views and we were led by their preferences. A set of questions to prompt discussions regarding preferences was provided to the youth workers supporting the consultation process.

### Research Team Co-Development to Finalize Decisions on the Focus and Topic Areas (Define, Position, and Concept Stages)

Following the second sexual and gender minority youth co-design workshop, the research team met to reflect on the voting decisions of sexual and gender minority youth and discuss their own ideas for the priority content and sections in the resource and its features (eg, video clips and animations). In addition to professional expertise, members of the team also have lived experience from their personal lives on which to draw (eg, MFGL is a White migrant, queer male individual and gender role nonconformer; RS is from an ethnic minority group and has lived experience of mental illness [25]; and KB is White British, grew up with a sibling who identifies as a gay cisgender male, and has lived experience of mental illness). The team held 2 meetings 1 week apart in June 2022.

### Questionnaire to Assess the Look and Feel of the Design Options: Concept Stage

Parallel to the co-design and development work outlined previously, Bluestep produced a number of design concepts for consideration by our sexual and gender minority youth workshop attendees and the research team (Multimedia Appendix 1). A questionnaire was developed that asked sexual and gender minority youth workshop attendees to consider the designs and some other key features related to the look and feel of the resource, such as whether the *characters* featured should be real people or fully animated or whether the *characters* should be acting out scenarios versus sharing their own personal experiences as sexual and gender minority individuals (Multimedia Appendix 2).

### Appointment of Sexual and Gender Minority Contributors Through Specialist Media and Modeling Agencies: Concept Stage

On the basis of our understanding of the need for credible sources to deliver messages in our intervention, and because the dramatizations we had initially envisaged for *Oneself* in our original study protocol were deemed too contrived and artificial by sexual and gender minority youth, we made a notable decision. In particular, it was identified that real sexual and gender minority young adults, who can talk authentically about their own experiences growing up as sexual and gender minority individuals, would be an important feature of *Oneself*. In July 2022, the process of recruiting 3 sexual and gender minority young adult contributors or community members was initiated. We applied to modeling and talent agencies given that we wanted contributors who were comfortable in front of cameras. We were provided with a dozen portfolios of different potential sexual and gender minority contributors and short introductory video clips on why they were interested in being involved in the development of *Oneself*. The research team and sexual and gender minority youth considered the clips separately, and the sexual and gender minority youth voted on their preferred contributors or community members. Feedback on the initial possible contributors highlighted that there was a lack of diversity, particularly regarding ethnicity and body size (ie, they looked “too much like models”). In our attempts to ensure a broader representation, we went back a second time to the agencies to obtain further potential contributor options.

### Further Co-Design Workshops With Sexual and Gender Minority Youth: Create Stage

In total, 2 additional co-design workshops were held in September 2022 and January 2023. Co-design workshops were hosted in person with MFGL, Bluestep, or ANG present. Audio recordings were transcribed, and once accurate transcripts were approved (by MFGL or ANG) and fully anonymized, the co-design workshop audio recordings were deleted. Participants were all secondary school-aged, primarily between the ages of 12 and 20 years, with those aged ≤15 years in a separate workshop. Demographic information about workshop participants is presented in Table 2. A total of 93% (14/15) of the participants were gender minority youth (ie, their gender identity was not the same as their sex as recorded at birth), and 60% (9/15) were bisexual or pansexual. Approximately one-quarter of sexual and gender minority youth (4/15, 27%) were of a dual heritage (eg, Asian and Black) or from a migrant background (eg, White participants who were not White British). An in-person consultation also bridged co-design workshops 3 and 4. This was not recorded.

**Table 2:** Demographic information about sexual and gender minority youth interested in supporting the mental well-being of sexual and gender minority youth involved in Oneself co-design workshops between May 2022 and January 2023 in England (N=15)^a^.

Demographics		Values
Age of sexual and gender minority youth (y), mean (SD; range)		15.7 (2.4; 12-20)
**Gender of sexual and gender minority youth, n (%)**		
	Nonbinary	5 (33)
	Gender fluid	3 (20)
	Other responses (eg, agender and demigirl)	2 (13)
	Transgender man or male	2 (13)
	Male or boy	2 (13)
	“No idea”	1 (7)
Is your gender identity the same as your sex recorded at birth?—no, n (%)		14 (93)
**Sexuality of sexual and gender minority youth, n (%)**		
	Bisexual	6 (40)
	Pansexual	3 (20)
	Questioning	1 (7)
	Omnisexual	1 (7)
	Not heterosexual	1 (7)
	Abrosexual	1 (7)
	“Don’t know”	1 (7)
	Left blank or no response	1 (7)
**Ethnicity of sexual and gender minority youth, n (%)**		
	British	1 (7)
	Mixed (eg, Asian and Black)	2 (13)
	White	2 (13)
	White British	10 (67)
Felt down or low for more than a few days in a row?—yes, n (%)		13 (87)

^a^4 co-design workshops in total (with between 5 and 8 youth participants each); 19 participants in total (including 4 youth workers).

## Results

### Results of the Systematic Scoping Review: Identify and Define Stages

The findings of the scoping review have been published previously [14]; however, a summary is presented in this section of what we learned that fed into our thinking about the content for *Oneself*. A total of 68 articles were identified as meeting the review criteria. The oldest paper dated from 2008, and more than half (25/68, 51%) were published from 2017 onward. Most studies (40/68, 59%) were small scale (ie, with <50 participants), and more than two-thirds (47/68, 69%) were conducted in the United States. In total, 26 studies included sexual minority youth only, a further 28 included sexual and gender minority young people, and 14 studies included only gender minority young people.

A total of 24 of the included articles focused on 17 unique interventions to support sexual and gender minority youth. More than half of the intervention papers (13/24, 54% studies) focused on both sexual and gender minority youth. In total, 9 studies included only sexual minority young people, and 2 studies focused on gender minority youth only. Of the 17 interventions, the most frequently cited therapeutic modality was cognitive behavioral therapy (11/24, 46% studies and 6/17, 35% interventions). Common features described in these interventions, including those with CBT-based modalities, are summarized in Table 3 [14].

Most of the interventions involved in-person delivery (14/24, 58% studies). In total, 5 (56%) out of 9 interventions were delivered in a digital format. In addition to the strategies and techniques outlined in Table 3, it was also noted that interventions often sought to affirm sexual and gender minority youth identities and give a message of hope to intervention users (eg, “I won’t always feel this way” in the Rainbow SPARX intervention [26]).

A total of 44 of the included studies did not focus on interventions per se. Instead, they were mainly qualitative studies (with some mixed methods studies combining survey and qualitative data) that explored the experiences of sexual and gender minority youth and the strategies they used to cope with the challenges they face. Table 4 [14] summarizes the commonly identified strategies and tools for sexual and gender minority youth drawn from these studies and applied to *Oneself*.

Taken together, the strategies listed in Tables 3 and 4 gave us a comprehensive list of potential contenders to make up the core content and features of *Oneself*. We drew on this information and the findings we present in the following section from our focus groups and interviews to develop the content of the 3 topic areas identified as most important.

**Table 3:** Common techniques identified internationally from existing interventions (ranked by frequency) to support co-design processes in the current project for sexual and gender minority youth and their mental well-being from a systematic scoping review published elsewhere in 2022.

Technique or coping strategy	Interventions that included it, n	Explanation of technique	How this was applied in *Oneself*
Peer support for sexual and gender minority youth	7	Providing a format or space for peer-to-peer support	6 relevant “Additional Resources” (all web-based) provided (eg, “LGBT Switchboard”)
Cognitive restructuring	6	For example, use of the ABCD technique—involves considering an activating event and the negative belief or thought that may accompany that, considering the consequence of that thought or belief, and then actively disputing any negative beliefs or thoughts to restructure the thinking around them	ABCD downloadable resource (entitled “Rejecting the Negativity”) included in the “Dealing with School” section
Problem-solving	5	Providing a structured way of thinking about problems and possible solutions	Problem-solving using “STEPS” included in the “Dealing with Parents and Families” section and firsthand experiences of problems and solutions provided by sexual and gender minority contributors
Behavioral activity or activation	4	Promoting activity that improves mental well-being because the activity is pleasant to do (eg, dancing and singing)	Provided as self-care tips under “Additional Resources” in the “Chilling Out” section
Recognizing problematic cognitions	4	Support to recognize own negative thinking patterns in response to the environment or others’ actions	ABCD downloadable resource (entitled “Rejecting the Negativity”) included in the “Dealing with School” section
Relaxation exercises	3	These include activities such as breathing exercises and progressive muscle relaxation techniques	3 relaxation exercises included in the “Chilling Out” section
Enhancing social or environmental support	3	Supporting the person to take positive action toward building a supportive social network	“Finding Allies” activities under the “Dealing with School” section
Psychoeducation	3	Education about the link between environmental stressors and well-being and between own thinking and behavior on emotional well-being	Quotes from sexual and gender minority youth used across Oneself and firsthand accounts from sexual and gender minority contributors (audiovisual content)
Building family relationships	2	Supporting family members in improving communication skills to enhance relationships	“Standing Up for Yourself”—exercise on how to best get one’s point across under the “Dealing with Parents and Families” section
Educating families	2	Educating family members to help them improve their attitudes and behaviors with their sexual and gender minority youth children or siblings	Quotes from sexual and gender minority youth (eg, “Know that it’s a lack of understanding rather than a lack of love”)
Raising awareness of resources	2	Identifying other sources of information and support that are available	6 relevant “Additional Resources” (all web-based) provided (eg, “LGBT Switchboard”)
Public narratives	2	Sharing of stories, such as “coming out” stories to help promote public discourse	The “Coming Out” section includes firsthand accounts from sexual and gender minority contributors (audiovisual content)

**Table 4:** Common strategies identified internationally to support co-design processes in the current project for sexual and gender minority youth mental well-being (ranked by frequency) from a systematic scoping review published elsewhere in 2022.

Strategy or tool	Studies that identified it, n	Explanation
The internet is an important tool to achieve connection with other sexual and gender minority youth	8	Internet seen as “lifesaving”
Social support and connection with other sexual and gender minority youth	6	Need to meet people “like me”
Taking on a peer educator or political advocacy role	5	Taking on an empowering role such as that of an educator or advocate was seen as valuable
Mentoring or providing support to other sexual and gender minority youth	4	Mentoring was also seen as useful and had the ability to build sexual and gender minority youth social networks
Escaping challenging environments and creating “pockets of safety”	4	Sexual and gender minority youth could not easily leave unsupportive environments but could potentially enhance their safety
Cognitive strategies to manage negative messages (such as the cognitive restructuring exercise or ABCD in Table 3)	4	CBT^a^ techniques designed to enhance the mental well-being of sexual and gender minority youth
Choosing when to be “out” vs “learning to hide” as adaptive strategies to manage well-being	4	Not coming out can be necessary to stay safe, but choosing to come out to the right people increases access to social support
Self-harming (not considered)	4	Although not recommended as a coping strategy, it was discussed in this way by some sexual and gender minority youth
Distraction techniques to take mind away from worries or improve mental well-being	3	This included using social media, gaming, watching web-based media, and exercising outdoors
Other risky coping strategies (eg, suicide attempts, risky sex, drug taking, and alcohol consumption; not considered)	3	Although not recommended coping strategies, they were discussed in this way by some sexual and gender minority youth
Use of mindfulness, emotional regulation strategies, cognitive reappraisals, assertive communication techniques, and resistance of rigid cultural boundaries	3	Strategies that are frequently part of CBT-based interventions
The internet can be a problematic space and can lead to exposure to mistreatment—sexual and gender minority youth need to be skilled users to protect themselves	3	Sexual and gender minority youth can be excluded or subject to abuse in web-based contexts, so managing safety with privacy settings or blocking abusers is necessary
Avoidance strategies—physically and emotionally	2	Attempting to suppress certain emotions at times or physically walking away

^a^CBT: cognitive behavioral therapy.

### Results of Interviews and Focus Groups With Sexual and Gender Minority Youth, Adult Experts, and Parents: Identify and Define Stages

To expedite drawing out the relevant data from the focus group and interview transcripts and inform *Oneself’s* development, the data were divided between the research team and examined carefully. Detailed notes were made regarding the sorts of issues that the various stakeholders identified as important to address. Details on strategies and tools that were deemed useful in participants’ experiences were also extracted. The issues and strategies identified were revised during 2 team meetings in June 2022. A more detailed framework analysis [27] of the data is underway and will be published in due course.

The rapid data extraction process provided us with a series of initial issues and potential areas or populations of focus. MFGL and ANG then met to construct a long list of the main issues (n=10) and the potential solutions or strategies (n=11) that emerged from the findings of the scoping review and the interviews and focus groups with stakeholders. These are summarized in Textboxes 2 and 3.

**The main topic areas identified as a priority for sexual and gender minority youth mental well-being during co-design in England (2022).**

- How to deal with unsupportive parents or other family members
- How to deal with bullying at school (eg, name calling)
- How to deal with the challenges associated with coming out
- How to deal with negativity directed at lesbian, gay, bisexual, transgender, and queer people (eg, from a religion)
- How to deal with misgendering
- How to deal with feeling isolated or alone
- How to deal with stigma (eg, homo-, bi-, or transphobia)
- How to deal with web-based abuse (eg, trolls saying nasty things)
- How to explore and make sense of your sexuality or gender
- How to deal with people not believing you about your sexuality or gender

**The potential toolkit focus areas or populations identified as a priority for sexual and gender minority youth mental well-being during co-design in England (2022).**

- Educate teachers and others on how to better support lesbian, gay, bisexual, transgender, and queer (LGBTQ+) youth so that school environments can be improved for LGBTQ+ youth
- Educate parents (and other people in the community) on how to better support LGBTQ+ youth so that communities can be improved for LGBTQ+ youth
- Help young people with practical issues—in particular, finding a toilet that they can safely use
- Allow the young person to connect directly with other LGBTQ+ young people so that they can talk to someone else who understands
- Coming out and how to do this safely—highlight that it is OK not to come out (and it is also OK to change one’s mind)
- Up-to-date and accurate information on sexuality and gender to help them make sense of their identity
- How to find supportive people via web-based environments so that they have a better support network
- Help young people figure out what they can and cannot change themselves so that they know what to focus their energy on
- Use affirmations (positive messages) about the young person (eg, “I deserve kindness” and “my gender is not an inconvenience”) to help people feel even better about themselves
- Help young people engage in creative activities (eg, art and music) to make them feel better
- Provide the contact phone numbers and details for supports available to LGBTQ+ youth so that they know where to go for extra help

### First Proposed Structure and Designs After Appointing the Digital Developer: In Preparation for the Concept, Create, and Use Stages

Bluestep provided a map of the potential structure and parameters of the digital resource that it would be possible to develop within the available budget, specifically a wireframe. A copy of the structure is provided in Multimedia Appendix 3. This illustrates the inclusion of 3 core features or sections of content and a “free” (all content is free to access, but the main content requires the user to create an account with a username or email address and password) taster section of content proposed as important to engage potential users and educate the wider public (eg, teachers).

### Findings of the Co-Design Workshops With Sexual and Gender Minority Youth: Position and Concept Stages

Table 5 shows the average rank order preferences from the adapted nominal group technique voting in relation to priority issues or topics to cover within the *Oneself* resource. Participants ranked their highest-priority topic as rank 1 and their lowest-priority topic as rank 10. The lowest average rank order identifies the highest preference among the group. Dealing with unsupportive parents or other family members and dealing with bullying were the highest-ranked topics to cover. Table 6 presents the average rank order preferences for possible solutions or strategies to include in *Oneself*. The highest-ranking content included educating parents and teachers to help improve the quality of the environments they live in.

**Table 5:** Average sexual and gender minority youth rank order preferences of topics to cover in relation to sexual and gender minority youth mental well-being for co-design purposes in England (June 2022)—ordered from highest to lowest priority.

Topics	Score, mean (SD; 1=highest priority to 10=lowest priority)
Unsupportive parents or family	3.4 (2.3)
Dealing with bullying	3.8 (3.2)
Coming out	4.3 (2.2)
Negativity due to differing beliefs	4.5 (2.1)
Misgendering	4.8 (2.8)
Feeling isolated or alone	5.8 (3.1)
Dealing with stigma and homo- or transphobia	5.9 (2.1)
Dealing with online abuse	6.5 (3.1)
Making sense of sexuality or gender	7.4 (3.0)
Dealing with not being believed	7.5 (2.1)

**Table 6:** Average sexual and gender minority youth rank order preferences of potential solutions or strategies in relation to sexual and gender minority youth mental well-being for co-design purposes in England (June 2022)—ordered from highest to lowest priority.

Topics	Score, mean (SD; 1=highest priority to 11=lowest priority)
Educate teachers to improve school environments	3.4 (2.3)
Educate parents and others to improve community environments	3.5 (2.9)
Contact details for support	4.2 (2.9)
Practical issues (eg, finding gender-neutral toilets)	4.9 (3.1)
Connecting with other LGBTQ+^a^ young people	5.4 (2.7)
Advice on coming out safely	5.5 (2.6)
Support to make sense of gender or sexuality	5.9 (2.7)
Help to find supportive people online to build networks	6.4 (1.7)
Knowing what to spend their energy on	7.4 (2.9)
Affirmations to make sexual and gender minority youth feel better about themselves	8.2 (2.7)
Support to engage in creative activities to enhance wellbeing.	8.5 (3.0)

^a^LGBTQ+: lesbian, gay, bisexual, transgender, and queer.

### Findings of the Research Team’s Co-Design Meetings in June 2022: Design, Position, and Concept Stages

The first co-design meeting with the research team began by reflecting on the rank order preferences of the sexual and gender minority youth (presented previously). It was acknowledged that, although clear priorities emerged from the data, there was also considerable variability in the rank order preferences. With the budget and practical limits to the amount of content that we could include, we could not create an ideal resource to suit all sexual and gender minority youth needs. However, given the identification by Bluestep that we could have three main sections with featured content, the selection of the top three topic areas was straightforward: (1) coming out and doing so safely; (2) managing school, including homophobic, biphobic, or transphobic bullying or similar; and (3) dealing with parents and families, especially unsupportive family members, including parents or caregivers. We found the favored focus area or population being about educating parents, teachers, and other community members to be outside the scope given our budget to date and as the resource was always intended to be primarily for sexual and gender minority youth themselves rather than adults who support them. The resource is designed to center the experiences of sexual and gender minority youth, but we expect that *Oneself* will ultimately support parents, teachers, and other community members by increasing awareness and visibility of sexual and gender minority youth experiences. We do acknowledge that there are important challenges in balancing (individual-focused) support for sexual and gender minority youth with promoting social justice through education of adult stakeholders. As was done in this study, it is important to consider these elements in parallel because they are interactive. While we decided not to explicitly target adults at this stage, we acknowledged this request as being part of sexual and gender minority youth’s desire for the environments they live in to be better and more supportive of them, hence the decision to prioritize the educational animation about pronouns, intended for a wider audience (including parents and teachers). We also reflected on the fact that, while the main purpose of the content should be to help young people cope with situations independently, it could also be useful for educating parents, teachers, and other members of the community. Specifically, the resource could help them understand the unique challenges of growing up as a sexual and gender minority youth and how they can act and respond supportively to promote positive social change. At this stage, we thought that the formats we might use to present content could be videos or animations depicting narratives of sexual and gender minority youth everyday experiences, possibly with some interactive content or features for the user.

Sexual and gender minority youth understandably had a range of perspectives and ideas about what should be covered in *Oneself*. We identified 9 such specific suggestions. For instance, we were cautioned against educating *Oneself* users on the various sexuality and gender “labels” used by a young person given that the terminology is continually evolving (and frequently contested). Another sexual and gender minority youth felt strongly that we should acknowledge the difficulties associated with challenging environments; for example, “you cannot change everyone,” and therefore, a sexual and gender minority youth must know how (and when) to “walk away.” They also wanted us to ensure that our sexual and gender minority contributors would represent as much diversity as possible. By the end of the research team discussions, there was a growing sense that we could cover, to some degree, many of the preferred solutions or strategies that had been discussed and voted on by sexual and gender minority youth in their co-design workshops, with a focus on the top 3 topics or issues.

It was beyond the scope and resources of *Oneself* to provide a web-based community space where sexual and gender minority youth could connect with each other safely in real time as this would likely require constant monitoring and ongoing administration. However, advice on where or how to do this elsewhere could be included, along with links to other supportive resources. It was decided that the resource would focus on supporting sexual and gender minority youth directly. We aimed to center the young person in this resource, with *Oneself* often talking directly to them and trying to focus on them and their needs, for instance, by using language or terms and concepts that map to the concerns they have raised with us as the research team. This act of centering is in direct contrast to the marginalization that they may face daily. It was also intended to have a dual purpose of potentially serving to educate the wider community, including parents and teachers. It was felt that, because the 3 main topics focused on dealing with challenges that can have a detrimental effect on well-being, the resource needed to include evidence-based tools and resources known to support and enhance mental well-being, such as relaxation techniques and other relevant means of coping. It also needed to include content that felt empowering of developing and evolving identities to support and develop users’ self-esteem.

### Findings of the Questionnaire to Assess the “Look and Feel” Design Options: Concept Stage

The wireframe structure of *Oneself* (which was designed to include some introductory content) was confirmed first. This included a log-in feature to access the 3 main content sections and recommended additional resources and sources of help and support. The log-in feature, with the associated gathering of demographic data, was deemed necessary to capture future user information related to *Oneself.* Next, Bluestep worked with the research team to develop a questionnaire posing different design concepts and options for the look and feel of the resource. The full questionnaire and the options posed are presented in Multimedia Appendix 2. The preferences that this process helped identify are briefly summarized in the following paragraph.

Although the idea for having full animations with voice actors was rated favorably by many sexual and gender minority youth participants, a clear overall preference emerged for using real people talking about their firsthand experiences growing up as sexual and gender minority youth, as well as the inclusion of sexual and gender minority youth “influencers” or public figures. There were also clear indications that the resource would most likely be accessed on a smartphone by sexual and gender minority youth and that video clips should include audio subtitles (to enable viewing without sound on; however, this is also valuable for accessibility reasons), and most indicated that they would use headphones to listen to content, too. On the basis of sexual and gender minority youth feedback, video-based content should ideally not exceed 60 seconds; some were willing to watch longer clips when the content was engaging. Downloadable information sheets, for access again offline, were identified as useful, and sexual and gender minority youth participants favored a color palette that was pastel and informed by the “progress rainbow flag.”

### Team Consultation Based on the Questionnaire Feedback Led to Plans for Inclusion of Sexual and Gender Minority Contributors: Concept and Create Stages

The feedback we obtained about the inclusion of sexual and gender minority contributors (ie, not actors playing a role) led to further consultation about the format of the resource and a decision to focus the main content on testimonial or account footage from sexual and gender minority young adults who could reflect on their experiences with the topics selected when they were growing up. We set out to identify individuals from modeling and talent agencies who would be willing to provide this kind of content, as described in the *Appointment of Sexual and Gender Minority Contributors Through Specialist Media and Modeling Agencies: Concept Stage* section.

The process of assessing potential sexual and gender minority content contributors resulted in the appointment of 3 people who identified as sexual and gender minority individuals who were willing to be involved for a set fee. Between them they represented diversity in terms of gender and sexual identity, body shape and size, ability, and ethnicity. More details about those selected are provided in the following section.

### Design Concept Selection via Email and Web-Based Consultation With Sexual and Gender Minority Youth and Outcomes From Co-Design Workshops 3 and 4: Position, Concept, and Create Stages

**Figure 1:**
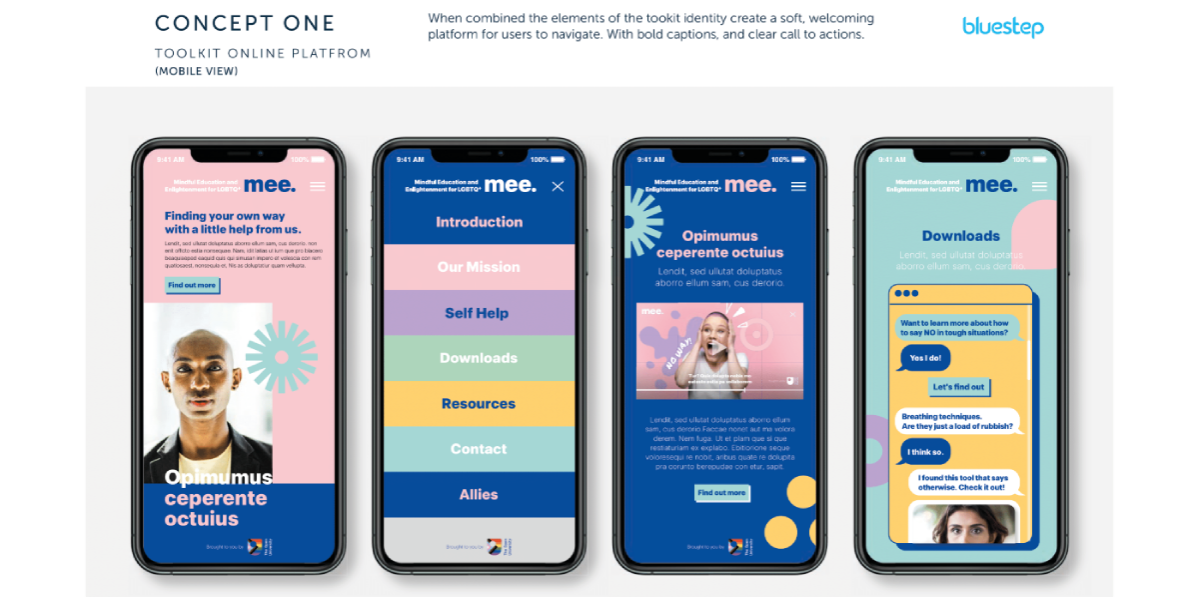
Concept 1 reviewed by sexual and gender minority youth interested in supporting sexual and gender minority youth mental well-being during co-design processes in England (2022).

**Figure 2:**
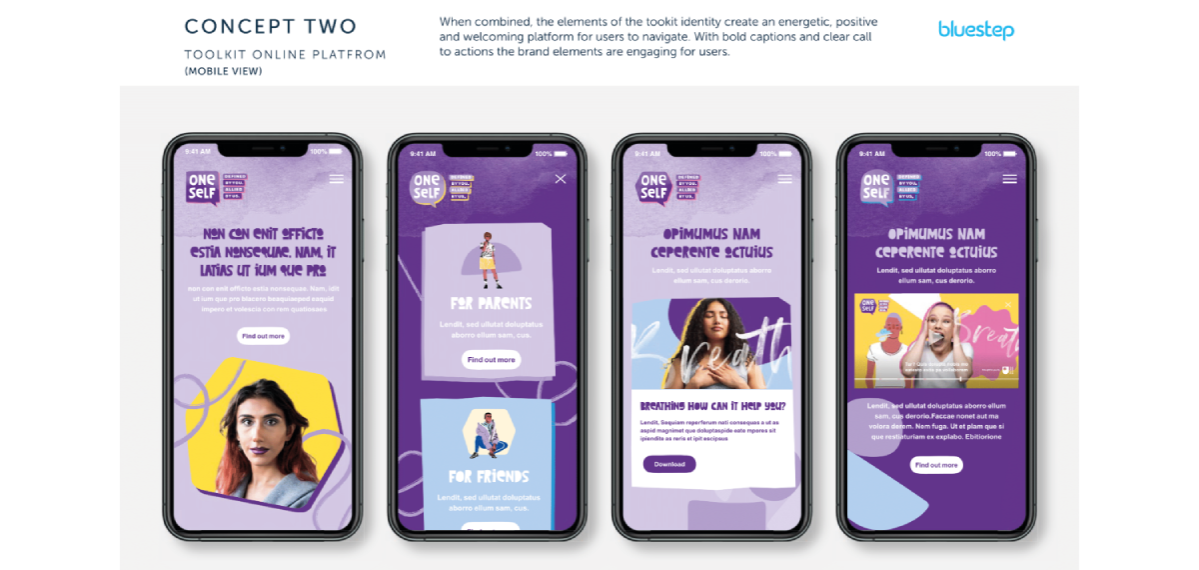
Concept 2 reviewed by sexual and gender minority youth interested in supporting sexual and gender minority youth mental well-being during co-design processes in England (2022).

Concept 1 (Figure 1) was a clear favorite in terms of the color scheme, and it was described as more “friendly” and inclusive than concept 2 (Figure 2). There was a question regarding the icons in both concepts (ie, symbols transposed over certain images); sexual and gender minority youth did not feel that the icons represented the topics adequately, and therefore, wording or text would be needed, which would defeat the purpose of using icons. In concept 1, a “share” function was seen as more understandable as it was interpreted as a speech bubble, though this could be made even clearer.

From concept 2, sexual and gender minority youth liked the “squiggly lines” in the designs if they could be incorporated into concept 1’s color scheme. It was preferred that design elements from both concepts could be used in the final resource, although sexual and gender minority youth were clear not at the same time as it would be too much on one image.

The sexual and gender minority youth participants were asked if they thought that including the OU (lead university for the project) logo on the resource was a good idea. Most participants felt that it would give people confidence in the quality of the resource as OU is a well-known brand in the United Kingdom. The preferred name for the resource, of the 3 suggestions, was *Oneself*, but they considered the inclusion of the originally proposed taglines to be too long. Consequently, we did not use a subsequent lengthy tagline in combination with the name *Oneself* across the whole resource.

Table 7 provides a summary of the workshops and consultations by date, including what was covered and how it aligns with the co-design stages by Hagen et al [13].

**Table 7:** A summary of the co-design workshops and consultation sessions in 2022 and 2023 in England that led to the creation of Oneself—a tool to support the mental well-being of sexual and gender minority youth.

Phase	Timeline	Strategies or features chosen	Stage
Co-design workshops 1 and 2 with sexual and gender minority youth	May 2022 to June 2022	Live video and animation combination (stylistic) Use of self-identified sexual and gender minority contributors or influencers (format) Downloadable information sheets (format) Subtitles for all video clips (format)	Position Concept
Research team co-design meetings	June 2022	Sexual and gender minority youth decide on the look and feel (stylistic) Prototype toolkit structured around 3 key features: dealing with parents and families, dealing with school, and coming out (content) Contributors or community members instead of actors and dramatizations (format)	Position Concept Create
IT development	June 2022 to December 2022	First draft of look and feel (stylistic) Wireframes (structure)	Position Concept Create
Email consultations with sexual and gender minority youth	July 2022 to September 2022	Style and color scheme confirmed (stylistic) Title and name (stylistic) Use of the OU^a^ logo confirmed (stylistic)	Create
Co-design workshop 3	September 2022	“Pearls of wisdom” and quotes from sexual and gender minority youth (content) Choosing the contributors: demo reels (content) Choosing a brief tagline (to be used infrequently): “supportive tools for your journey” (stylistic)	Create
In-person follow-up consultation session	October 2022	Feedback on refining the colors, images, and design (stylistic), including initial drafting and then critiques of the pronoun animation script (content)	Create
Co-design workshop 4	January 2023	Pronoun animation storyboard approval (stylistic) Choosing key points and information from filmed material and initial contributor clips (content)	Create

^a^OU: The Open University.

### Introducing the Sexual and Gender Minority Contributors: Create Stage

Bluestep shortlisted 10 candidate sexual and gender minority young adult contributors for the research team, who in turn shortlisted 5 to present to the young people in co-design workshop 3. There were some unforeseen recruitment difficulties. For example, the selected racial and ethnic minority gay man and a transgender woman (who was one of the sexual and gender minority youth’s top choices) were unfortunately not able to participate as initially agreed. For instance, one of them became concerned about how publicly accessible *Oneself* would be once released (ie, they could be “outed” to a whole range of people known to them). Thus, 2 female contributors were selected from the initial shortlist, and both were rated very favorably by the sexual and gender minority youth. As it was important for the project to reflect diversity across gender identity, sexuality, race, and disability, a further search for a third contributor was carried out in October 2022. Finally, 3 contributors were selected and approved by the young people: Chloe, Lilly, and Georgie.

Georgie, also known as Triple Minor, uses they, she, or he pronouns and is transgender nonbinary. Georgie wanted to contribute to *Oneself* because they were keen to be the much needed representation that is often lacking within LGBTQ+ communities.

Lilly uses she or her pronouns and is pansexual. Lilly wanted to contribute to *Oneself* because, when she was younger, she would have loved to have heard more about queer perspectives. This is why she wanted to talk about her own experiences.

Chloe uses she or her pronouns and is a lesbian. Chloe wanted to contribute to *Oneself* because she believes it is important for the younger LGBTQ+ community to feel supported and comfortable in their sexuality and be able to hear the voices and perspectives of queer people.

**Figure 3:**
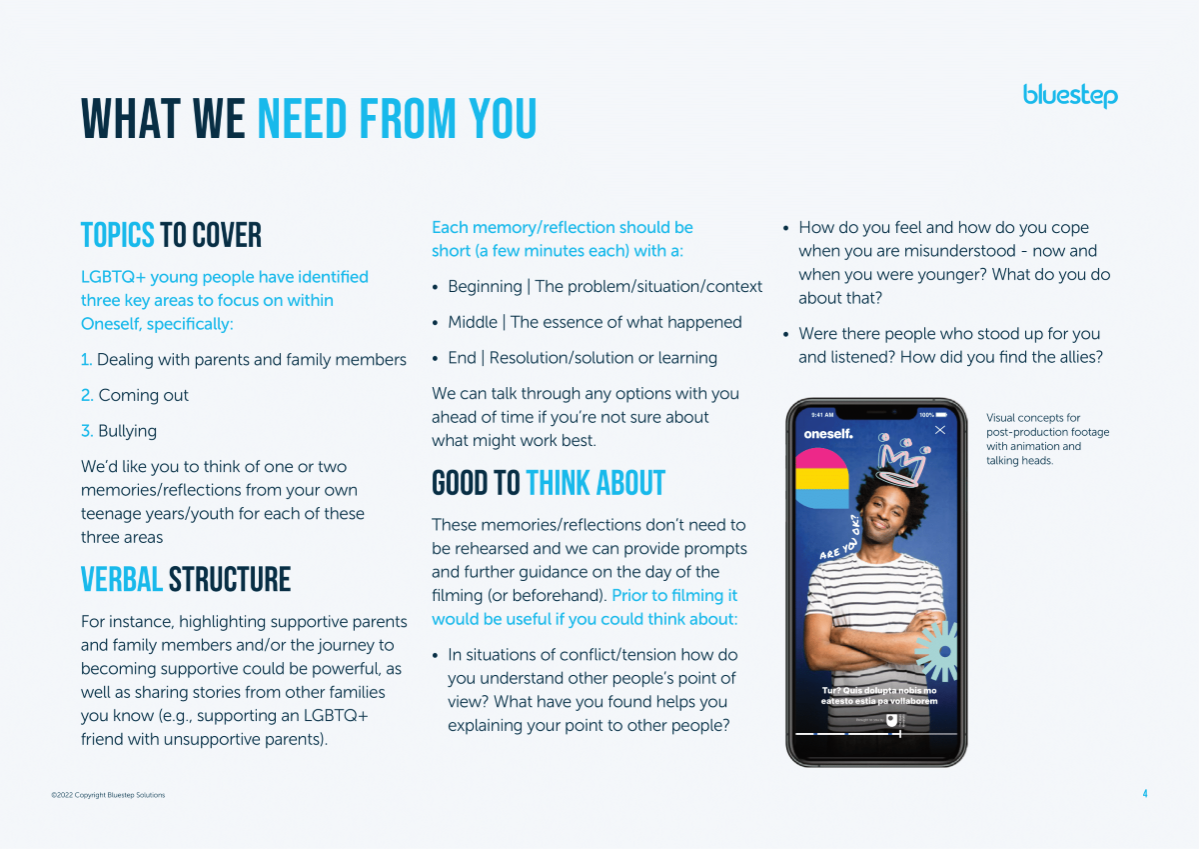
Creative briefing reviewed by sexual and gender minority contributors interested in supporting sexual and gender minority youth mental well-being during co-design processes in England (2022; part 1). LGBTQ+: lesbian, gay, bisexual, transgender, and queer.

**Figure 4:**
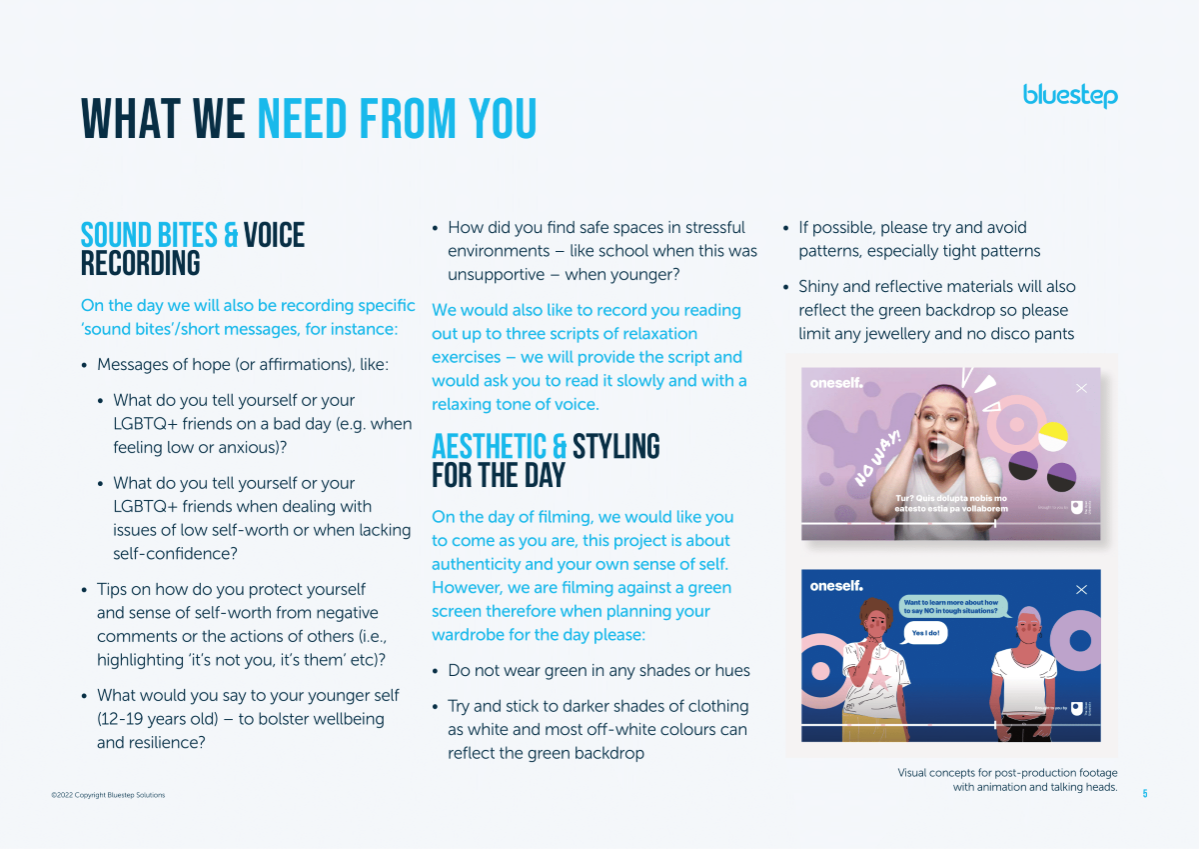
Creative briefing reviewed by sexual and gender minority contributors interested in supporting sexual and gender minority youth mental well-being during co-design processes in England (2022; part 2). LGBTQ+: lesbian, gay, bisexual, transgender, and queer.

Bluestep developed and circulated a creative brief for the 3 sexual and gender minority contributors explaining the requirements for filming (Figures 3 and 4).

Filming took place on November 29, 2022, in a London-based studio. On the day, all 3 sexual and gender minority contributors were asked the same questions on the topics of school, coming out, and friends and family (Multimedia Appendix 4). Filming was done against a green screen so that animations could be added later. Rough-cut footage included approximately 35 minutes of Chloe, Lilly, and Georgie each and 10 minutes of a group recording. ANG transcribed these rough cuts, which comprised 28 pages in total, and summarized their content into key points and quotes that could be shared with the young people. These were given to the sexual and gender minority youth in co-design workshop 4, who rated the points and quotes, adding their own reflections. For instance, the sexual and gender minority youth found Lilly’s advice to cope if someone reacts negatively to coming out helpful—she said the following: “Remember you are not alone. It may take time, but you’ll find your community and people that get you and understand you.” However, the sexual and gender minority youth found Georgie’s advice for teachers and students to manage bullying at school (ie, “zero tolerance” for this) too vague as most schools should have zero tolerance policies but there is still a need for proactiveness to enforce them. A summary of these points, organized by topic (coming out, school, and family and friends) and divided into challenges and solutions and strategies with key quotes to include, was then given to Bluestep to create 2- to 3-minute–long rough cuts of each video, which combined live footage and animation. This resulted in a total of 6 videos—*Parents and Families: Some Common Challenges*, *Parents and Families: Some Strategies*, *School: Some Common Challenges*, *School: Some Strategies*, *Coming Out: Some Common Challenges*, and *Coming Out: Some Strategies*. These were reviewed several times for content, design, storyline, accessibility, and subtitles and finally refined before approval by MFGL and ANG.

### The Oneself Resource

**Figure 5:**
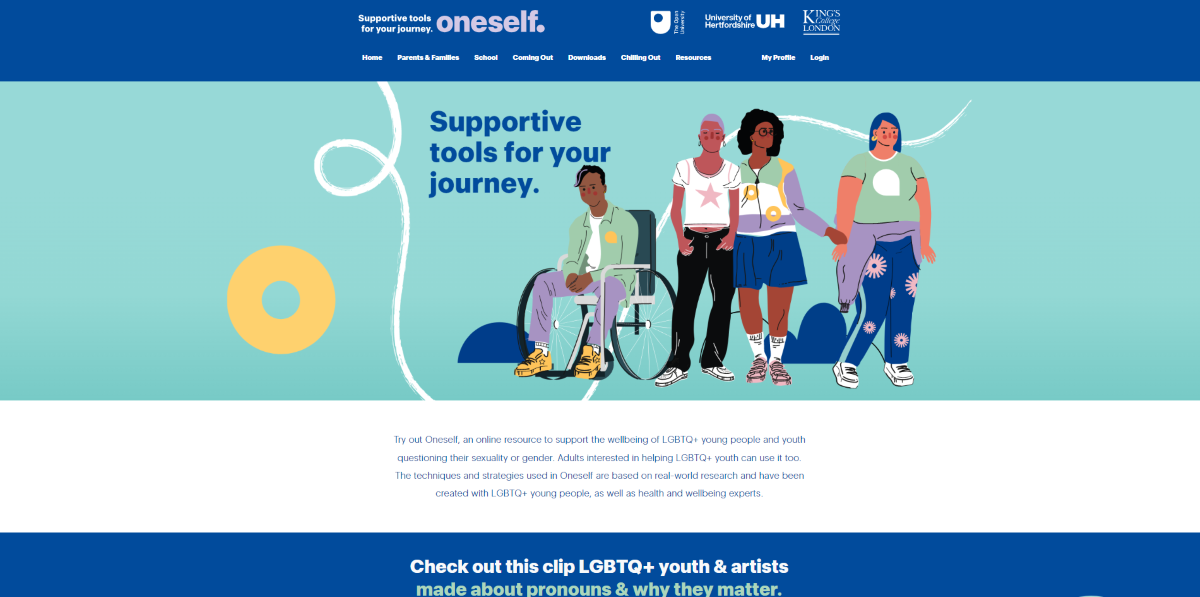
Part of the home page of Oneself that was developed together with sexual and gender minority youth interested in supporting sexual and gender minority youth mental well-being during co-design processes in England (2022).

Oneself was divided into 7 web pages: a home page (Figure 5); the 3 topics of parents and families, school, and coming out; downloads; chilling out; and resources. To access the entire toolkit, the user needs to log in and complete a brief baseline measure of well-being (ie, the 5-item World Health Organization Well-Being Index). The home page is free to access for anyone, although images of the contributors are reserved for the logged-in user.

The home page includes a description of *Oneself*; quotes; and extracts of what the user could find in the resource, for instance, the 3 topics. These were designed to prompt the user to log in to access the content. The home page also features an animation of the meaning and use of pronouns created in collaboration with sexual and gender minority youth from Rainbow Power (a sexual and gender minority youth group) run by the Free2B Alliance in England.

Each topic area began with two parts: (1) the problems and challenges that sexual and gender minority youth face in relation to that topic and (2) potential strategies and solutions to these issues. Each topic area included videos and social polls, which were then followed by activities, downloadable exercises, and external resources (see Multimedia Appendix 5 for an example).

Each topic area had 2 live footage videos: the first with sexual and gender minority contributors talking about common challenges on the topic based on their own experiences and the second with sexual and gender minority contributors talking about solutions, strategies, and advice on the topic based on their own experiences. Live footage was mixed with an animated background, highlighting what contributors were speaking about with color, illustrations, or additional text.

Each topic area also had 2 social polls, the first of which asked users to reflect on their own experiences on the topic. For example, for *Coming Out*, the question was as follows: “have you come out to others about your sexuality or gender yet?” A second social poll question asked users to reflect on which contributor’s experience was most similar to their own. After answering, the percentage of responses to the question became visible to the user. This was designed so that the user could understand others’ experiences and feel part of the *Oneself* community.

**Figure 6:**
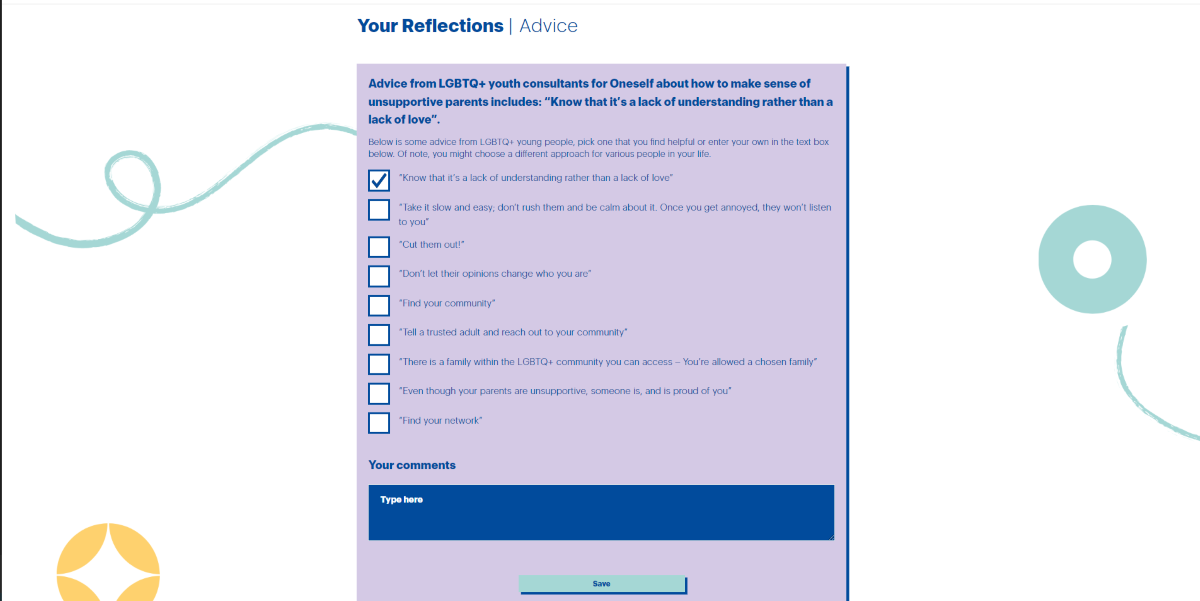
Example of a range of strategies presented after clicking on “explore more,” which was developed together with sexual and gender minority youth interested in supporting sexual and gender minority youth mental well-being during co-design processes in England (2022). LGBTQ+: lesbian, gay, bisexual, transgender, and queer.

In total, 2 exercises or activities per topic area were designed to help the user reflect on the topic in greater depth and learn more about how to implement strategies and advice in managing challenges. For instance, an exercise in “Parents and Families” is framed as follows: “Some LGBTQ+ young people have repeatedly described online environments as ‘lifesaving’ at times. Reflect on your experiences of creating an online support network for yourself.” This is followed by an “Explore More” button that takes the user to another page where they can read through several strategies and choose the ones that fit them best (Figure 6).

**Figure 7:**
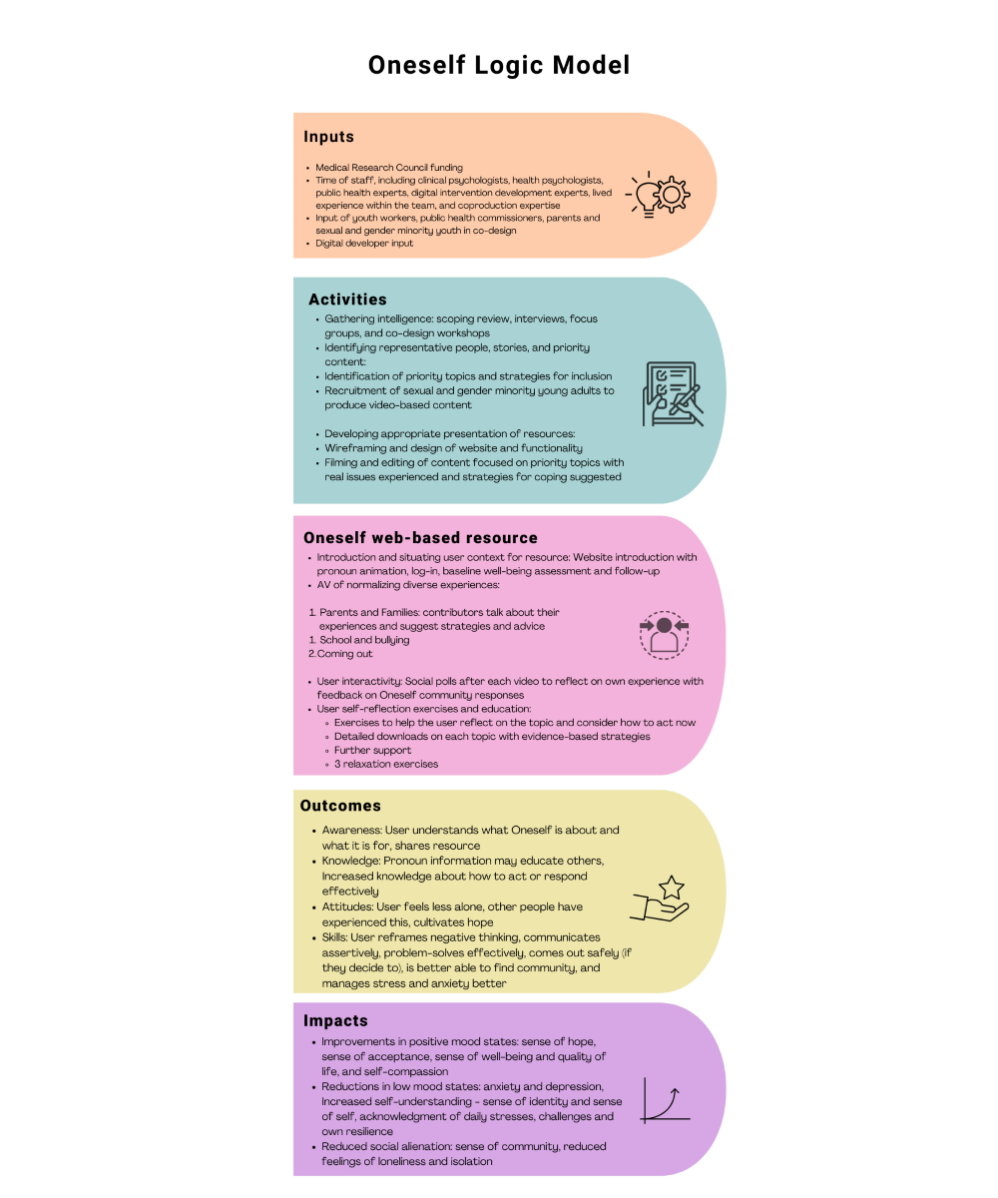
An overview of the co-design processes as these apply to the Oneself logic model as this pertains to sexual and gender minority youth mental well-being in England (2022). AV: audiovisual.

Downloads or downloadable exercises for each topic area were drafted by MFGL and ANG and then checked and refined by other research team members. Downloads were designed to tackle the problems and challenges described in each of the topics—2 downloadable guides addressing issues relevant to each topic provide detailed written information on strategies and solutions regarding them. In the case of *Parents and Families*, these look at standing up for yourself (communication) and problem-solving. In the case of *School*, they focus on finding allies and rejecting negativity (ie, the ABCD method). In the case of *Coming Out*, they support the coming out journey and finding hope. Downloads can also be found grouped together under the *Downloads* tab. An overview of the logic underpinning the development and content of Oneself is depicted in Figure 7.

Finally, each topic area provided 2 external resources leading to organizations and web pages that can offer sexual and gender minority youth further support, such as advice, community resources, or helplines. These were chosen in agreement with the research team. External resources can also be found grouped together under the *Resources* tab.

All content was interspersed with quotes from our sexual and gender minority youth participants and the 3 sexual and gender minority contributors as well as short comments and advice linked to the social polls.

**Figure 8:**
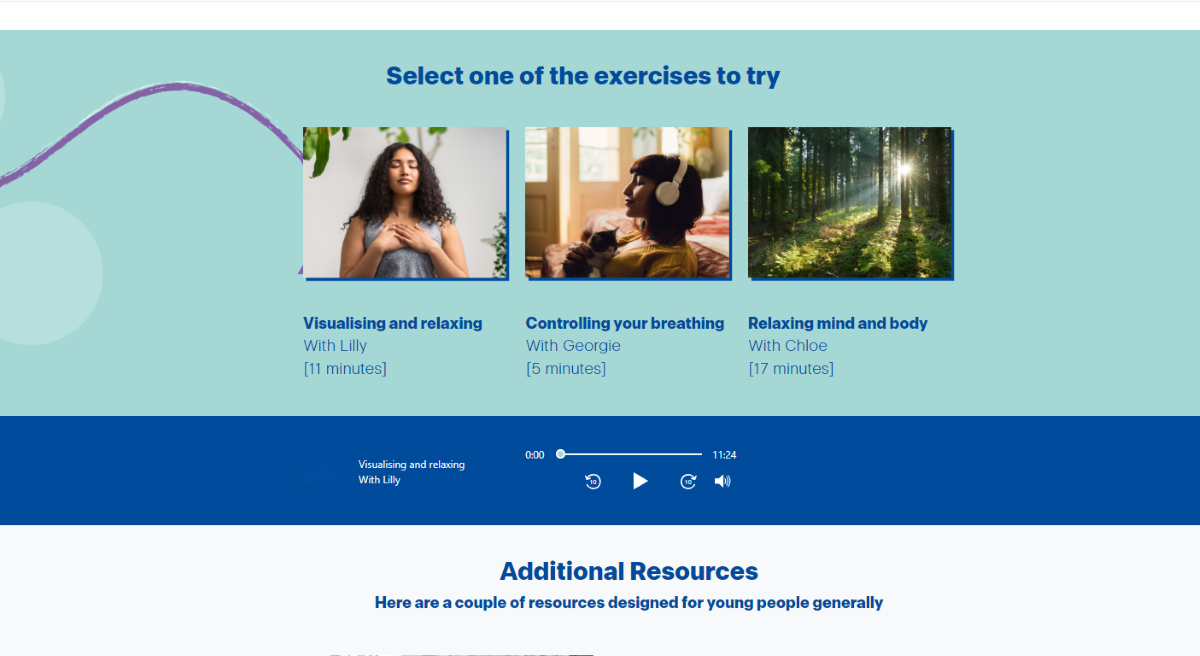
Screenshot of the relaxation exercises from the “Chilling Out” section that was developed together with sexual and gender minority youth interested in supporting sexual and gender minority youth mental well-being during co-design processes in England (2022).

Finally, a *Chilling Out* section was included promoting relaxation exercises as well as 2 additional external resources. These exercises consisted of 3 recordings, each led by a sexual and gender minority contributor following a script provided by the research team (Figure 8). A stress levels scale of 1 to 10 was available to complete before and after listening to each recording to help the user reflect on whether it had been a useful and calming exercise for them. *Oneself* was designed so that users can rate content with between 1 and 5 stars as they work through it, providing the research team with feedback.

## Discussion

### Principal Findings

This study aimed to set out the systematic and iterative approach undertaken to develop a web-based resource to support the mental well-being of sexual and gender minority youth so that they can deal effectively with the specific challenges of growing up LGBTQ+. Providing this kind of support was identified as important because sexual and gender minority youth are at greater risk of poor mental health outcomes than their cisgender and heterosexual peers [1,4,6,14]. This study demonstrated how project activities are mapped against the 6 stages of co-design set out by Hagen et al [13]. In particular, it showed how extant research evidence and engagement with a range of stakeholders and representatives of end users were drawn on to make decisions about the content and design of the final resource, named *Oneself*. The logic underpinning the content of the resource is also set out to support the design of future process and outcome evaluations of *Oneself*. Initial usability and end-user feedback has been gathered through a set of “think aloud” interviews and post use reflection interviews with sexual and gender minority youth and adult stakeholders. The findings of the latter study will be reported in detail elsewhere (MFG Lucassen, unpublished data, 2024). The feedback to date has been largely positive, with all sexual and gender minority youth testers saying they would recommend the resource to others. There have also been some points of constructive and critical feedback, in particular from adult stakeholders, which will need to be considered in future development work (eg, that a greater range of experiences should be included, such as those of cisgender gay male individuals and individuals from minority faith backgrounds).

### Strengths and Limitations of Oneself

There are several strengths and limitations to *Oneself* in its current format. Strengths include the fact that *Oneself* represents one of the first digital mental well-being–related resources reported on that is designed specifically to meet the needs of sexual and gender minority youth. The design drew on the evidence base for techniques to support their well-being [14] and was developed in collaboration with 4 different stakeholder groups: sexual and gender minority youth, adults who work with sexual and gender minority youth, parents of sexual and gender minority youth, and commissioners of public health services focused on their needs. In doing so, the process of development included a wide variety of relevant perspectives and looked to build on what is already known about supporting the well-being of this population. It is also a strength of the resource that its development included adolescents aged <16 years and was inclusive of gender and sexual minority groups rather than focusing solely on gender or sexual minority groups. This is a departure from previous interventions that have typically focused on those aged >16 years only and selected to focus on either gender or sexual minority groups [14]. Although there are important differences between sexual and gender minority experiences, there is also considerable overlap, including compromised mental well-being for many. Some young people will ultimately identify as being both a sexual and gender minority, which makes the resource’s recognition of both groups important.

Limitations of the resource include the fact that, given the budget constraints, much of the available funding had to be channeled into creating basic initial functionality that would be likely to engage and sustain interest from the target end users. This meant that much of the evidence-informed content that we might expect to have the greatest effect on mental health and well-being had to be included within the more text-heavy “downloads” section. Although, in early consultation work, sexual and gender minority youth suggested that these “downloads” were a good way to provide additional resources for use offline, it was later acknowledged that young people do not want to have to read a lot of text when engaging with the content (MFG Lucassen, unpublished data, 2024). Common evidence-based features for supporting mental health and well-being include relaxation exercises [28], behavioral activation [29], problem-solving [28-30], helping people recognize problematic cognitions [26], and cognitive restructuring [26]. Future iterations will need to focus on bringing more of this content into the interactive elements of *Oneself.* However, in doing so, it will also be important to consider whether such features are best delivered via pure self-help or whether optimal delivery requires engagement with an adult who can help structure what are often quite complex therapeutic activities (eg, sexual and gender minority youth can feasibly be supported by “e-coaches” to complete resources such as *Oneself*).

The sexual and gender minority youth involved in co-designing *Oneself* included almost one-quarter of individuals who were of dual heritage (eg, European and West African) or from a migrant background (eg, several of the White participants). Furthermore, gender minority youth, who have been traditionally underrepresented in LGBTQ+ research [31], were very well represented, as were bisexual and pansexual participants. Nonetheless, content could have been improved in relation to intersectionality, such as the fact that there is a need to represent sexual and gender minority youth more complexly in terms of sexual and gender minority youth’s social positions (eg, across ethnicity, religion, and social class). Future iterations need to look at making the resource more relatable to additional underrepresented groups, as was suggested during co-design processes (eg, for care-experienced Asian sexual and gender minority youth from a minority faith background), who may face different and complex challenges growing up as sexual and gender minority youth.

Self-help digital resources and interventions have the potential to be very cost-effective [32]. They can be relatively low cost to produce, with the potential for very high reach given evidence of increasing digital access and capability, particularly among young people [10,11]. Despite this, it is likely that those with the greatest vulnerabilities and at the most risk of poor mental well-being may be the least likely to access suitable web-based spaces with ease (eg, those with limited funds to purchase data for a mobile phone). Therefore, reaching these individuals needs to be carefully considered by those who are responsible for identifying and tackling such needs, including youth support organizations, schools, and commissioners of services. In addition, digital resources such as *Oneself* need to keep up with the rapid pace of progress and evolution in the web-based world. Young people have high expectations and are savvy consumers of web-based media, and they anticipate polished and engaging products. Keeping a resource such as *Oneself* comprehensive, up-to-date, and relevant in terms of content and look and feel requires ongoing funding. Relatedly, sexual and gender minority youth highlighted the importance of educating others, in particular teachers and parents, as this would bolster their overall mental well-being. In future funded work, we would like to develop resources specifically for adults, potentially within the overall *Oneself* intervention. Finally, something we identified that we would not be able to achieve with *Oneself*, at least for now, was direct access to support and interaction from a sexual and gender minority youth peer group. Although this was desired, providing it would involve considerable resources to monitor and approve content and messaging and avoid harm that could be caused by web-based bullying and harassment. Investigating how to provide this sense of community more fully in a web-based space warrants further attention. Ideally, such spaces should be structured in such a way that the experiences of sexual and gender minority youth can be shared without any pressure to divulge information that could identify a young person or lead to instances of “oversharing” (which sexual and gender minority youth may regret at a later stage). Case studies, as presented in *Oneself* with the contributors, could offer a safe means to discuss personal issues without the need for self-disclosure. We think that establishing and maintaining web-based community spaces in the context of digital mental health technologies requires further study to ensure that such spaces are both acceptable and viable. However, a noteworthy shortcoming of direct access to ongoing human support and interaction given the associated costs and practical considerations (eg, whether an intervention can realistically be provided 24 hours a day, 7 days a week) are limitations in terms of an intervention’s likely reach.

### Strengths and Limitations of the Research

The research we have conducted in developing *Oneself,* and this paper specifically, makes an important contribution to needed literature that opens the “black box” of intervention development [33]. Attempting to record the process of development, including the co-design stages, as accurately and comprehensively as possible and placing it within the public domain via open access publishing contributes to the Open Science Agenda by making it accessible, inclusive, and transparent [34]. Being explicit about the logic that underpins the intervention content in terms of how it is intended to have an effect on factors associated with maintenance (or not) of mental well-being is also important to support the design of future evaluation studies [35].

Co-design work is complex and challenging to do well. We believe that aspects of our co-design efforts were of merit, in particular our inclusion of younger sexual and gender minority youth (which included participants as young as 12 years of age) and our engagement of sexual and gender minority youth from the “Identify” all the way through to the “Use” stages of the process [13]. We drew heavily on sexual and gender minority youth’s views to decide on the topic areas to focus on and in deciding on the “look and feel” of *Oneself*. We also made key changes to the resource in response to sexual and gender minority youth feedback, such as not using dramatizations, as was initially envisaged. Challenges to the co-design processes included the COVID-19 pandemic at the start of the project, which meant that work with sexual and gender minority youth was conducted using videoconference software at a time when adolescents were frequently fatigued by web-based forms of communication. Connected to this was our awareness that assisting in the creation of *Oneself* was one of the many demands placed on the sexual and gender minority youth involved in co-design, and as such, we sought to use sexual and gender minority youth’s time efficiently. Consequently, we limited the number of workshops conducted and carried out some consultations via email, which was less robust. In the future, we could enhance our co-design efforts and move closer to partnership (as opposed to consultation as defined by Arnstein [36] in her ladder of participation) by helping a number of older sexual and gender minority youth learn more about evidence-based techniques for supporting mental well-being and subsequently getting them to design features of the content. These older adolescents could be employed as coresearchers, and they could draft and further develop content with our ongoing support.

Most sexual and gender minority youth involved in the co-design of *Oneself* were gender minority young people, which is a strength given that these youth are underserved by mental health services [4]. However, a limitation of our research was that we struggled to recruit cisgender adolescents to join the co-design workshops and, as such, may have underrepresented the views or specific needs of certain youth (eg, cisgender lesbian and gay youth). Relatedly, it is likely that some groups or individuals who may need intervention support the most are among those least likely to get involved in co-design or research activities (eg, sexual and gender minority youth who do not feel safe to “come out”) leading to intervention development and associated research more generally, which misses the perspective of those who are “not out.” Acknowledging this potential issue is important, and striving to reach the underheard and underserved must remain a priority of future research.

### Summary, Conclusions, and Next Steps

This study aimed to set out the process involved in co-designing and developing *Oneself*, a digital resource to support sexual and gender minority youth in building and maintaining their resilience to cope with the everyday challenges of growing up LGBTQ+ and support their mental health and well-being more generally. It is hoped that, in the future, this resource will be extended so that it is also of use for educating adults who wish to support sexual and gender minority youth. We have explained the included content and the logic that underpins its use and acknowledged a range of strengths and limitations of what has been achieved so far. Priorities for future efforts will be to specifically address critique and feedback provided by adults and sexual and gender minority youth during their “think aloud” interviews (MFG Lucassen, unpublished data, 2024); build in additional characteristics translating evidence-based content into interactive features; and continue to incorporate diverse voices in co-design, including consideration of how intersectionality may need to be more integrated. The next steps include applying for further research funding to continue our evaluation and development activities.

## Appendix

Multimedia Appendix 1 Concept design options for Oneself.

Multimedia Appendix 2 Questionnaire for sexual and gender minority youth as part of co-design of Oneself.

Multimedia Appendix 3 Wireframe website map for Oneself.

Multimedia Appendix 4 Instructions given to sexual and gender minority contributors for filming video content.

Multimedia Appendix 5 Screenshots from Oneself.

## Abbreviations

LGBTQ+: lesbian, gay, bisexual, transgender, and queer
OU: The Open University
PRISMA-ScR: Preferred Reporting Items for Systematic Reviews and Meta-Analyses extension for Scoping Reviews

## Data Availability

The data sets generated during and analyzed during this study are not publicly available due to their sensitive nature but are available from the corresponding author on reasonable request.
