# Supplementary material for: A Web-Based Intervention to Support the Mental Well-Being of Sexual and Gender Minority Young People: Mixed Methods Co-Design of Oneself
Source: JMIR Form Res. 2024 May 21;8:e54586. doi: 10.2196/54586 (PMC11150889; doi:10.2196/54586)
Supplement: Multimedia Appendix 1 [file formative_v8i1e54586_app1.pdf]

EVERYTHING BEGINS WITH AN *idea...*

OPEN UNIVERSITY | TOOLKIT IDENTITY & NAMING

bluestep
Design & Merchandise

toolkit VALUES

We have defined the tooklit values by representing them in the form of vision boards.These represent what we have outlined, enabling us to understand the true visual essance of the toolkit, further refining our brief and the overall aim of the online support. We have also defined how these values will sound when communicated to users, in a less formal and more conversational way. These phrases will be used when writing the content of the tooklit.

INCLUSIVE

VISUALISED TOOLKIT VALUES

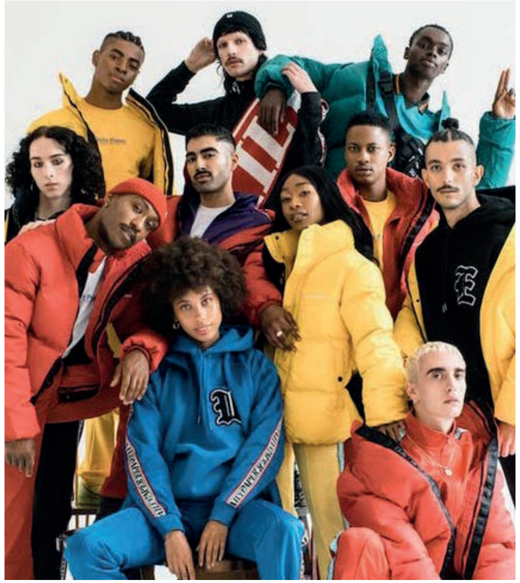

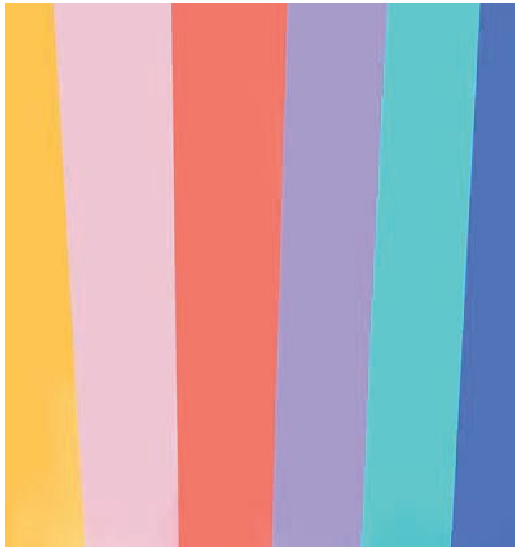

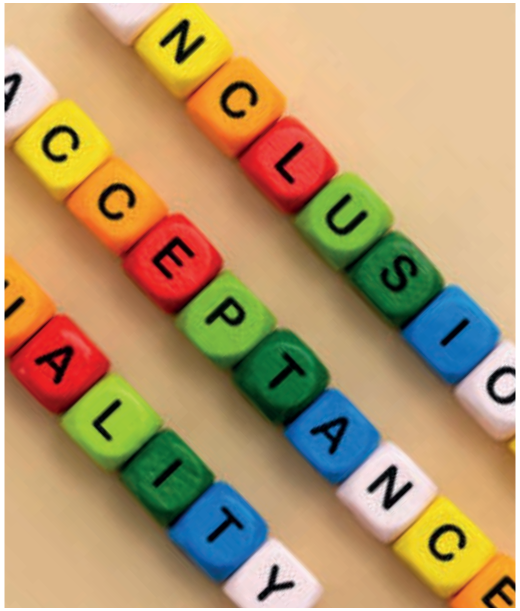

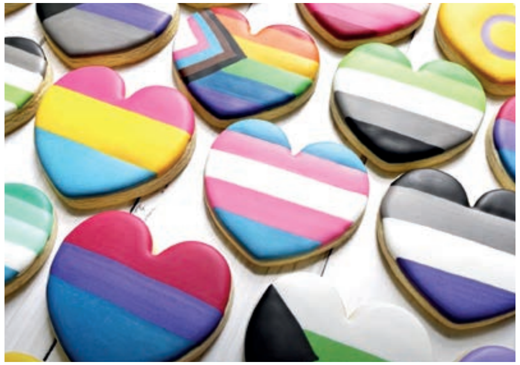

TONE OF VOICE:

WE'RE ALL IN

©2022 Copyright Bluestep Solutions

SAFE

VISUALISED TOOLKIT VALUES

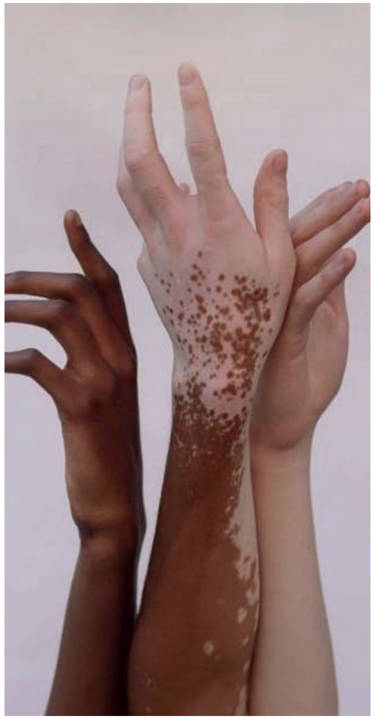

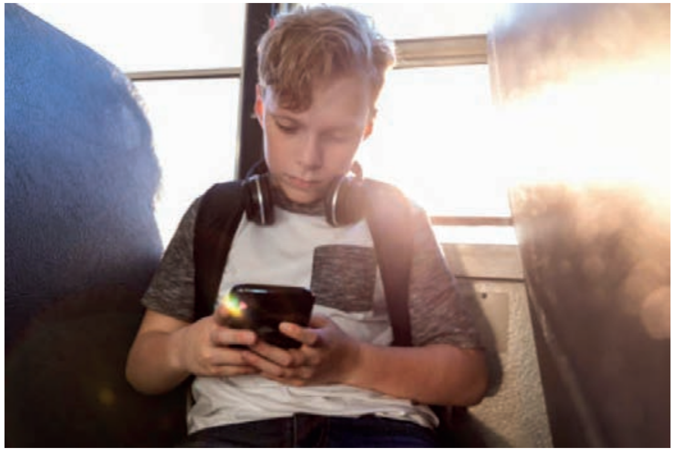

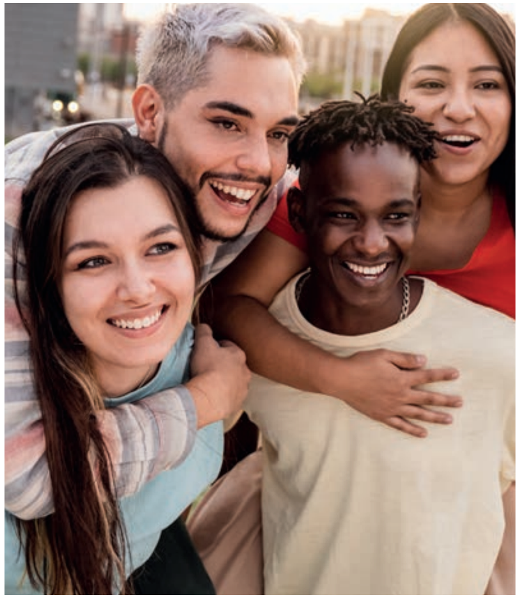

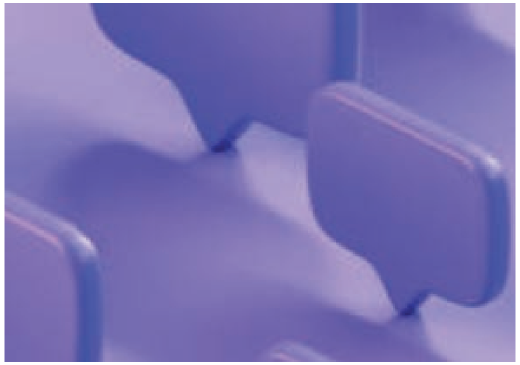

TONE OF VOICE:

BE YOURSELF & HELP YOURSELF

©2022 Copyright Bluestep Solutions

POSITIVE

VISUALISED BRAND VALUES

bluestep

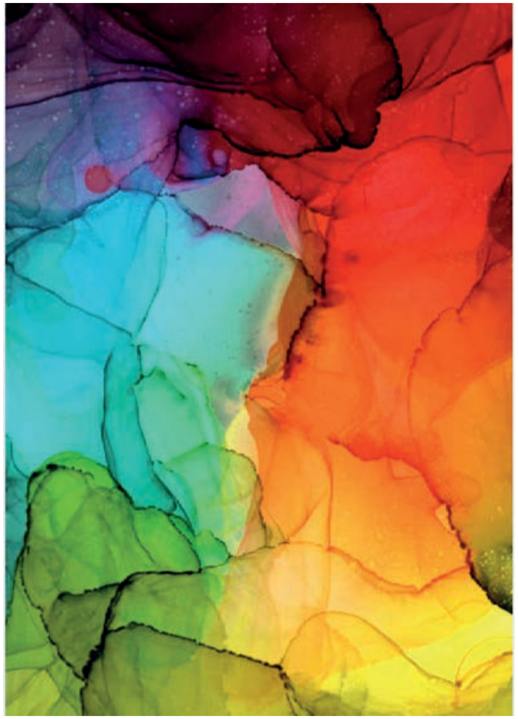

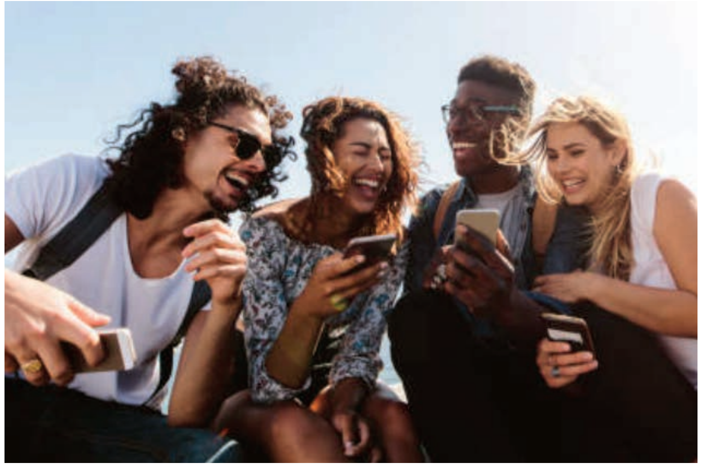

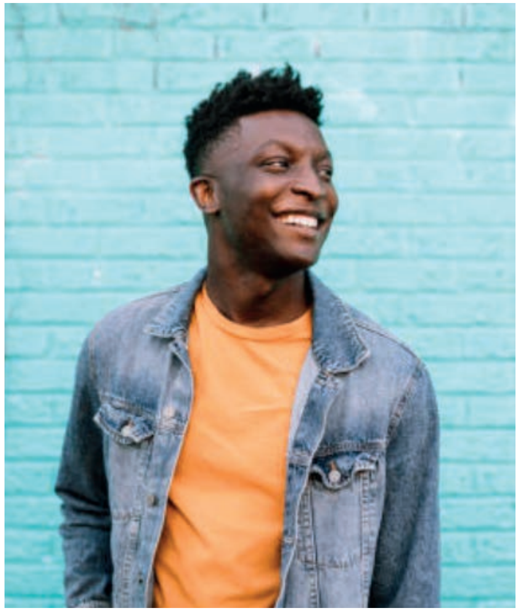

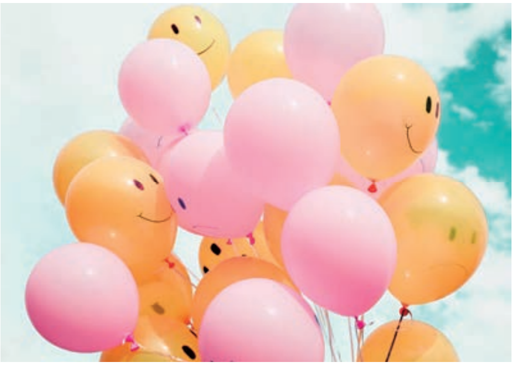

TONE OF VOICE:

HAPPY VIBES ONLY

©2022 Copyright Bluestep Solutions

AUTHENTIC

VISUALISED TOOLKIT VALUES

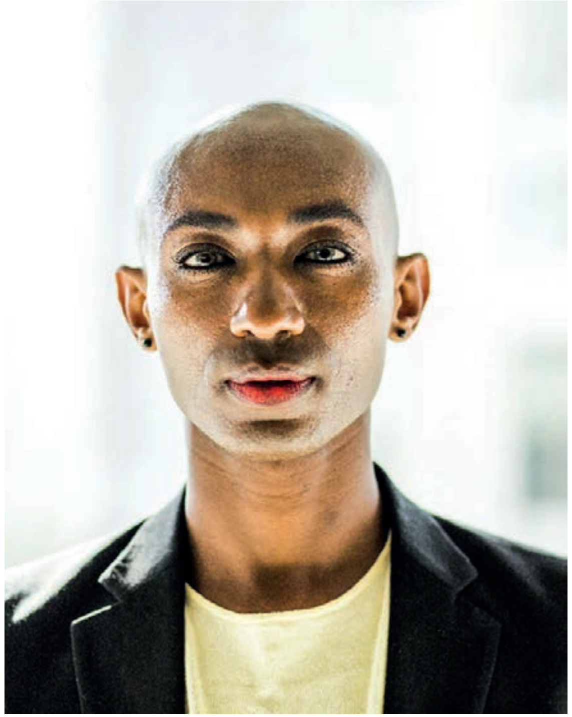

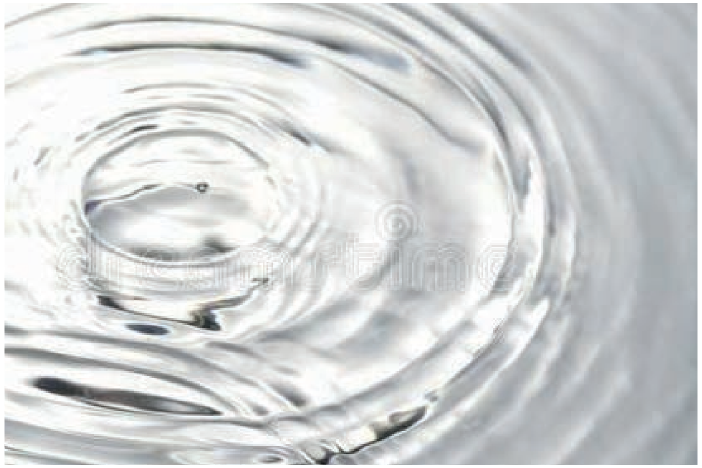

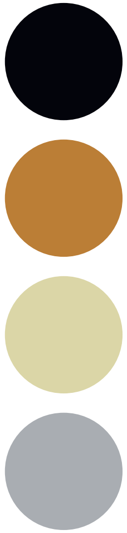

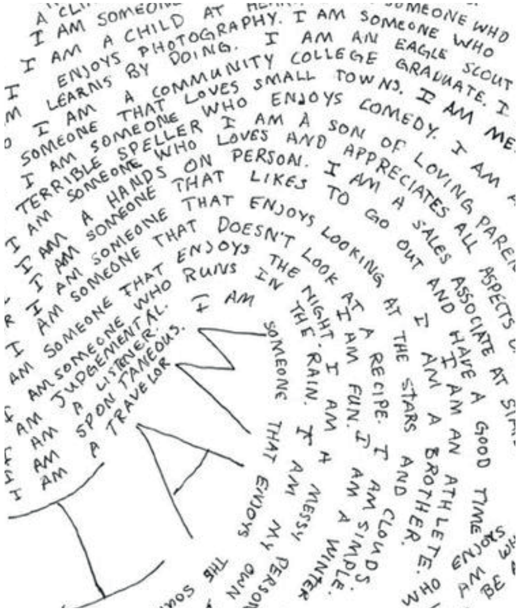

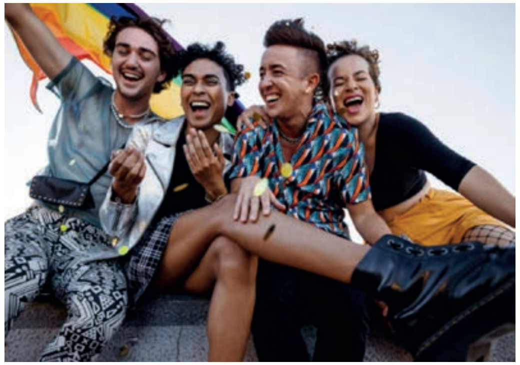

TONE OF VOICE:

ABSOLUTELY NO BS

©2022 Copyright Bluestep Solutions

THE TOOLKIT **IS:**

VISION BOARD

bluestep

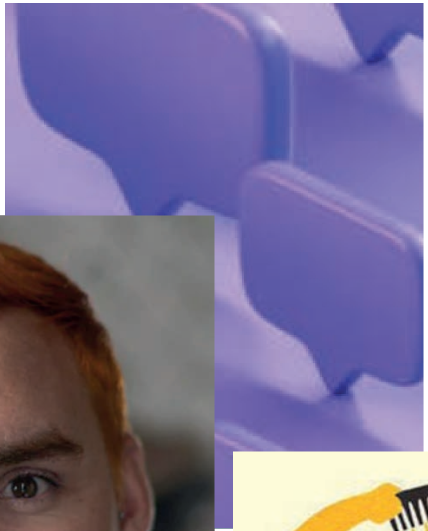

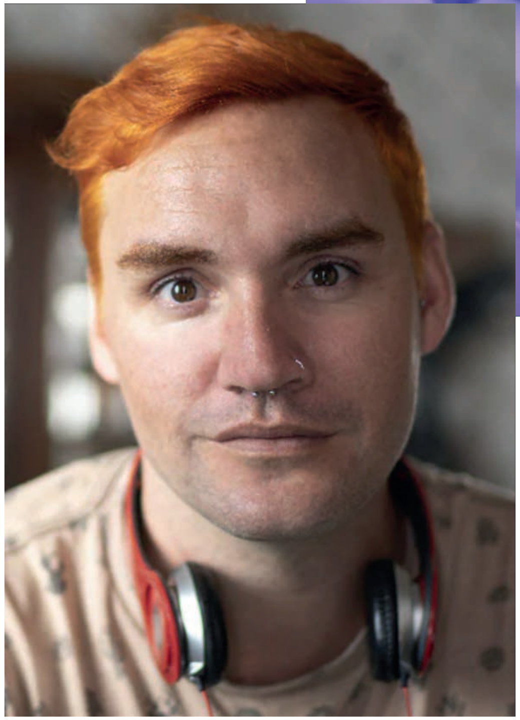

brook

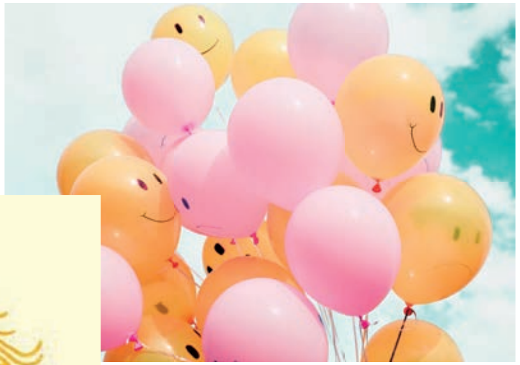

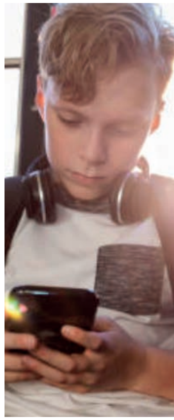

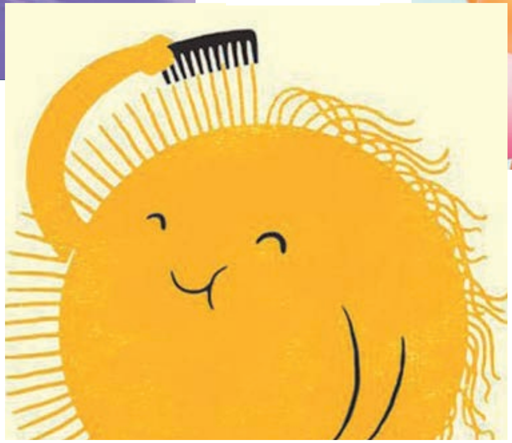

INCLUSIVE

WARM

CALM

APPROACHABLE

INFORMATIVE

SUPPORTIVE

WILDFANG

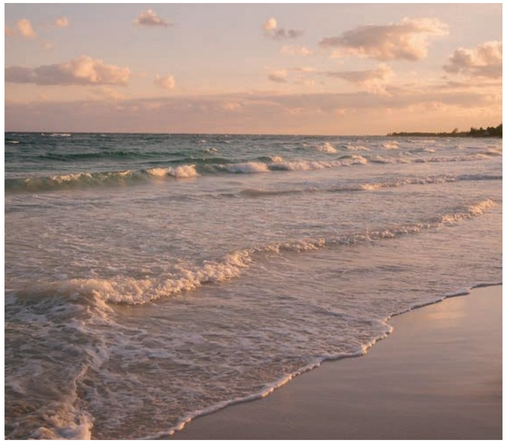

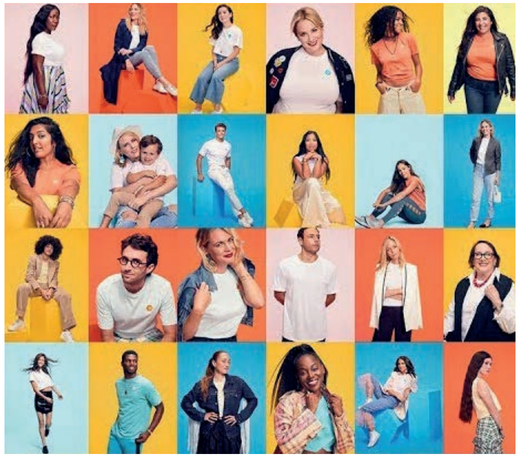

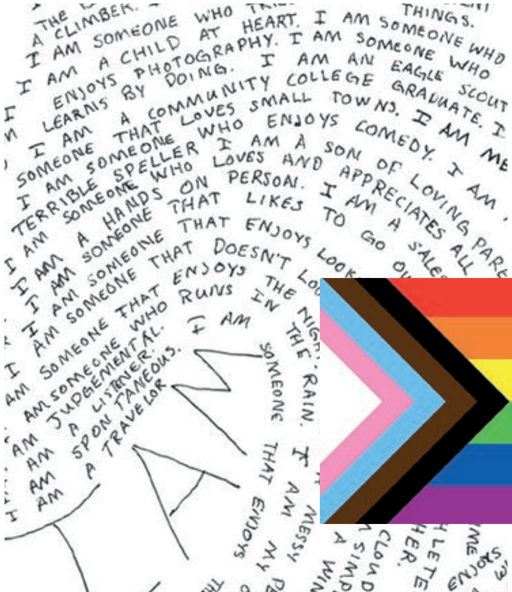

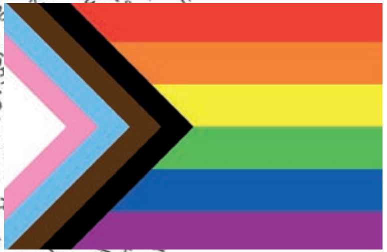

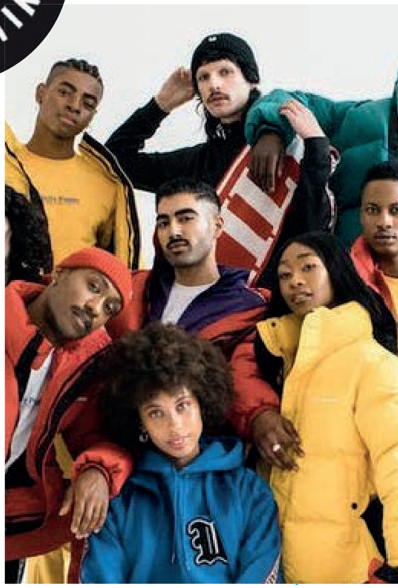

©2022 Copyright Bluestep Solutions

THE TOOLKIT **IS NOT:**

VISION BOARD

CLINICAL

DIRECT

AUTHORITATIVE

NEGATIVE

COLD

JUDGEMENTAL

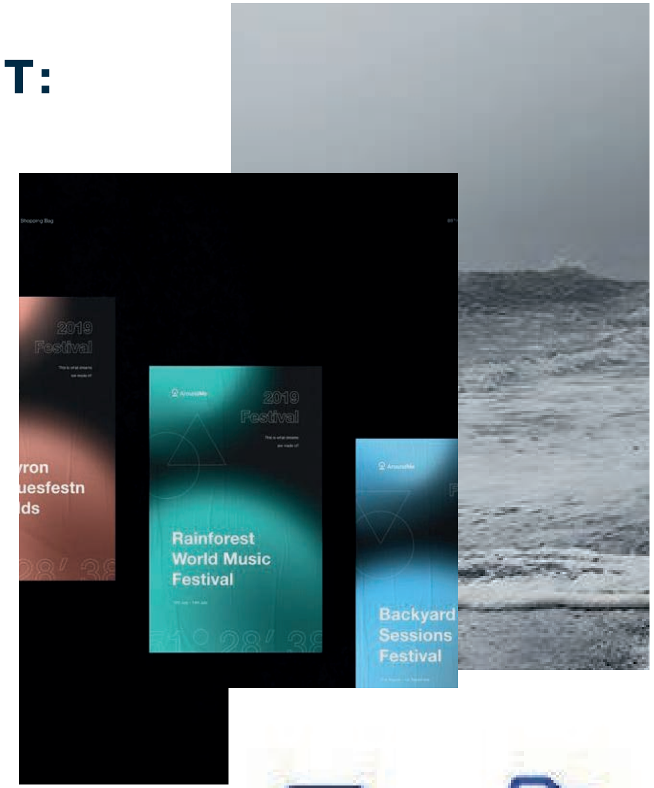

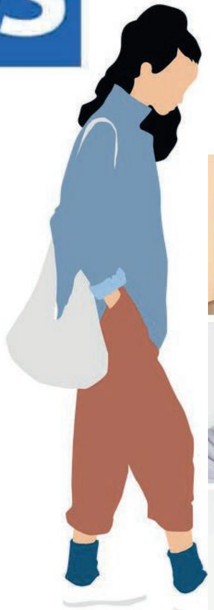

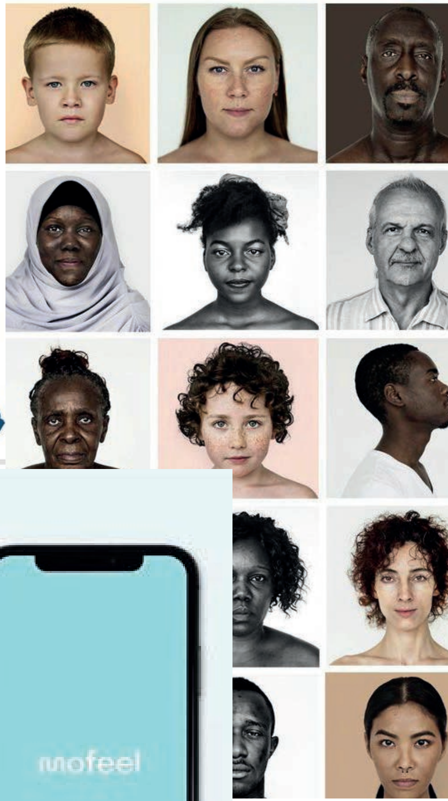

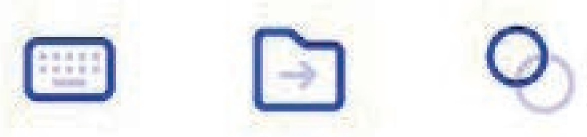

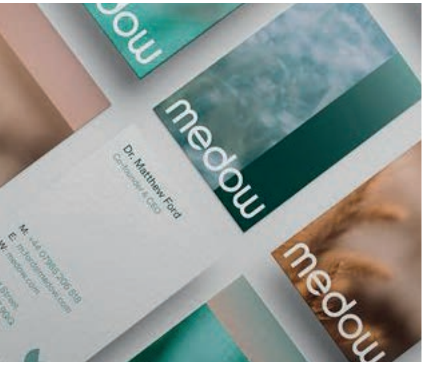

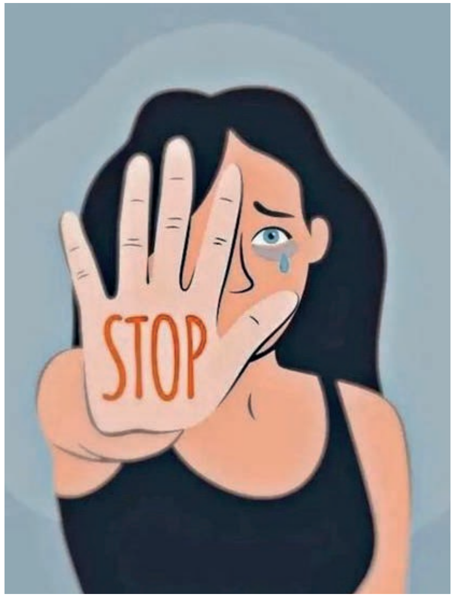

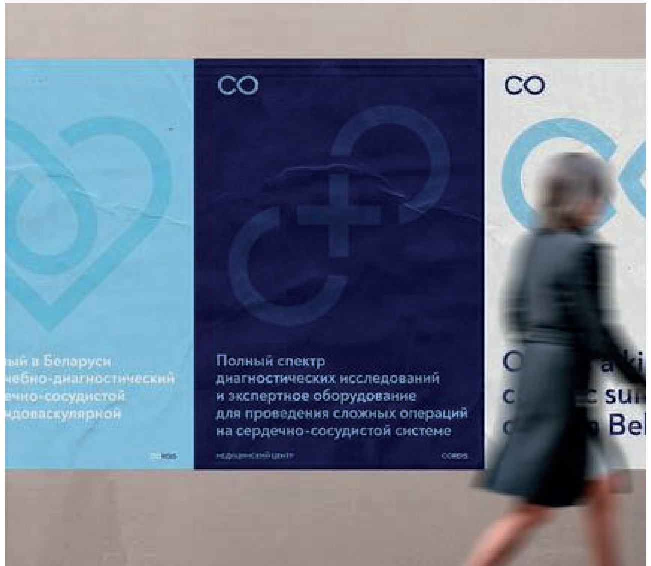

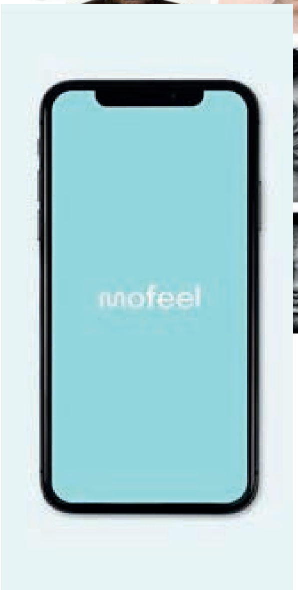

©2022 Copyright Bluestep Solutions

concept TERRITORIES

The concept routes we have developed explore the depicted visual territories within these vision boards. The vision boards are compiled of our research into existing design surrounding similar topics and resources. This helped us when producing the creative so we could make sure not to repeat existing work, but also inspired us to produce creative assets on a similar wavelength.

TOOLKIT IDENTITY CONCEPT TERRITORIES

COMTEMPORARY

A popular look and feel for like minded causes is 'contemporary' featuring bold colours and sans serif fonts. This creates a positive vibe and engages audiences with bold graphics and statements.

bluestep

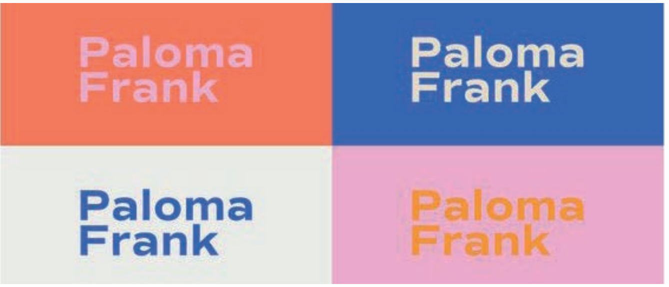

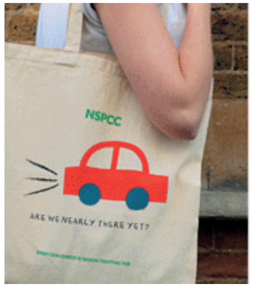

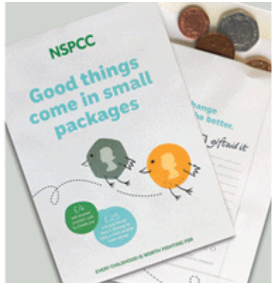

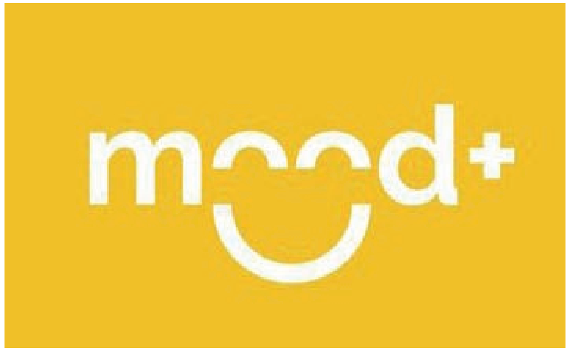

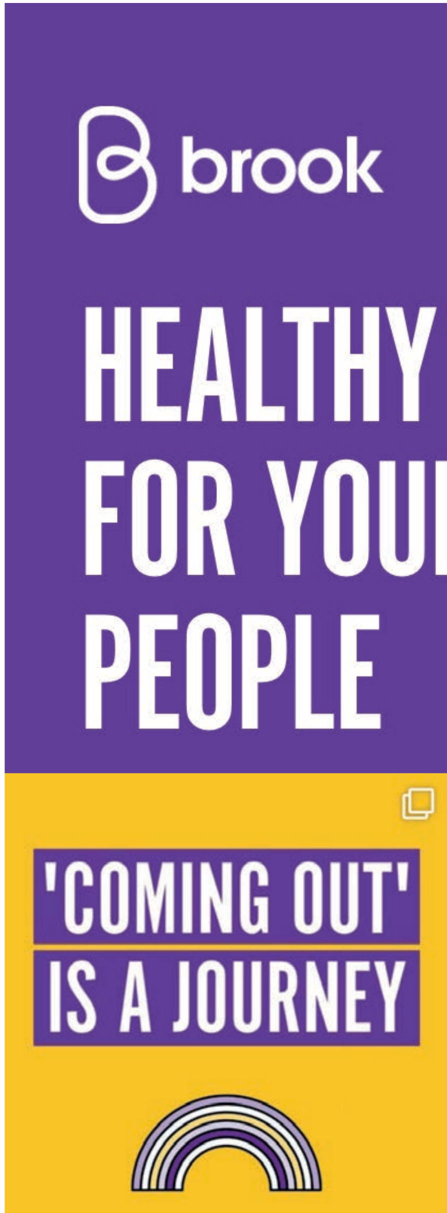

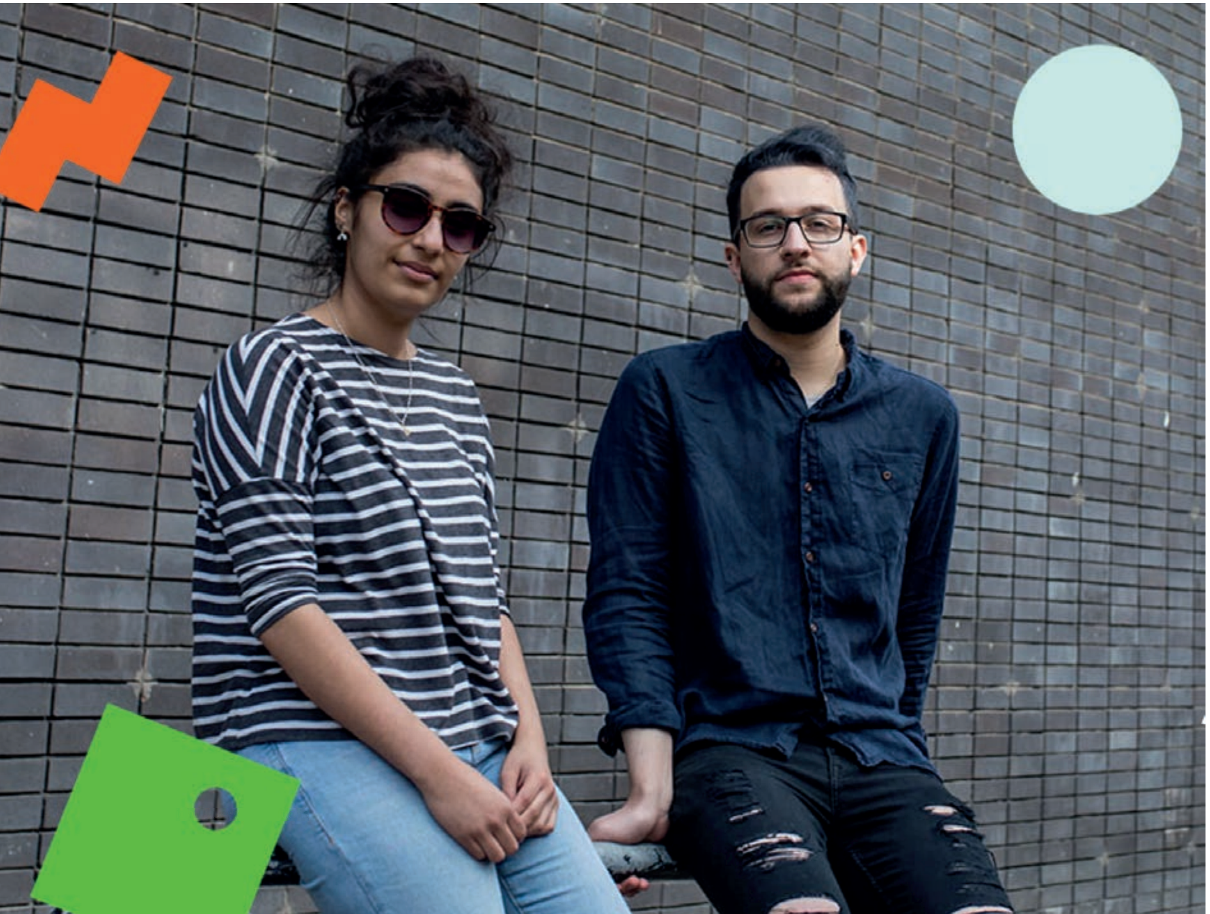

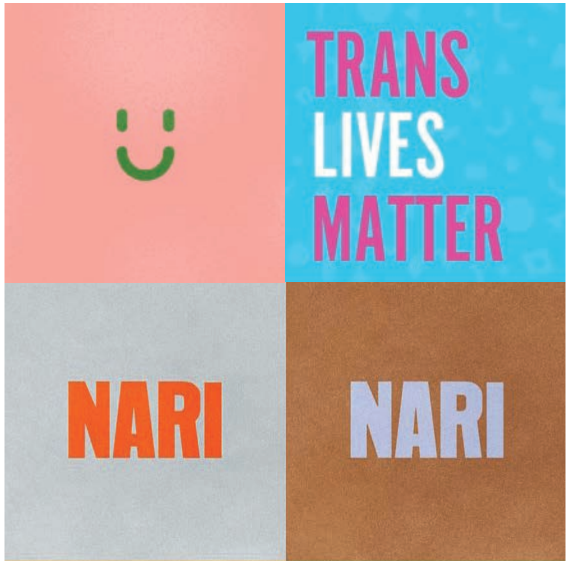

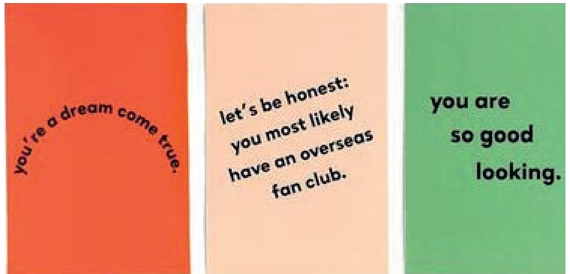

TOOLKIT IDENTITY CONCEPT TERRITORIES

ORGANIC

Equally, a more organic look and feel is popular amoungst like minded causes. These feature a more hand finished approach, with scripted fonts and doodle illustrations. This creates an approachable energy and engages audiences with friendly soft graphics and statements.

bluestep

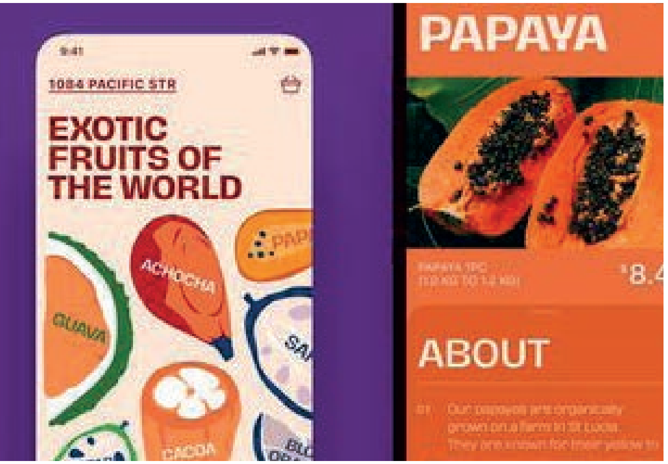

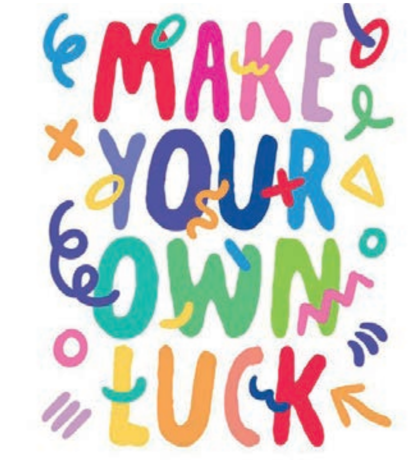

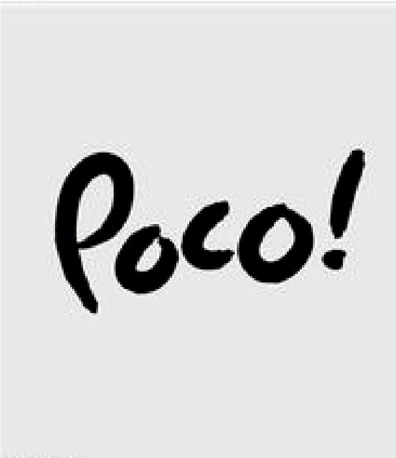

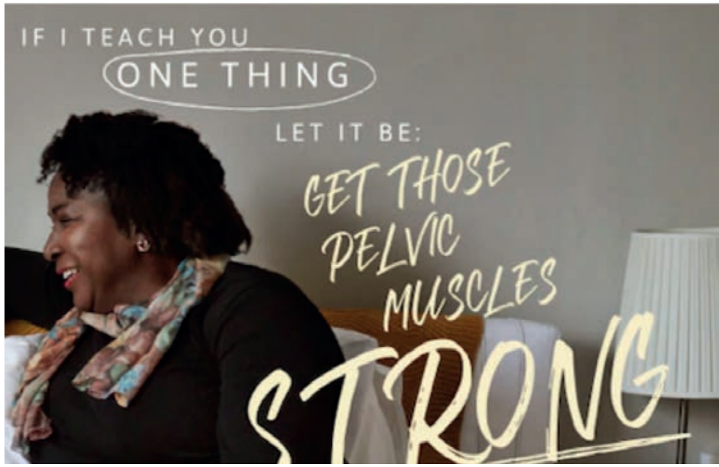

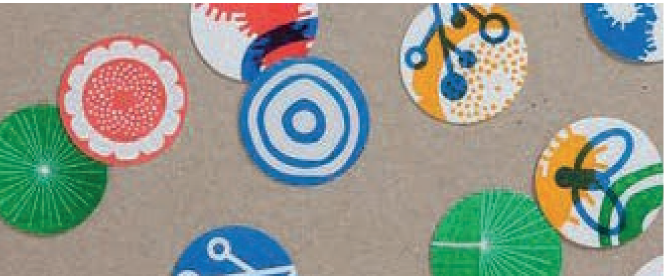

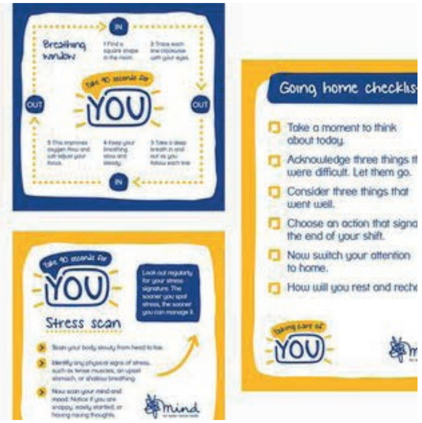

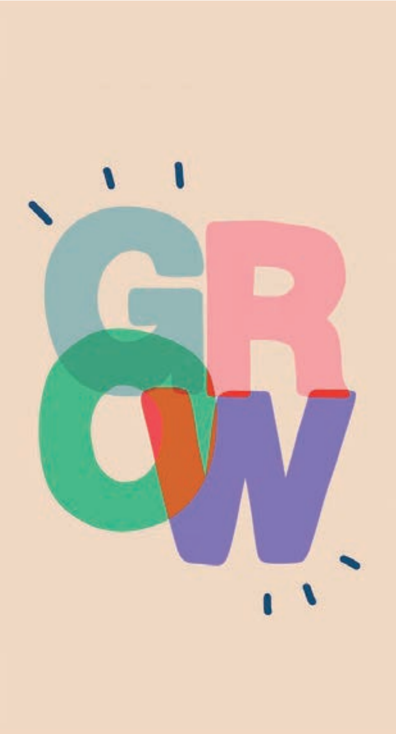

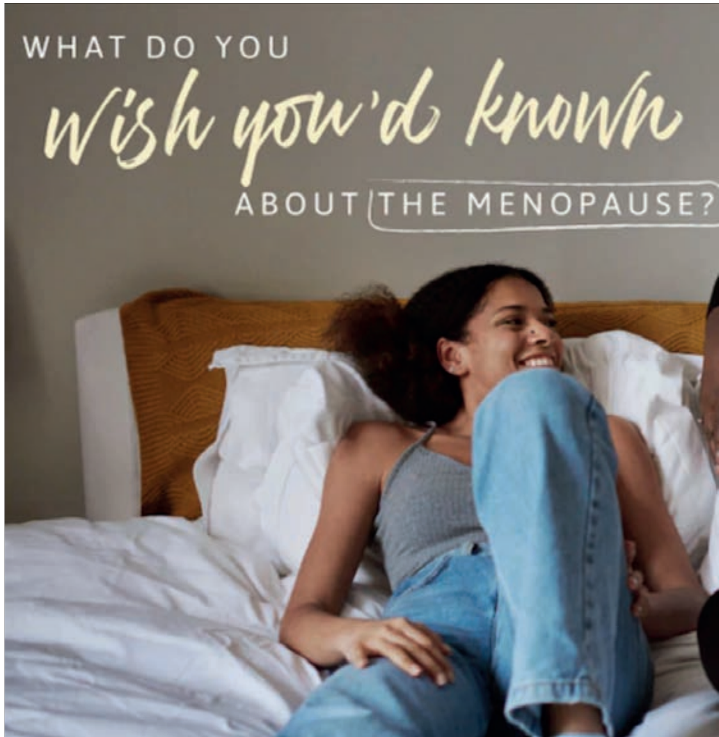

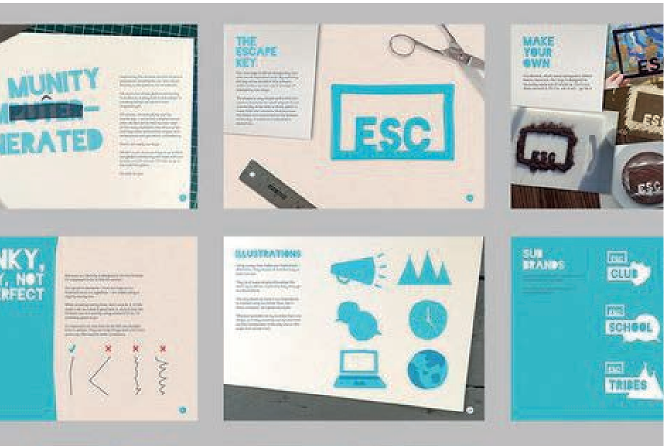

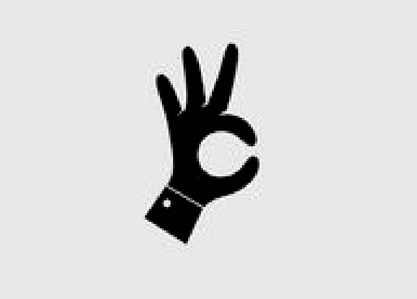

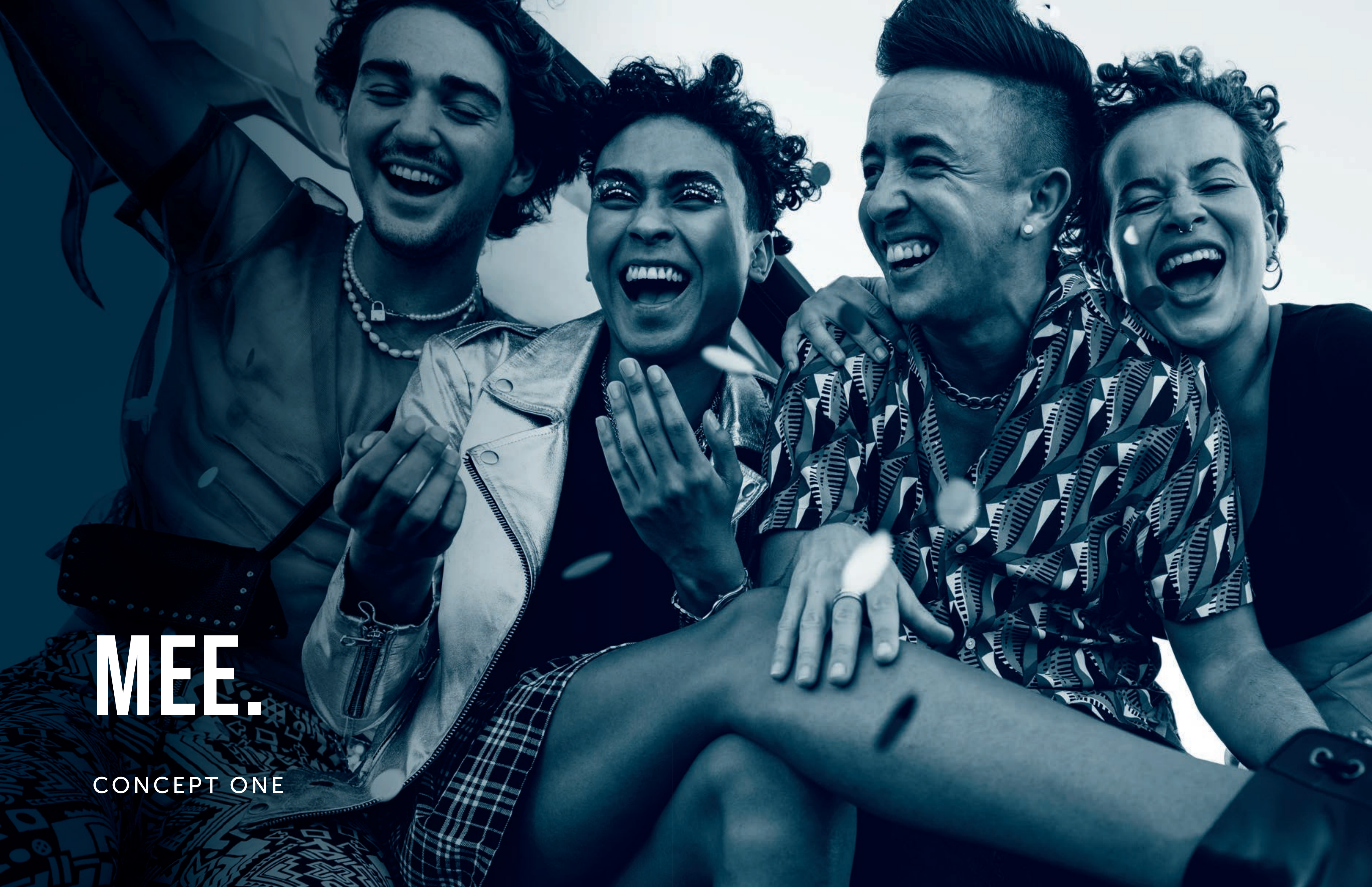
MEE.
CONCEPT ONE

CONCEPT ONE

TOOLKIT IDENTITY

The toolkit identity has been developed using a clean sans serif typeface. Focus is drawn to the identity name using The Open Universities master brand colour in order to create synergy with the tooklit and the organisation.

bluestep

Mindful Education and Enlightenment for LGBTQ+

mee.

Brought to you by The Open University

CONCEPT ONE

TOOLKIT IDENTITY COLOURS

Based on the results of the workshops a pastel colour pallet has been explored. The master brand colours consist of the OU blue and a pink pastel, the master colours hint towards the trans pride flag colours. The secondary pallet is made up of fresh, clean hues that compliment the master tones.

bluestep

C: 99
M: 73
Y:0
K:0

R: 1
G: 75
B: 156

C: 0
M: 30
Y:10
K:0

R: 248
G: 200
B: 208

C: 0
M: 0
Y:0
K:100

R: 0
G: 0
B: 0

C: 0
M: 0
Y:0
K:0

R: 255
G: 255
B: 255

C: 38
M: 0
Y:34
K:0

R: 172
G: 214
B: 187

C: 40
M: 0
Y:20
K:0

R: 165
G: 215
B: 213

C: 30
M: 40
Y:0
K:0

R: 188
G: 163
B: 206

C: 0
M: 20
Y:65
K:0

R: 254
G: 209
B: 210

Brought to you by The Open University

CONCEPT ONE

TOOLKIT IDENTITY
FOR THE VISUALLY IMPARED

We've adapted the pastels to bright colours, as these are generally the easiest to see because of their ability to reflect light. Solid, bright colours such as red, orange, and yellow are usually more visible than pastels.

bluestep

C: 99
M: 73
Y:0
K:0

R: 1
G: 75
B: 156

C: 0
M: 30
Y:10
K:0

R: 248
G: 200
B: 208

C: 0
M: 0
Y:0
K:100

R: 0
G: 0
B: 0

C: 0
M: 0
Y:0
K:0

R: 255
G: 255
B: 255

C: 55
M: 0
Y:70
K:0

R: 130
G: 192
B: 111

C: 60
M: 0
Y:10
K:0

R: 93
G: 196
B: 225

C: 70
M: 90
Y:0
K:0

R: 110
G: 54
B: 140

C: 5
M: 20
Y:80
K:0

R: 254
G: 209
B: 210

Brought to you by The Open University

CONCEPT ONE

TOOLKIT IDENTITY ICONS

A selection of contemporary icons have been created to express different emotions and key words. This helps to break down and simplify otherwise complex emotions into a more geometric visual format.

bluestep

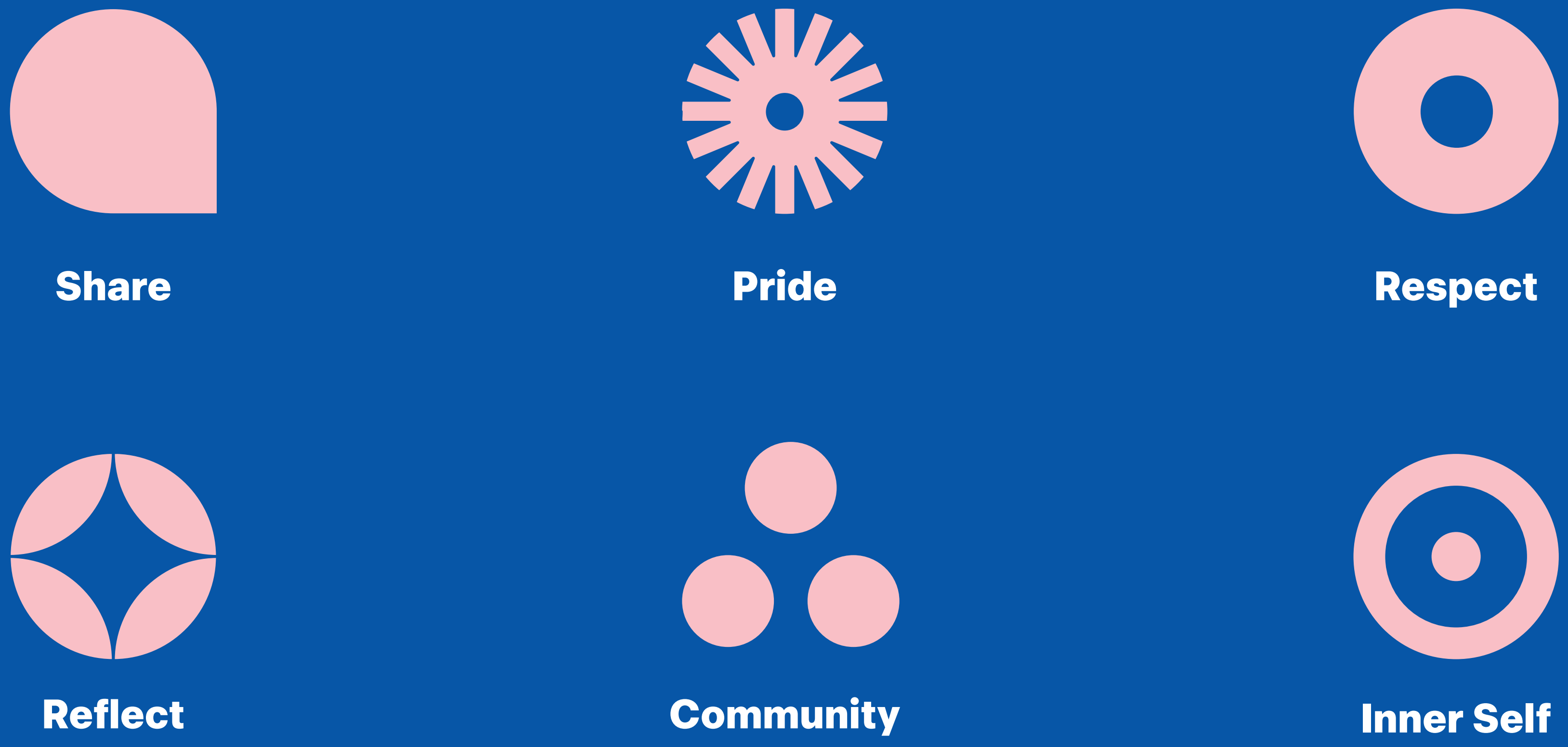

CONCEPT ONE

TOOLKIT ONLINE PLATFROM
(MOBILE VIEW)

When combined the elements of the tookit identity create a soft, welcoming platform for users to navigate. With bold captions, and clear call to actions.

bluestep

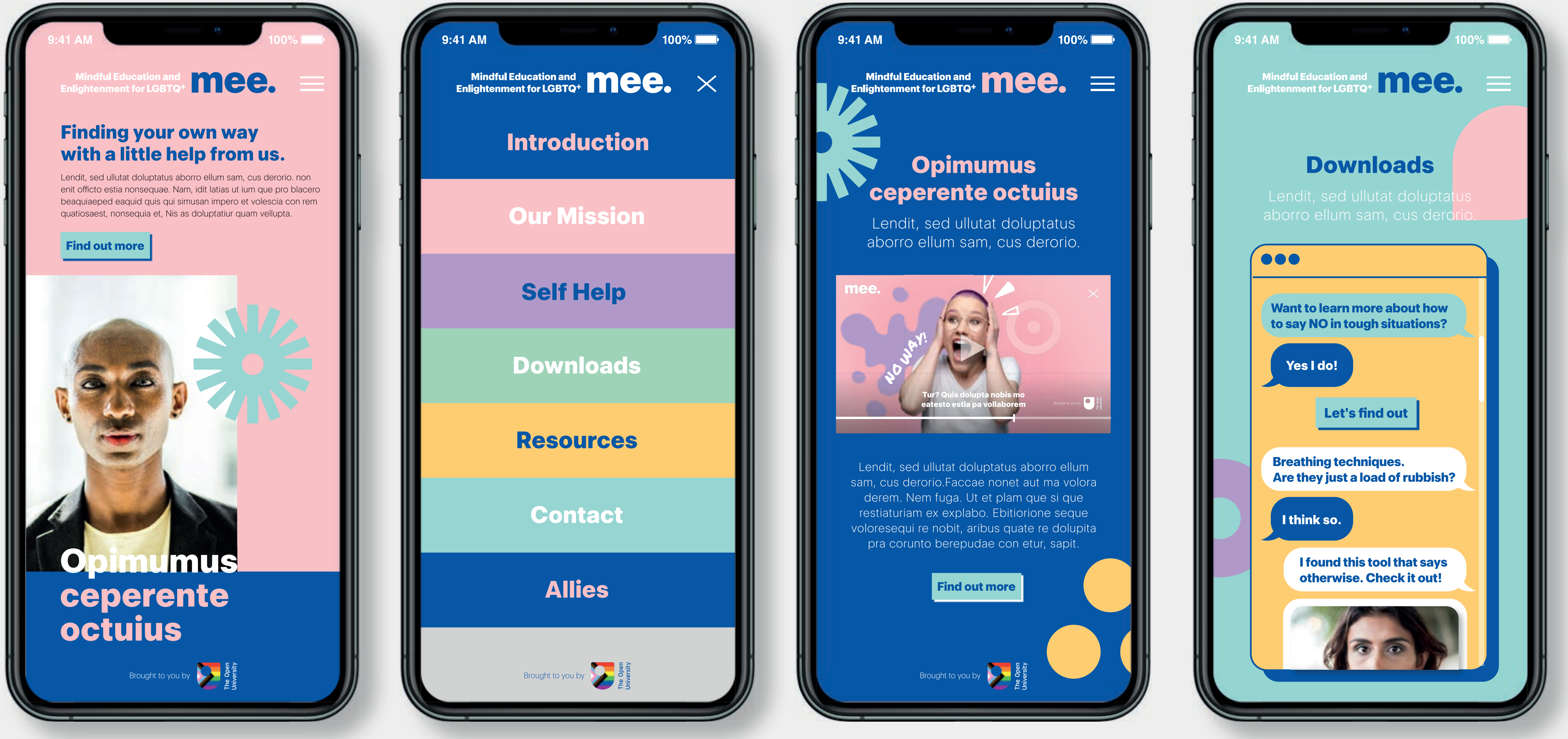

CONCEPT ONE

TOOLKIT ONLINE PLATFROM
(DESKTOP VIEW)

We start to introduce authentic photography to sit alongside the other branding elements, this adds to the genuine feel of the tooklit. This will enable us to showcase the various contributers, who will be discussing their own experiences and coping strategies. Contributors and other individuals are also depicted using illustration this gives us the ability to showcase diversity.

bluestep

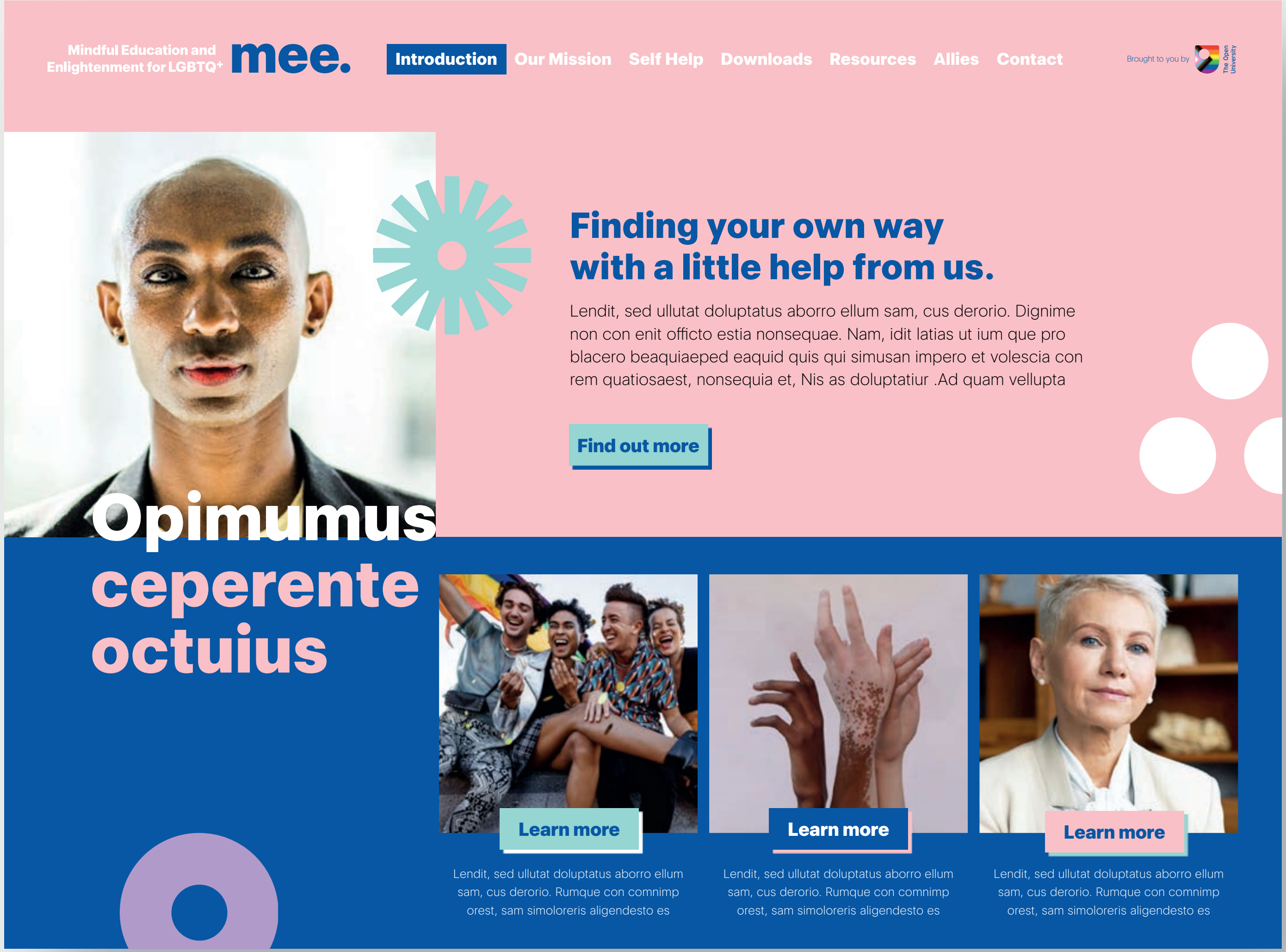

CONCEPT ONE

TOOLKIT VIDEO STYLING

The contributor videos use bright, clean, backgrounds utilising our colour pallet with a mix of hand drawn doodles, organic shapes, and geometric icons. These help express emotions and events being spoken about. The videos will be broken up using time stamps for ease of navigation.

bluestep

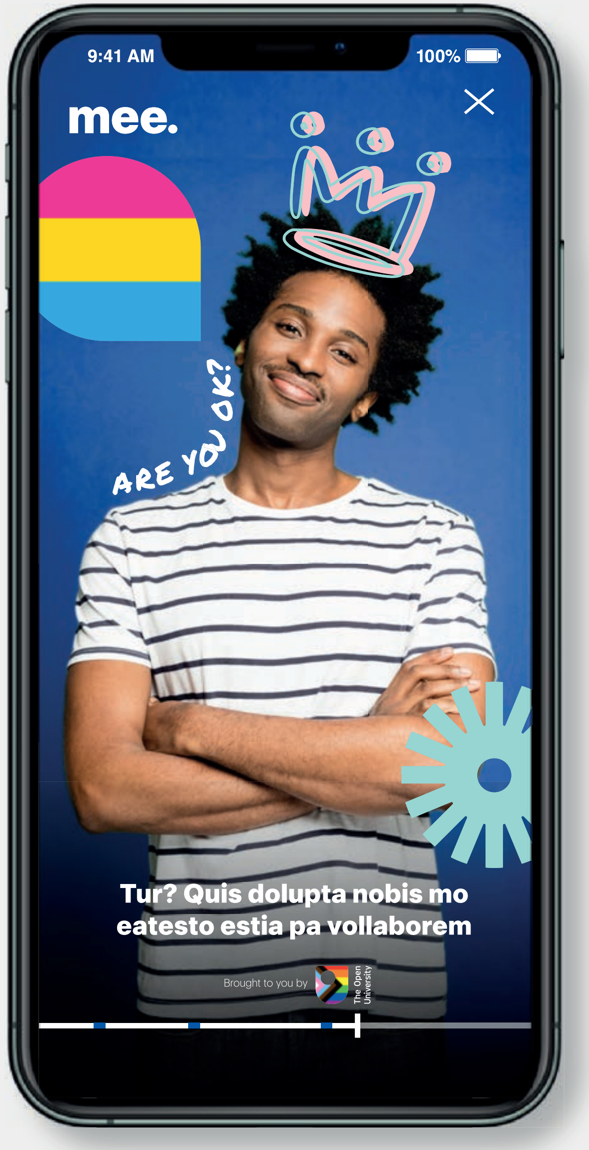

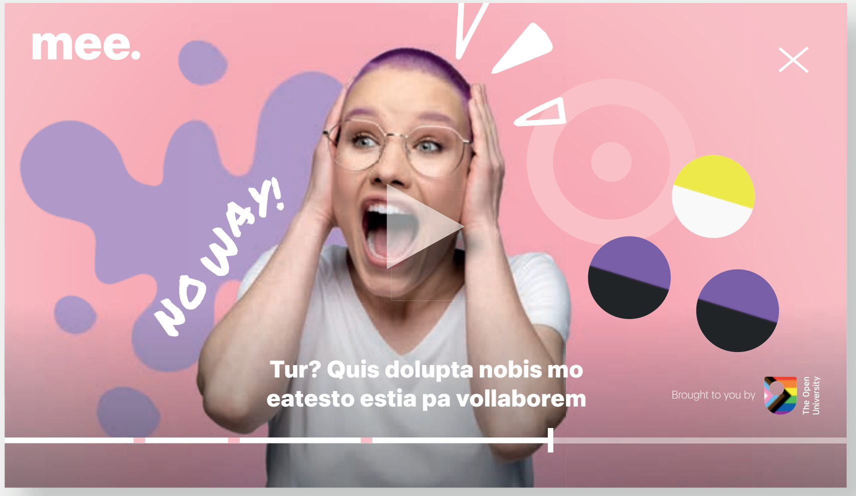

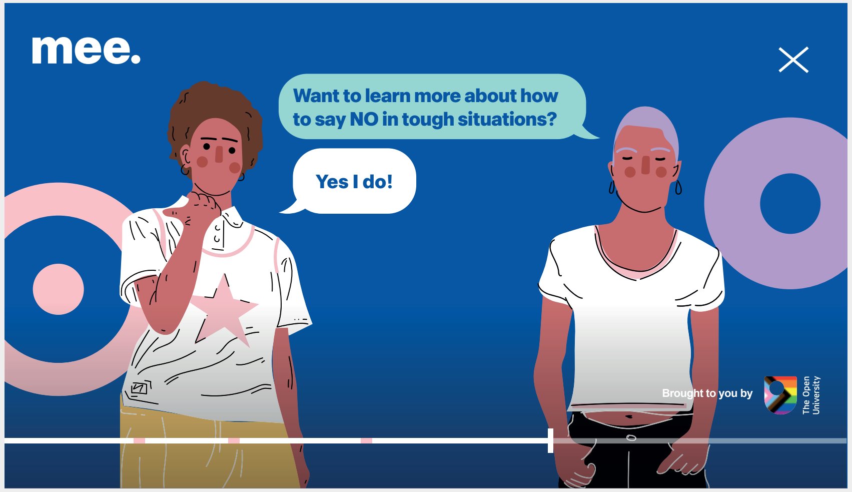

CONCEPT ONE

TOOLKIT SOCIAL ADS

Social ads were voted the most popular method of toolkit discovery by the young people. The identity translates well across ad space, allowing the use of authentic photography, bold headlines and the illustration style developed.

bluestep

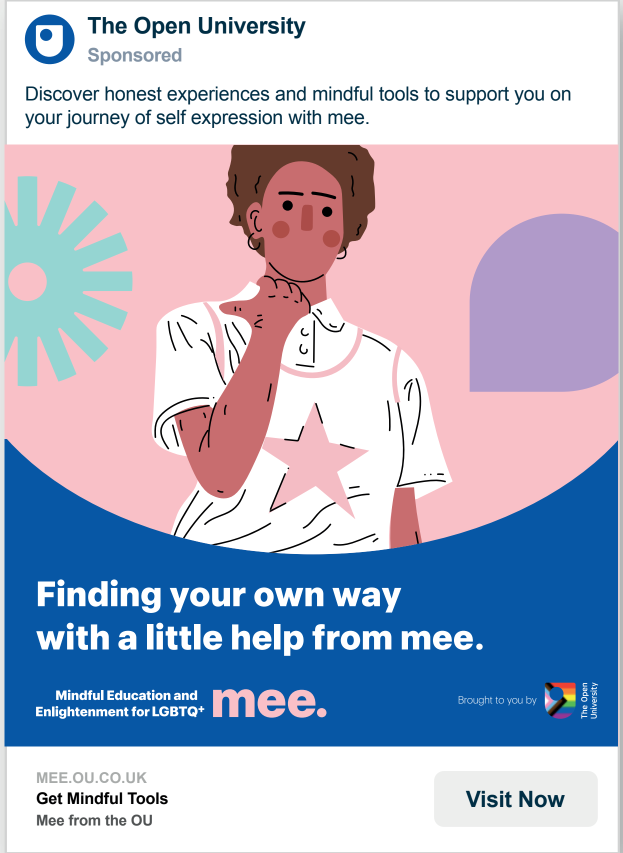

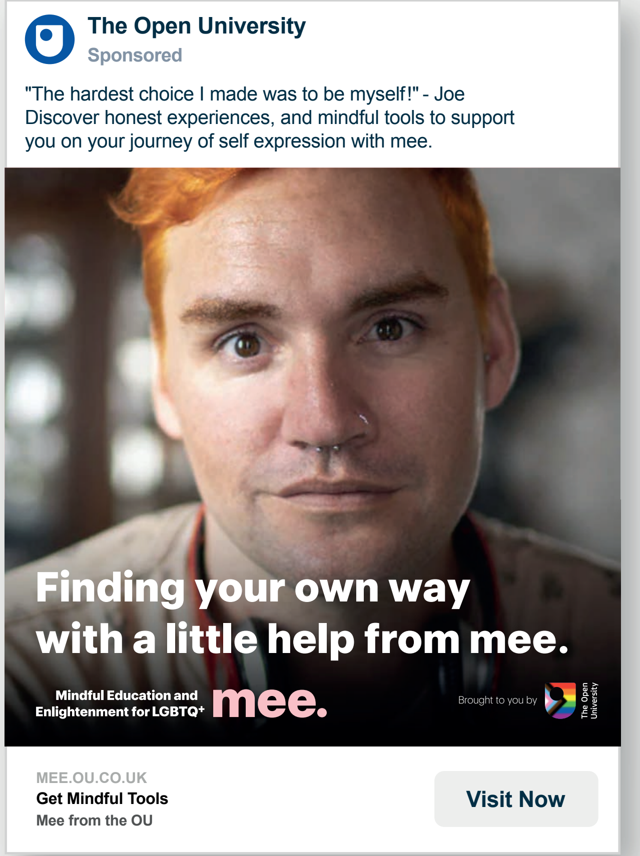

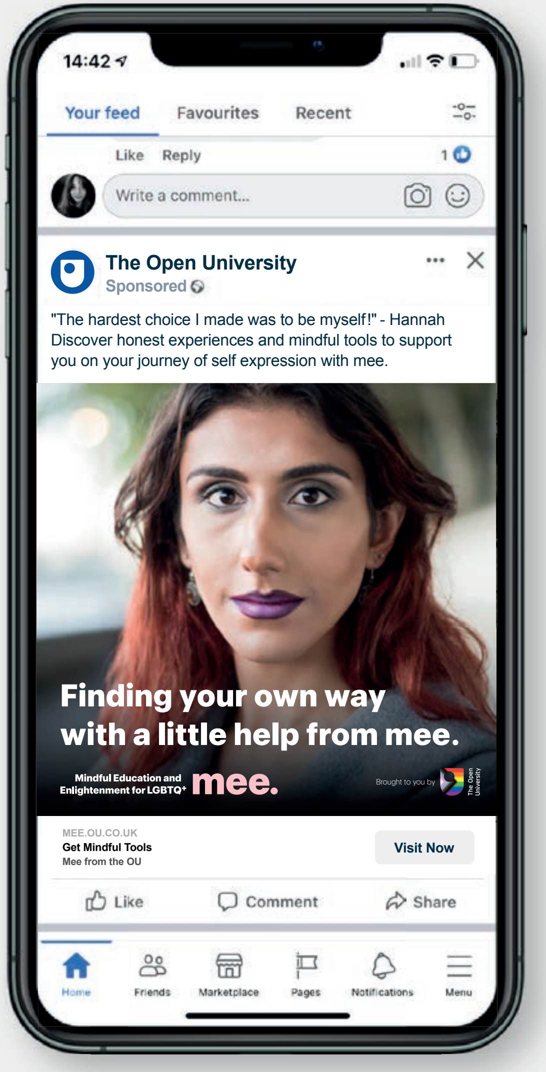

©2022 Copyright Bluestep Solutions

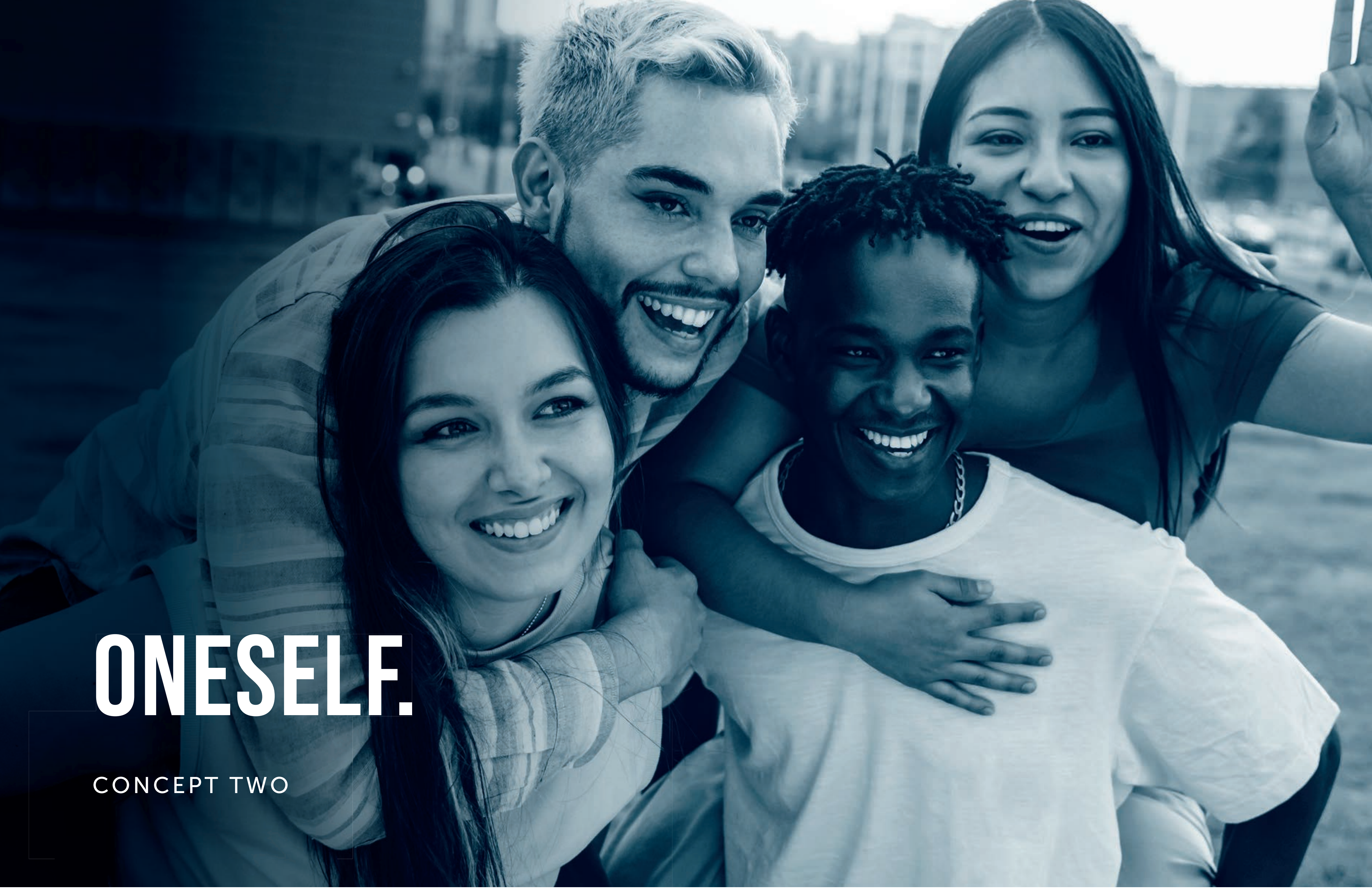

ONESELF.

CONCEPT TWO

CONCEPT TWO

TOOLKIT IDENTITY

The toolkit identity has been developed using organic shapes and typography. Focus is drawn to the identity name using stylistic speech bubble containers, representing the individuality of each self. This is the master identity shape.

bluestep

ONE SELF

DEFINED BY YOU.
ALLIED BY US.

Brought to you by The Open University

CONCEPT TWO

TOOLKIT IDENTITY

The identity is flexible and therefore can be used in other speech bubble containers to visually imply and represent individuality of self and expression.

bluestep

ONE SELF

DEFINED BY YOU. ALLIED BY US.

ONE SELF

DEFINED BY YOU. ALLIED BY US.

ONE SELF

DEFINED BY YOU. ALLIED BY US.

Brought to you by The Open University

CONCEPT TWO

TOOLKIT IDENTITY COLOURS

We have used the feedback from workshops to define the colour pallet. There was discussion around using the colour purple and bright colours to initiate positivity.

bluestep

25%
45%
75%

C: 76
M: 91
Y:0
K:0

R: 98
G: 52
B: 139

C: 0
M: 0
Y:0
K:100

R: 0
G: 0
B: 0

C: 0
M: 0
Y:0
K:0

R: 255
G: 255
B: 255

25%
45%

C: 75
M: 25
Y:0
K:0

R: 38
G: 153
B: 214

C: 5
M: 65
Y:0
K:0

R: 230
G: 122
B: 174

C: 0
M: 15
Y:75
K:0

R: 255
G: 216
B: 84

Brought to you by The Open University

CONCEPT TWO

TOOLKIT IDENTITY
FOR THE VISUALLY IMPARED

We've adapted some of the secondary colours to have more contrast, as these are generally the easiest to see because of their ability to reflect light. Solid, bright colors, such as red, orange and yellow are usually more visible than pastels.

bluestep

25%
45%

C: 76
M: 91
Y:0
K:0

R: 98
G: 52
B: 139

C: 0
M: 0
Y:0
K:100

R: 0
G: 0
B: 0

C: 0
M: 0
Y:0
K:0

R: 255
G: 255
B: 255

C: 55
M: 0
Y:70
K:0

R: 130
G: 192
B: 111

C: 5
M: 65
Y:0
K:0

R: 230
G: 122
B: 174

C: 0
M: 15
Y:75
K:0

R: 255
G: 216
B: 84

Brought to you by The Open University

CONCEPT TWO

TOOLKIT IDENTITY ICONS

A selection of shape containers have been developed for use within the toolkit identity. These add contrast, and act as components in which to house messaging and content.

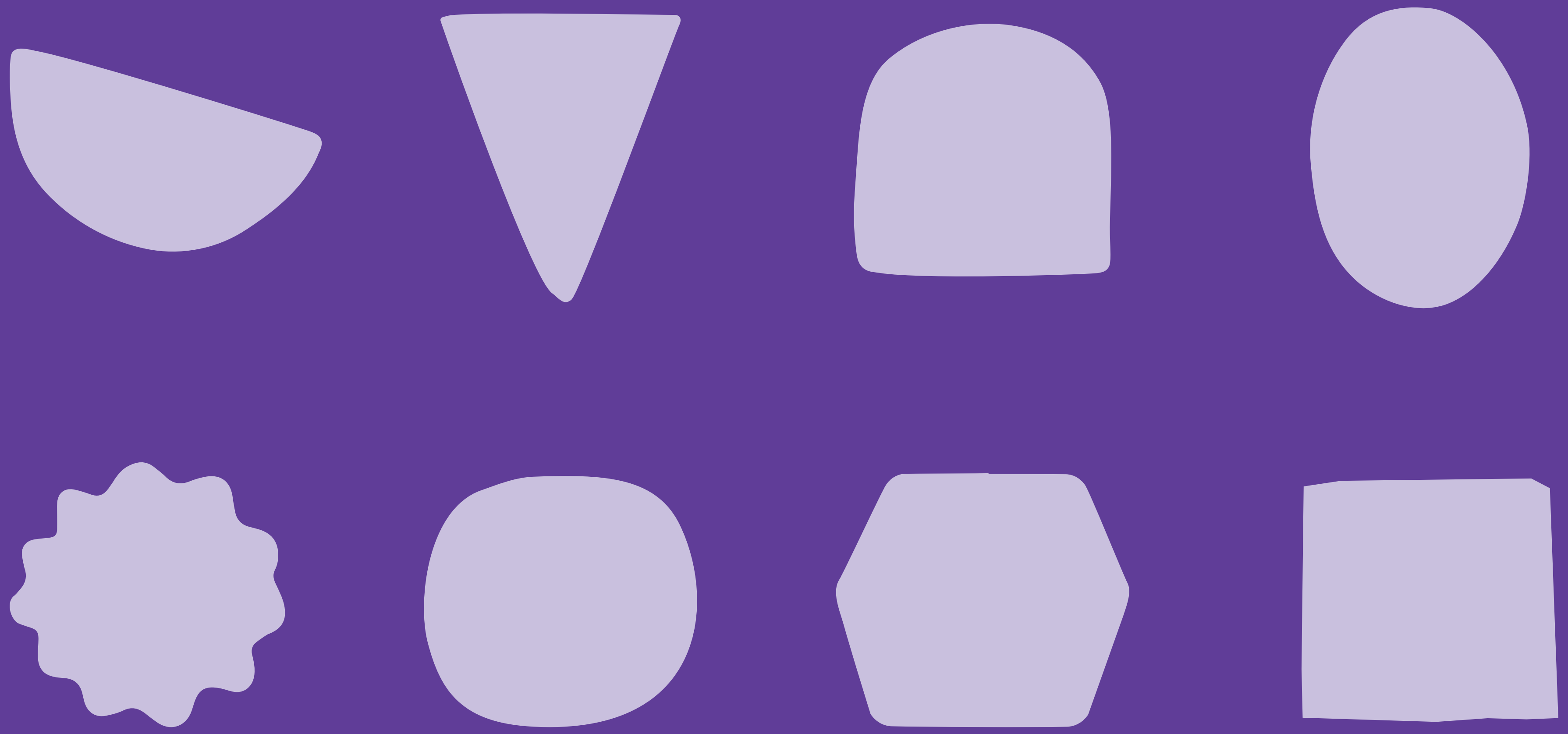

CONCEPT TWO

TOOLKIT ONLINE PLATFROM (MOBILE VIEW)

When combined, the elements of the tookit identity create an energetic, positive and welcoming platform for users to navigate. With bold captions and clear call to actions the brand elements are engaging for users.

bluestep

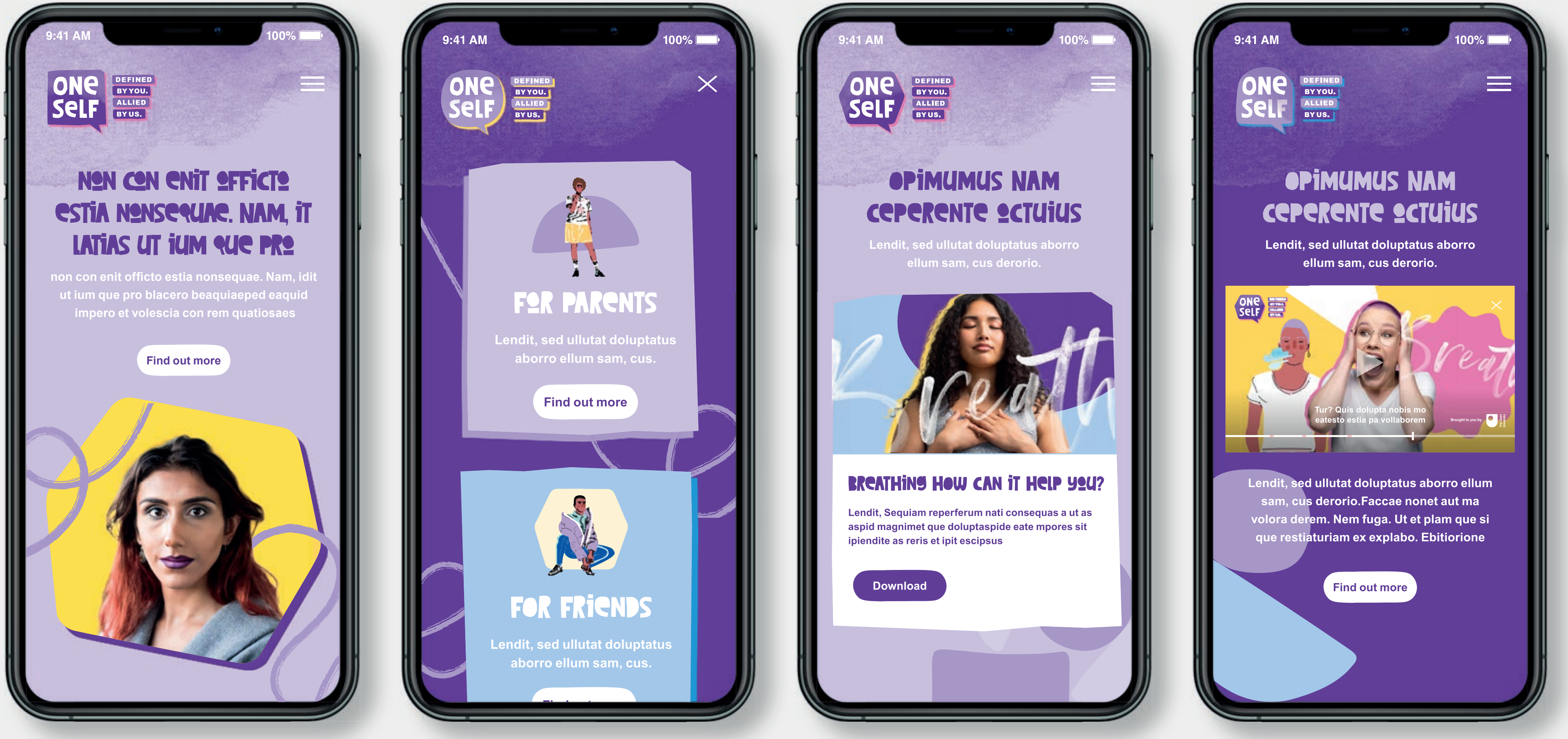

CONCEPT TWO

TOOLKIT ONLINE PLATFROM
(DESKTOP VIEW)

We start to introduce authentic photography to sit alongside the other branding elements, this adds to the genuine feel of the tooklit and will enable us to showcase the various contributors. Contributors and other individuals are also depicted using illustration this gives us the ability to create diversity.

bluestep

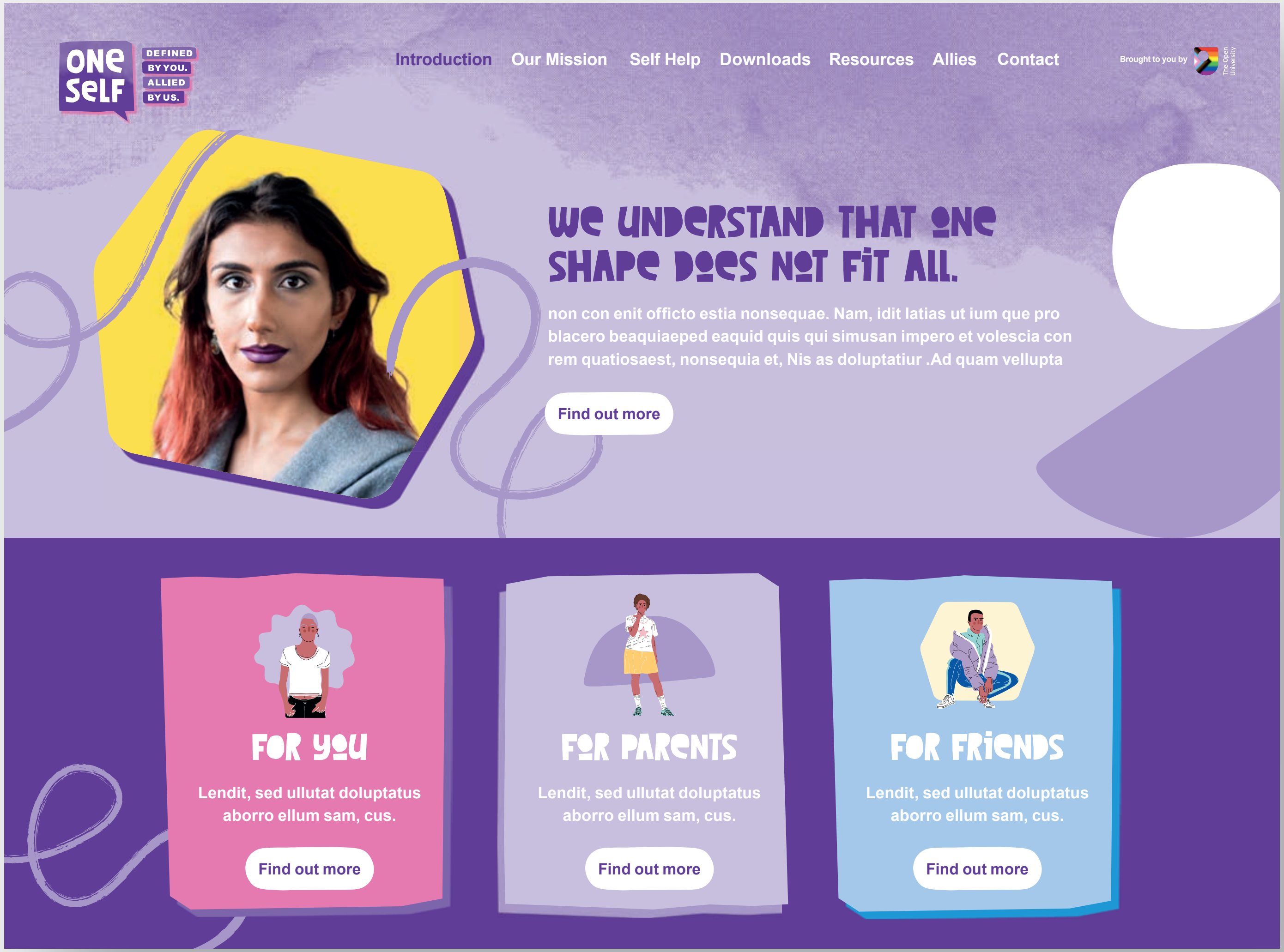

CONCEPT TWO

TOOLKIT VIDEO STYLING

The contributor videos use bright, clean, backgrounds utilising our colour pallet with a mix of hand drawn type, organic shapes, and the shape containers. These help express emotions and events being spoken about, especially with the use of the illustrations.

bluestep

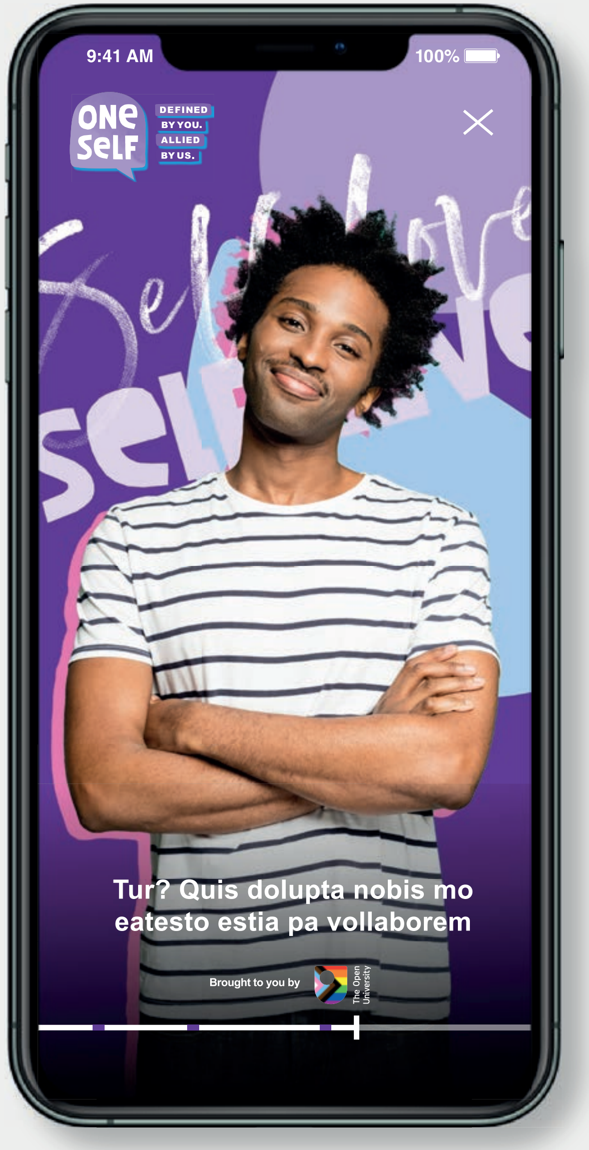

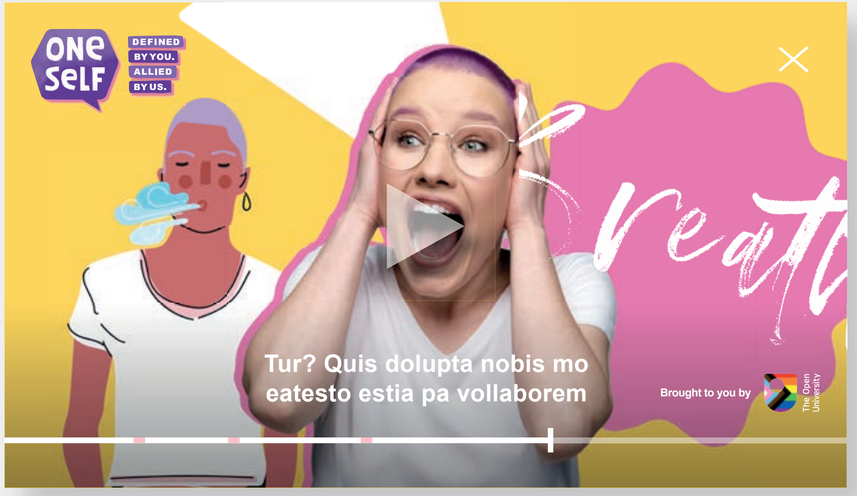

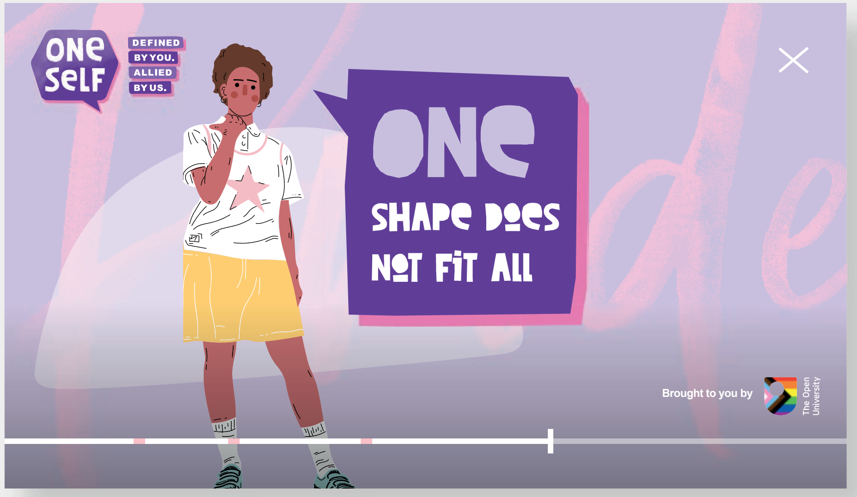

CONCEPT TWO

TOOLKIT SOCIAL ADS

Social ads were voted the most popular method of toolkit discovery by the young people. The identity translates well across ad space, allowing the use of authentic photography, bold headlines, and the illustration style developed.

bluestep

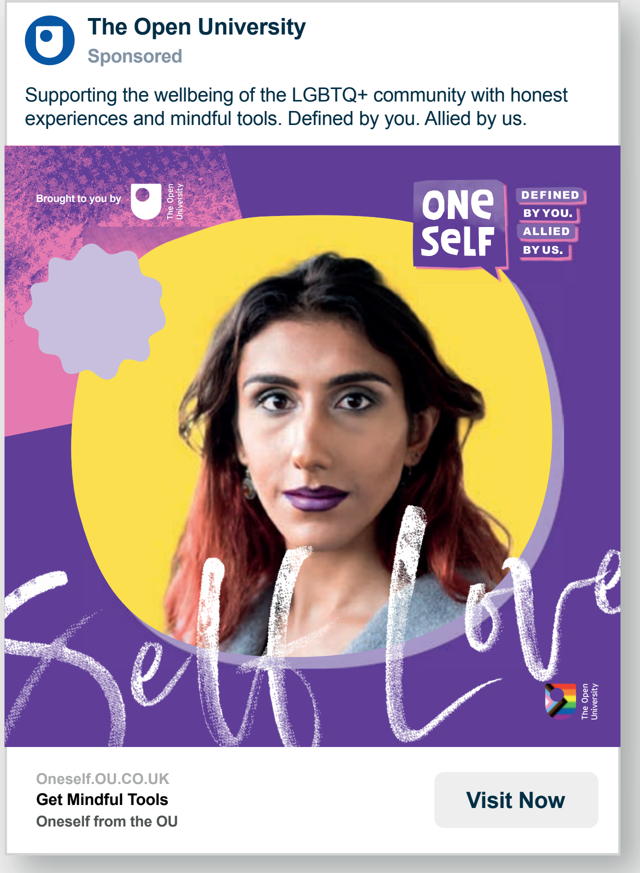

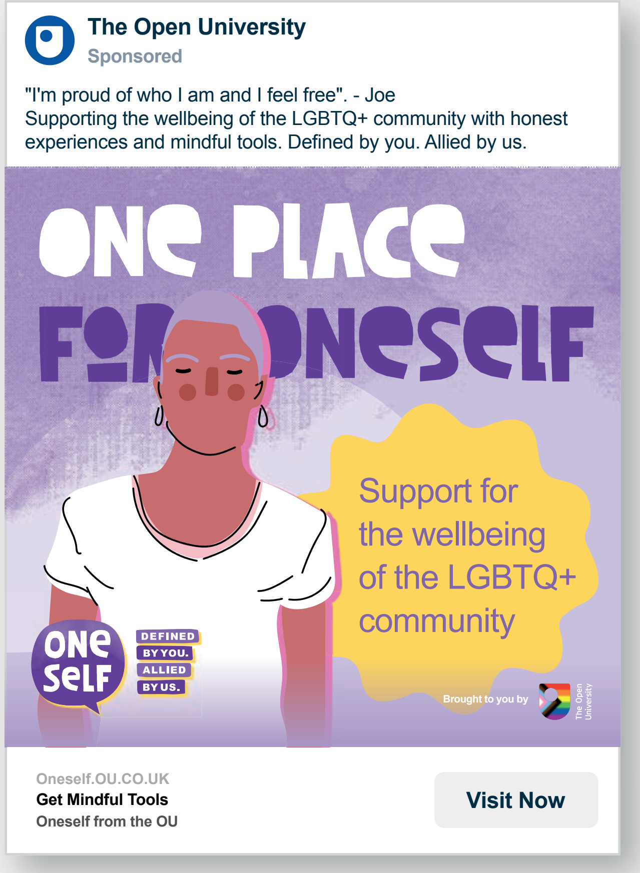

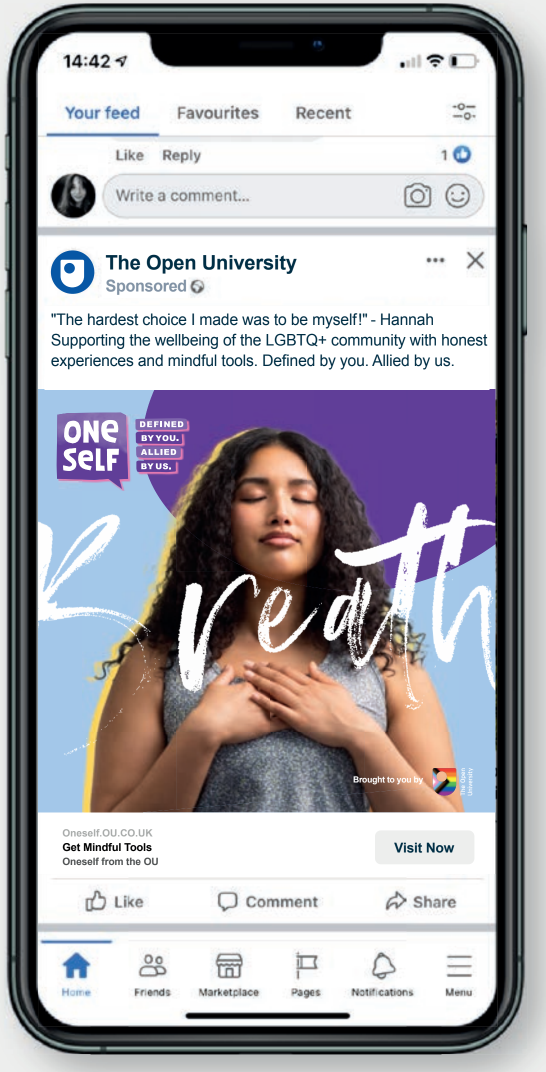

©2022 Copyright Bluestep Solutions

toolkit

NAMING

TOOLKIT NAMES

WITH SUPPORTING STRAPLINE

This name has been developed as an acronym. Not only does Mee refer to the individual, but also stands for 'mindful education & enlightenment' the premise of the toolkit for the LGBTQ+ young community.

bluestep

MEE.

MINDFUL EDUCATION & ENLIGHTENMENT FOR LGBTQ+

TOOLKIT NAMES

WITH SUPPORTING STRAPLINE

This name has been developed with individuality in mind. The reference of oneself as an individual being, defined by that individual alone rather than any external influences. The strapline simply implies this with the mention of support from their allies i.e the tooklit.

bluestep

ONESELF

DEFINED BY YOU. ALLIED BY US.

TOOLKIT NAMES

WITH SUPPORTING STRAPLINE

This name has been developed to imply freedom in oneself. It feels positive and encouraging. We underpin the name with a short statement about what the toolkit will offer for the young persons own individual journey.

bluestep

FREE TO BE

MINDFUL TOOLS FOR YOUR JOURNEY.

role model
PROFILES

CONTRIBUTOR

PROFILES

We have reached out to the below contributors from the LGBTQ+ community. These are not the final selection, as we are awaiting responses from other agancies including more information from those already approached.

bluestep

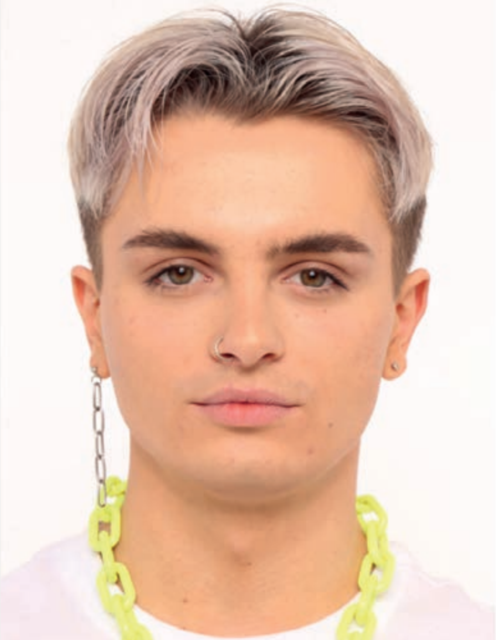
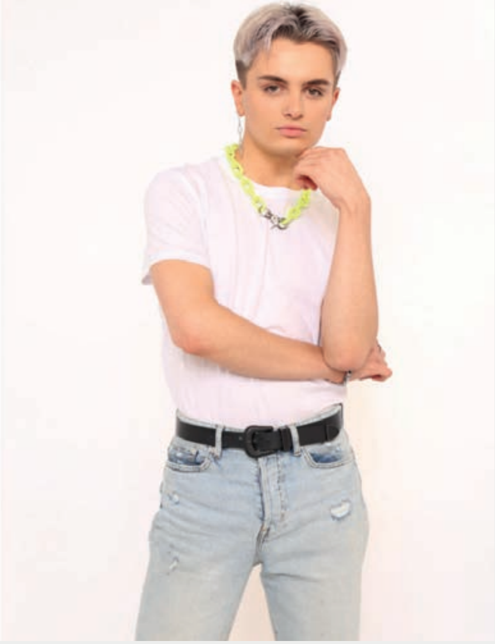

Callum Olver

Pronouns: He/Him

Sexuality: TBC

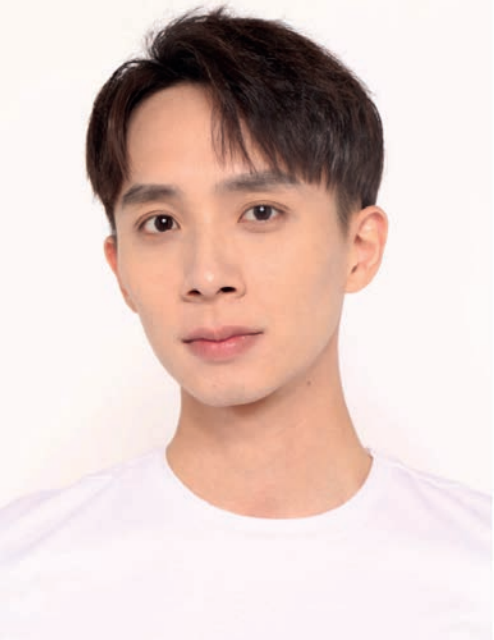
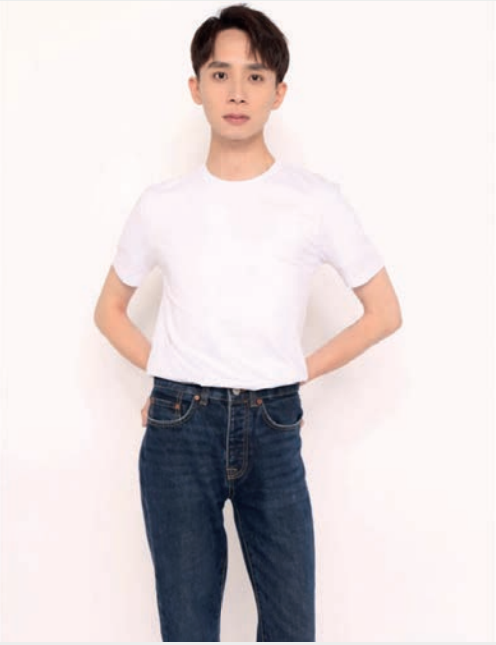

Dirk Tsai

Pronouns: He/Him

Sexuality: TBC

CONTRIBUTOR

PROFILES

We have reached out to the below contributors from the LGBTQ+ community. These are not the final selection, as we are awaiting responses from other agancies including more information from those already approached.

bluestep

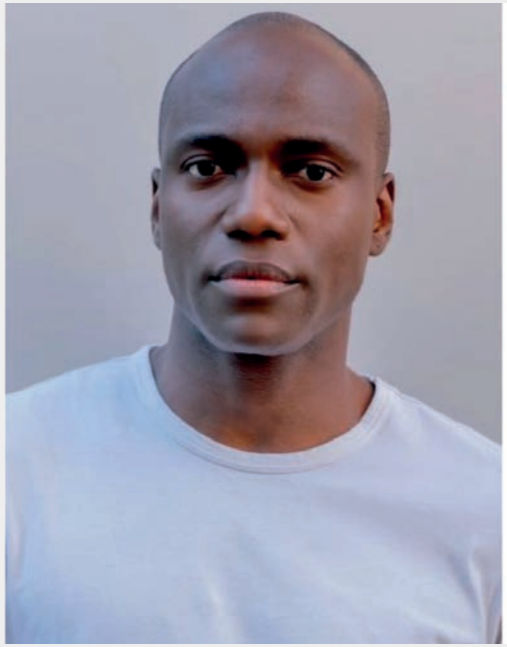
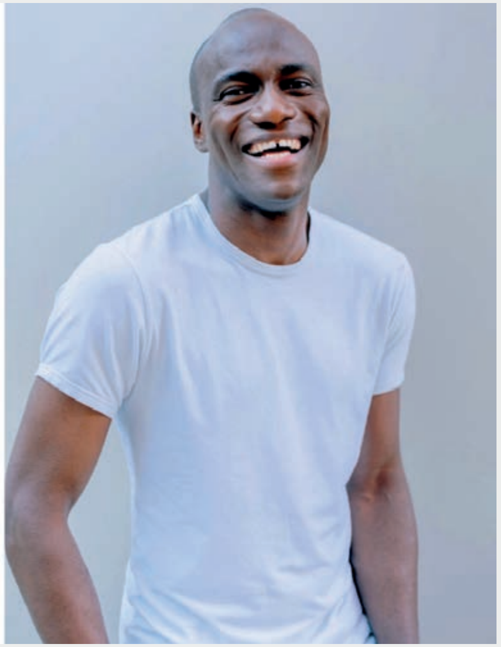

Julius Reuben

Pronouns: He/They

Sexuality: TBC

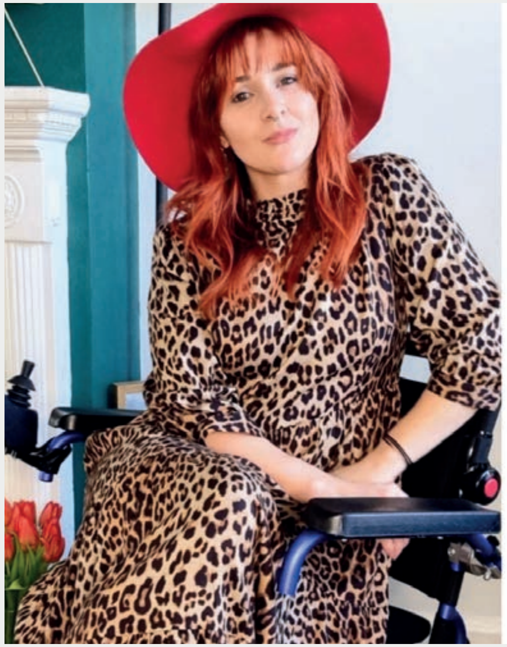
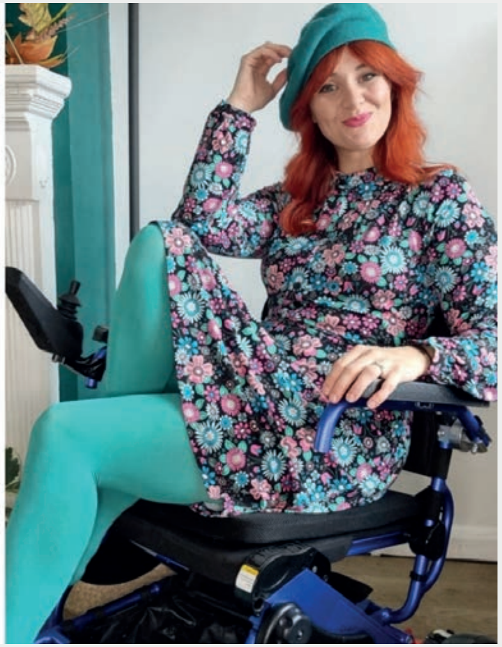

Gemma Bow

Pronouns: She/Her

Sexuality: Lesbian

CONTRIBUTOR

PROFILES

We have reached out to the below contributors from the LGBTQ+ community. These are not the final selection, as we are awaiting responses from other agancies including more information from those already approached.

bluestep

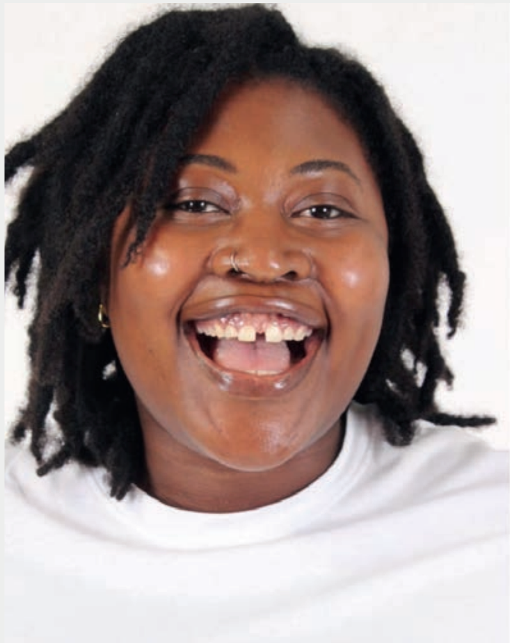

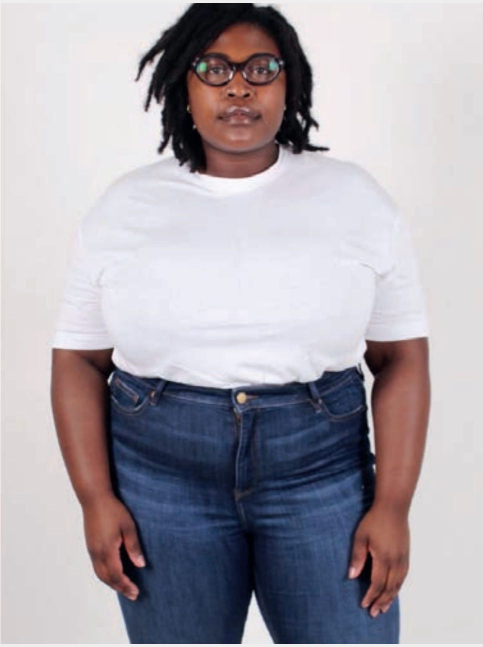

Ruvimbo

Pronouns: She/Her

Sexuality: TBC

thank you
FOR YOUR TIME

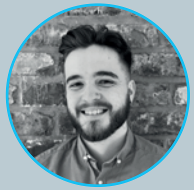

Mark Craig
Creative Account Manager

mark.craig@bluestepsolutions.com

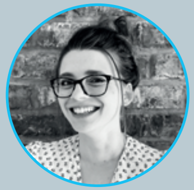

Harley Jacquest
Lead Creative

harley.jacquest@bluestepsolutions.com

bluestep
Design & Merchandise
