# Supplementary material for: A Web-Based Intervention to Support the Mental Well-Being of Sexual and Gender Minority Young People: Mixed Methods Co-Design of Oneself
Source: JMIR Form Res. 2024 May 21;8:e54586. doi: 10.2196/54586 (PMC11150889; doi:10.2196/54586)
Supplement: Multimedia Appendix 2 [file formative_v8i1e54586_app2.pdf]

Multimedia Appendix 2

THE PRIDE PROJECT: CO-DEVELOPING A NEW DIGITAL TOOLKIT WITH LGBTQ+ YOUNG PEOPLE (WORKSHOP QUESTIONNAIRE SUMMARY)
(N=15 OR 16)

I. From the six images below, marked A-F, please circle which overall look you think would appeal the most to LGBTQ+ young people using the toolkit?

A n=3, B n=9 and D n=3 (the others received zero votes)

Figure 1

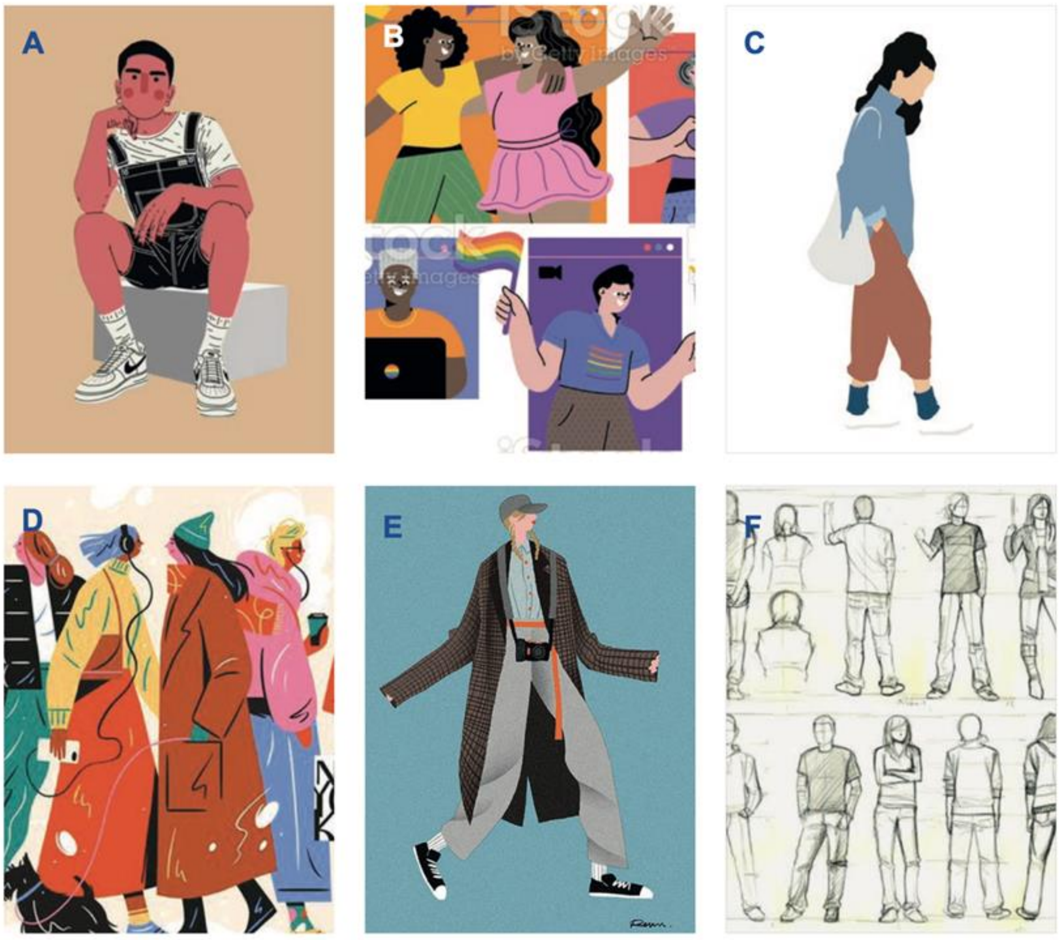

II. What features do you think will be important that we include in the toolkit (please circle as many as you think will be useful or important):

- Downloadable information sheets n=13
- Animations n=9
- Real people talking about their experiences n=11
- Actors re-creating realistic every-day situations n=7
- Choose-your-own-adventure videos n=4
- Subtitles/written text for all video clips n=13

III. What device are LGBTQ+ young people most likely to access the toolkit on (please circle one response below)?

- Desktop computer/PC n=1
- Tablet (like an iPad) n=0
- Smart/mobile phone n=15

IV. How likely is it, if you were using the toolkit, that you would listen to the video content using headphones?

1 = Extremely likely n=3

2 = Very likely n=8

3 = Not sure n=2

4 = Unlikely n=1

5 = Not at all likely n=1

V. Ideally, how long should each video clip be in the toolkit (please circle one response)?

- 15 seconds or less n=0
- Between 16 and 30 seconds n=4
- Between 31 and 60 seconds n=7
- Between 1 and 3 minutes n=2
- Between 3 and 5 minutes n=2
- Longer than 5 minutes n=0

VI. How would you prefer to hear about the Toolkit (please feel free to circle more than one response)?

- Social media ads n=12
- Email n=4
- Leaflet drop n=4
- School/college services n=6
- Your GP surgery n=2
- LGBTQ+ youth workers n=11
- Another young person n=9
- Someone or somewhere else (e.g., an LGBTQ+ organisation), in particular n=6 ______________________

VII. Who do you want to see sharing advice to LGBTQ+ young people in the toolkit (please feel free to circle more than one response)?

- Actors (who are LGBTQ+) n=11
- Regular LGBTQ+ individuals sharing their personal experiences n=13
- LGBTQ+ influencers/public figures n=15
- Creative and illustrative animations with LGBTQ+ voice actors n=14
- A combination of animation and 'live action' (actors or real people) n=5

VIII. What colour/s do you feel best represent LGBTQ+ young people and should be in the toolkit? Any colours we should avoid? Various answers to be collated.

____

IX. Where and when do you think LGBTQ+ young people would want to access a toolkit to support their wellbeing? Various answers to be collated.
