# Supplementary material for: A Web-Based Intervention to Support the Mental Well-Being of Sexual and Gender Minority Young People: Mixed Methods Co-Design of Oneself
Source: JMIR Form Res. 2024 May 21;8:e54586. doi: 10.2196/54586 (PMC11150889; doi:10.2196/54586)
Supplement: Multimedia Appendix 3 [file formative_v8i1e54586_app3.pdf]

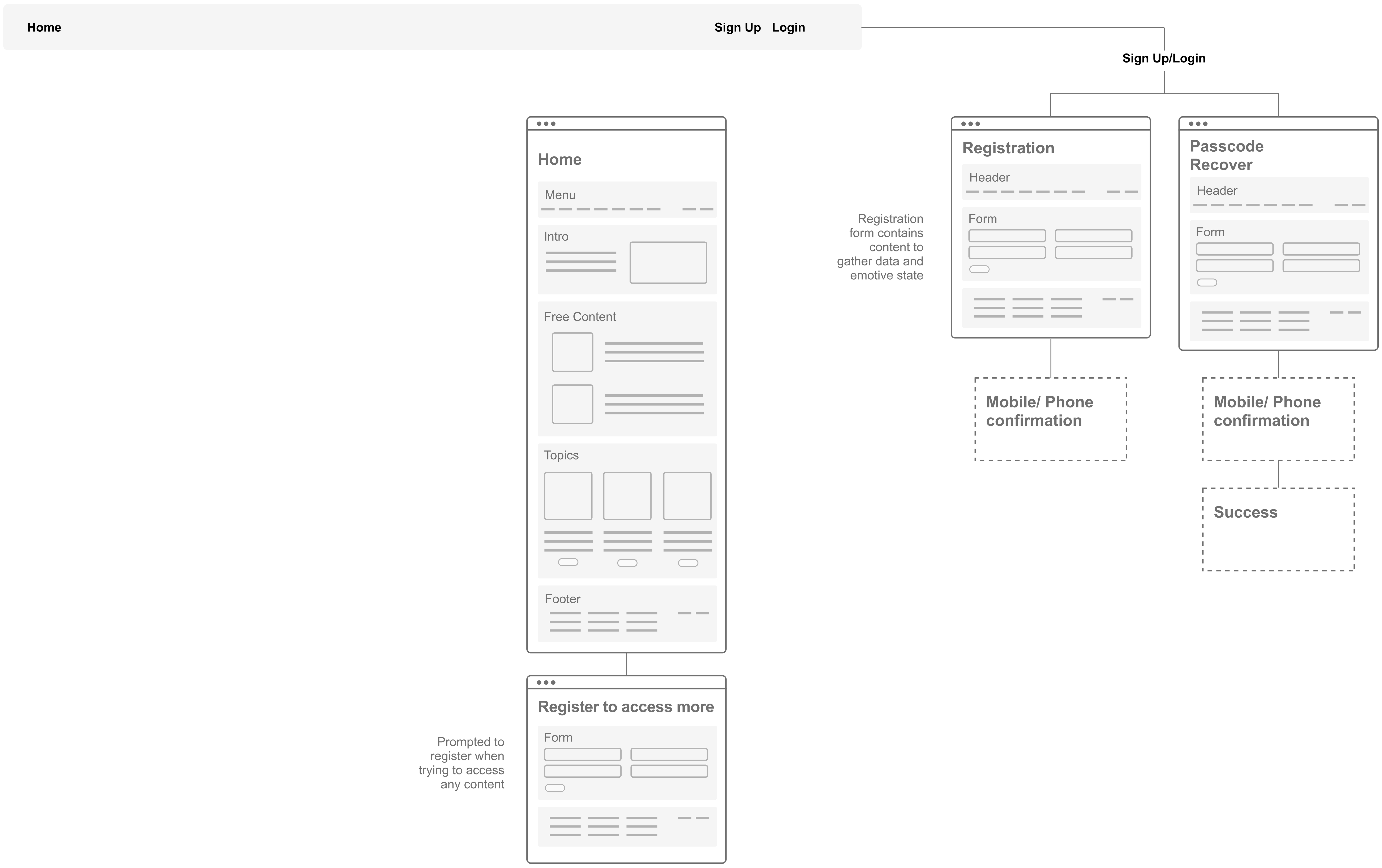
Home
Sign Up
Login
Sign Up/Login
Home
Menu
Intro
Free Content
Topics
Footer
Register to access more
Form
Prompted to register when trying to access any content
Registration
Header
Form
Registration form contains content to gather data and emotive state
Passcode Recover
Header
Form
Mobile/ Phone confirmation
Mobile/ Phone confirmation
Success

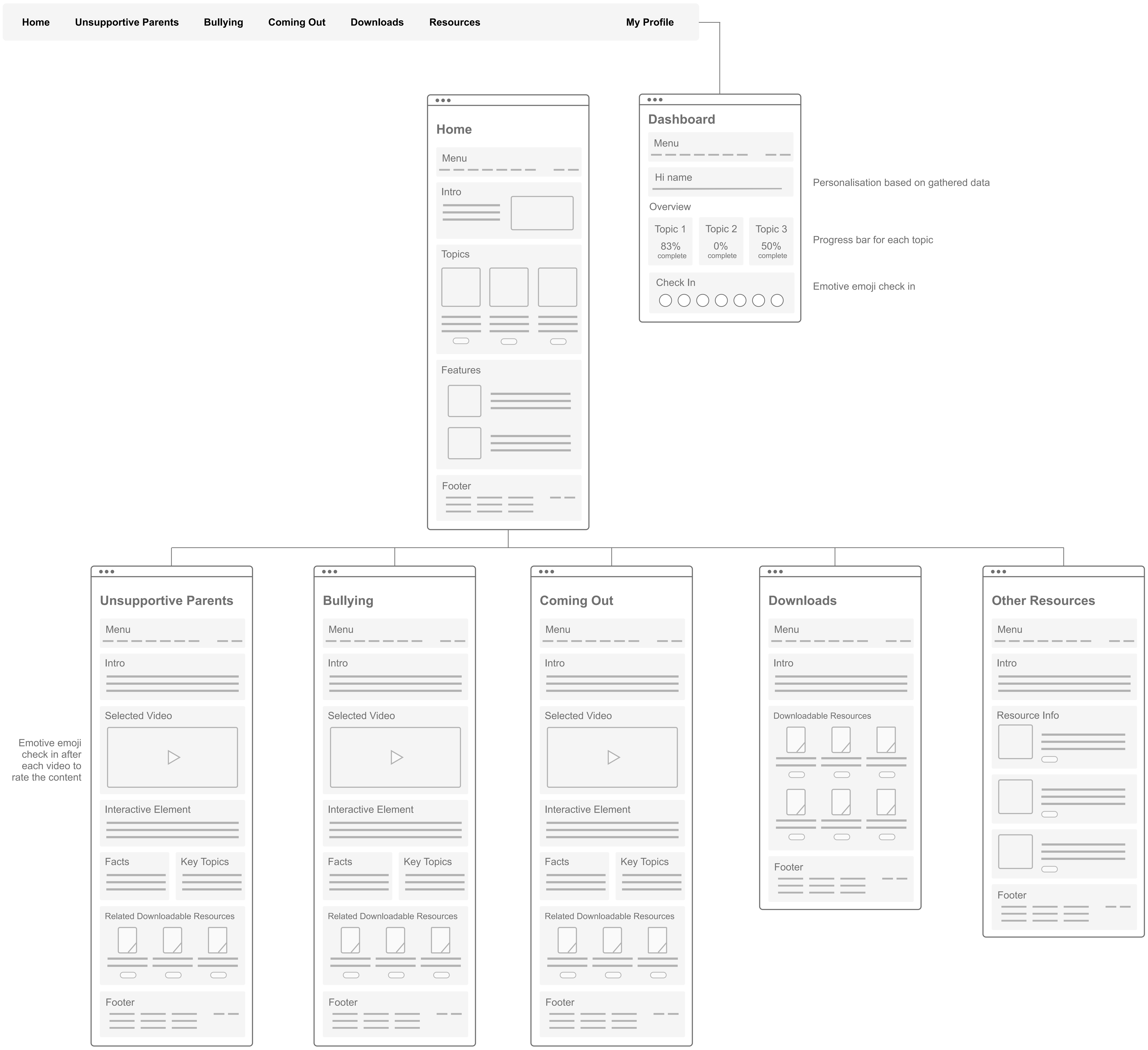
Home
Unsupportive Parents
Bullying
Coming Out
Downloads
Resources
My Profile
Home
Menu
Intro
Topics
Features
Footer
Dashboard
Menu
Hi name
Overview
Topic 1
83%
complete
Topic 2
0%
complete
Topic 3
50%
complete
Check In
Personalisation based on gathered data
Progress bar for each topic
Emotive emoji check in
Unsupportive Parents
Menu
Intro
Selected Video
Interactive Element
Facts
Key Topics
Related Downloadable Resources
Footer
Emotive emoji check in after each video to rate the content
Bullying
Coming Out
Downloads
Downloadable Resources
Other Resources
Resource Info
