# Supplementary material for: A Web-Based Intervention to Support the Mental Well-Being of Sexual and Gender Minority Young People: Mixed Methods Co-Design of Oneself
Source: JMIR Form Res. 2024 May 21;8:e54586. doi: 10.2196/54586 (PMC11150889; doi:10.2196/54586)
Supplement: Multimedia Appendix 4 [file formative_v8i1e54586_app4.pdf]

Multimedia Appendix 4

Oneself – Approach to AV clips

Youth-friendly and inclusive language, storytelling narrative. 2-3 minutes clips. Authentic, from the heart, hope-instilling.

Prompts

Overall context

How and have and do you cope with LGBTQ+ mistreatment? Split your answer across examples from your home, school and coming out story.

Social environments will change but do so slowly. How can young people cope with this? How did you cope with this when you were a teenager at home with your family, in high school or whilst coming out? What made it easier for you?

Teenagers do not have the power that adults do. How did you manage this in your everyday life as a teenager? As a young person, how did you cope when realising that the world could be a challenging place for LGBTQ+ youth? How did you adjust your expectations when realising that some people and spaces might never be fully accepting?

How can LGBTQ+ teenagers take on an educator role and mentor other LGBTQ+ young people? For example, how can LGBTQ+ youth get involved in helping others?

Some 'righteous anger' is powerful. It could be helpful to accept this. How do you think LGBTQ+ young people can use this anger to bring about positive change (e.g. activism)?

LGBTQ+ young adults often reflect back and think that their earlier experiences strengthened them. In what ways have your experiences as a teenager made you a more resilient person?

Coming out

- Tell us your experience of coming out as a teenager. How old were you? Who did you first tell? What was that like? Could you share some of your most positives experiences coming out? What about some of your most challenging experiences when coming out?
- The internet has been described by LGBTQ+ young people as lifesaving. How can LGBTQ+ young people skilfully use the internet, especially whilst coming out?
- How have you used the internet to find others like yourself online and support you whilst coming out?
- As a young person, have you ever been outed? What were the consequences? How can being outed be best managed?
- It is very important to feel safe with the first few people you come out to. So how do you know who it is safe to disclose to?
- How can young people introduce that fact that they are LGBTQ+ in social situations?
- Does a young person have to come out?
- What can help or what should a young person do if/when someone reacts negatively when they come out to them?

Family and friends

- How did friends and family react to you being LGBTQ+ as a teenager? Has this changed over time?
- Why do some friends and family members struggle accepting and supporting LGBTQ+ young people?
- What are some of the positive messages that family and friends can tell LGBTQ+ young people?
- It can be helpful to do things you enjoy doing to take the edge off how you feel when things get difficult with friends or family. What activities did you do, or do you still do, that help you to cope and feel well (or feel better)?
- At times when your friends and family were not supportive of you as an LGBTQ+ young person, where could you have got support from? Did anything work for you?

School bullying

- What was your experience of high school like as an LGBTQ+ young person?
- If you had a magic wand, what would you do to make schools better for LGBTQ+ young people?
- Can you describe an experience of LGBTQ+-related bullying at school? What worked that helped you manage the bullying?
- In your experience how did teachers and students react to LGBTQ+ youth being bullied at school?
- In an ideal world, how should teachers and students manage the bullying of LGBTQ+ youth?
