# Supplementary material for: A Web-Based Intervention to Support the Mental Well-Being of Sexual and Gender Minority Young People: Mixed Methods Co-Design of Oneself
Source: JMIR Form Res. 2024 May 21;8:e54586. doi: 10.2196/54586 (PMC11150889; doi:10.2196/54586)
Supplement: Multimedia Appendix 5 [file formative_v8i1e54586_app5.pdf]

Multimedia Appendix 5 – A Oneself topic example: Coming Out

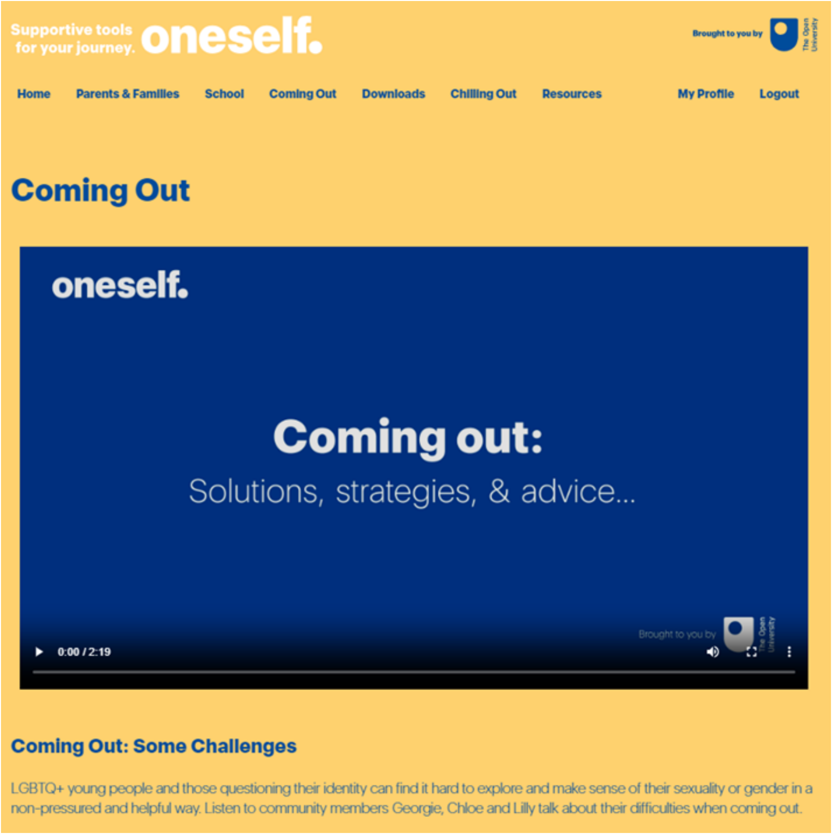

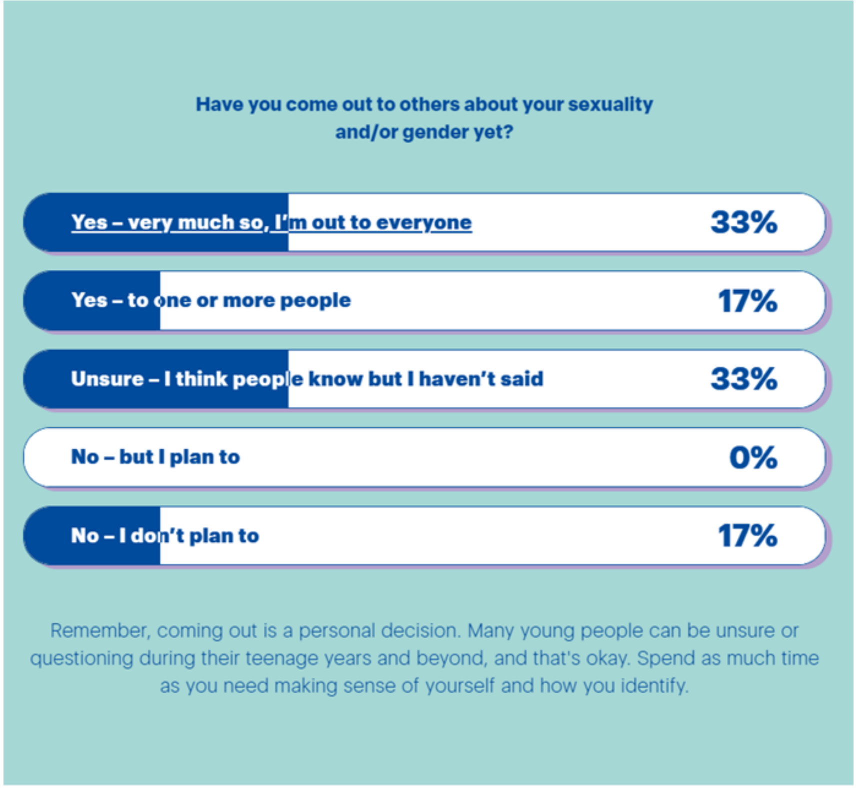

"You don't have to come out if you don't want to. If you're being pressured to come out by your peers, you don't have to listen. Coming out is a personal journey."

– LGBTQ+ YOUTH CONSULTANT FOR ONESELF

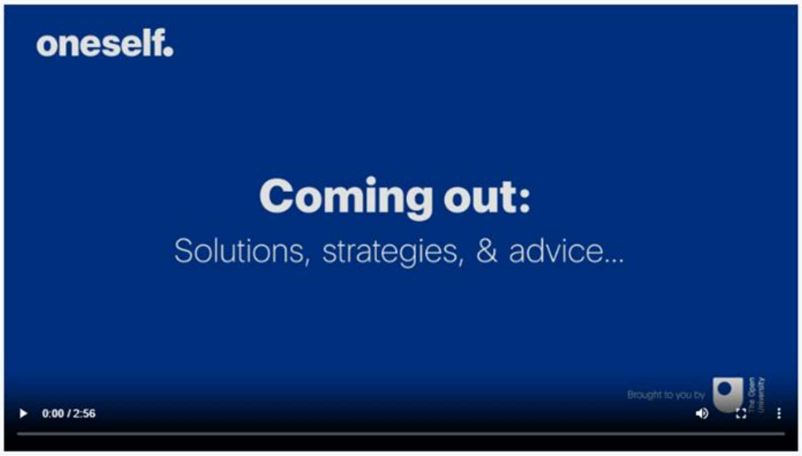

Coming Out: Some Strategies

Listen to community members Georgie, Chloe and Lilly talk about coming out and how they came to better understand themselves and who to confide in.

Which of the three experiences was most like your own?

Georgie's	17%
Chloe's	0%
Lilly's	17%
None of them seem like my own experience	67%

All our coming out experiences will vary, many people find that coming out is quite a relief. But some people never come out and that's okay too.

Your Reflections

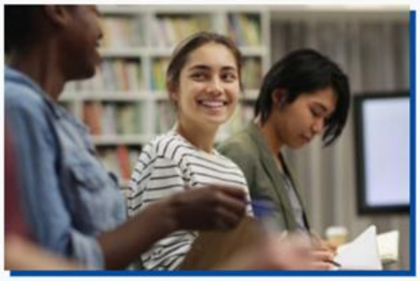

There are different ways of looking at the stages around coming out. Here are five such steps.

Explore More

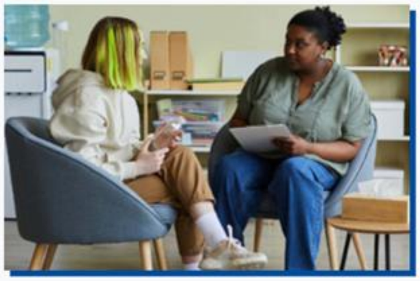

Have you used any of these responses or strategies when coming out?

Explore More

Downloadable Resources

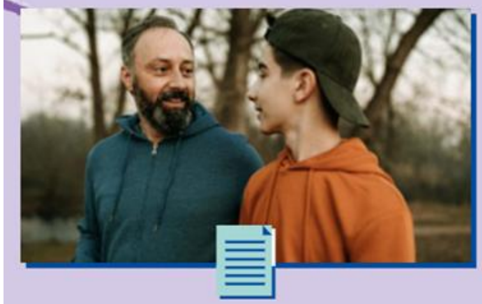

Thinking about Coming Out?

Read this factsheet to help you with your coming out journey.

Download

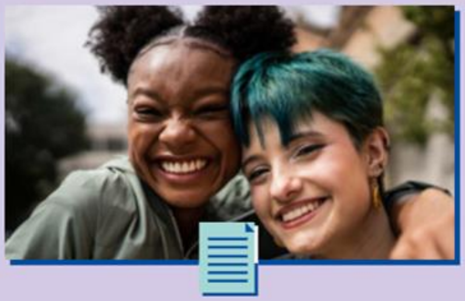

Finding Hope

Read this factsheet to help you envision you living your best life.

Download
